# SOMOphilic Alkynylation of Unreactive Alkenes Enabled by Iron-Catalyzed Hydrogen Atom Transfer

**DOI:** 10.3390/molecules27010033

**Published:** 2021-12-22

**Authors:** Binlin Zhao, Tianxiang Zhu, Mengtao Ma, Zhuangzhi Shi

**Affiliations:** 1 Department of Chemistry and Material Science, College of Science, Nanjing Forestry University, Nanjing 210037, China; ztx_126mm@163.com (T.Z.); mengtao@njfu.edu.cn (M.M.); 2 State Key Laboratory of Coordination Chemistry, Chemistry and Biomedicine Innovation Center (ChemBIC), School of Chemistry and Chemical Engineering, Nanjing University, Nanjing 210093, China; shiz@nju.edu.cn

**Keywords:** alkynylation, iron-catalysis, alkenes, hydrogen atom transfer, radical

## Abstract

We report an efficient and practical iron-catalyzed hydrogen atom transfer protocol for assembling acetylenic motifs into functional alkenes. Diversities of internal alkynes could be obtained from readily available alkenes and acetylenic sulfones with excellent Markovnikov selectivity. An iron hydride hydrogen atom transfer catalytic cycle was described to clarify the mechanism of this reaction.

## 1. Introduction

**Scheme 1:**
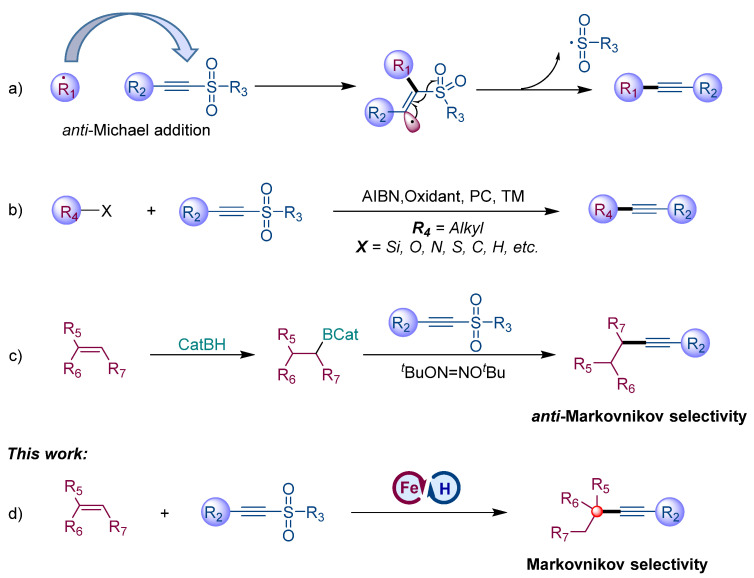
Strategies towards SOMOphilic alkynylation. (**a**) Radical-mediated alkynylation utilizing acetylenic sulfones as alkynyl sources; (**b**) SOMOphilic alkynylation via varieties of radical-mediated strategies; (**c**) *anti*-Markovnikov selective alkynalation of alkenes with acetylenic sulfones; (**d**) Iron-catalysed Markovnikov selective alkynalation.

Alkyne and its derivatives are important structural cores in diversities of bioactive compounds from natural products to pharmaceuticals and functional materials [1,2,3], which also serve as versatile synthetic building blocks in organic synthesis [4,5,6,7,8,9]. As a result, remarkable attention has been paid to the synthesis of these prime frameworks from versatile feedstocks. Straightforward nucleophilic or electrophilic alkynylation of nucleophilic acetylides generated utilizing strong bases relying on their intrinsic acidity or electrophilic acetylide variants prepared through complex routes were considered as traditional strategies to assemble the alkyne moieties onto the organic skeletons for the construction of C (*sp*^3^)–C (*sp*) bonds. Additionally, C (*sp*^3^)–C (*sp*) bond coupling reactions by the catalysis of transition metals serve as powerful methods for the construction of alkynes, wherein some appropriate ligands were employed to restrict the *β*-elimination of alkyl–metal complexes [10,11,12,13]. Recently, radical-mediated SOMOphilic alkynylation has made remarkable progress depending on the flourish development of radical chemistry, which also provides reliable approaches for the formation of C (*sp*^3^)–C (*sp*) bond. Moreover, diversities of alkyne reagents were designed and synthesized, providing alternative alkyne precursors to enable alkynyl functionalization [14,15,16,17,18,19,20,21,22,23,24,25,26]. Among these, acetylenic sulfones [22,23] exhibited vigorous synthetic abilities in organic transformations, especially forming C (*sp*^3^)–C (*sp*) bonds via a radical-induced process. Generally, acetylenic sulfones are usually treated as efficient radical acceptors, attached by the generated carbon radicals with excellent anti-Michael selectivity to afford enyl radical intermediates, achieving alkynyl functionalization with the realization of a sulfonyl radical via a sequential radical-mediated *β*-scission process (Scheme 1a). These reactions were amply explored by the efforts of organic chemical scientists (Scheme 1b). Chen [27] and König [28] developed photo-induced decarboxylative alkynylations of redox-active esters using acetylenic sulfones as alkynyl sources under reductive photochemical conditions, respectively. In 2016, Zhu and coworkers reported that the ring opening alkynylation of strained cyclobutanols could be enabled by oxygen radical-induced C–C bond cleavage by the catalysis of manganese salts [29]. In addition, another visible light-promoted oxygen radical-induced ring opening alkynylation via C–C bond cleavage was disclosed by the group of Wang [30]. Meanwhile, Fu and coworkers demonstrated that the alkynyl motifs from acetylenic sulfones could be introduced onto the aliphatic alcohol-derived redox-active esters, affording alkynes bearing quaternary carbons via a photo-induced C–O bond cleavage [31]. Additionally, aliphatic amine-derived Katritzky salts were employed by Gryko and coworkers to realize C–N bond alkynylation with acetylenic sulfones under metal-free photoredox catalytic conditions [32]. In 2019, Studer and coworkers utilized alkyl allyl sulfones as alkyl radical precursors to accomplish desulfonylative C (*sp*^3^)–C (*sp*) bond coupling initiated by 2, 2′-azobis (2-methylpropionitrile) (AIBN) [33]. Moreover, some alkanes or functionalized alkanes could be directly converted into internal alkynes with acetylenic sulfones via the diversity of the radical-mediated C–H bond alkynylation process [34,35,36,37,38,39]. Although remarkable achievements have been made in this research area, some of the reactions suffer from several limitations, such as the utilization of expensive catalysts or peroxides and the narrow scope of substrates and prolix procedures for the preparation of the radical precursors. It is highly desirable to establish a practical and efficient platform to afford C (*sp*^3^)–C (*sp*) bond coupling products from readily available substates in the presence of earth-abundant metal catalysts.

Recently, metal (Fe, Co, Mn)-catalyzed hydrofunctionalization of alkenes has been established as an attractive and robust strategy for the construction of structural skeletons via the metal hydride hydrogen atom transfer (MHAT) process [40,41]. The alkenes interact with the metal hydride in situ generated from the metal catalyst with hydrogen sources to form carbon radical species, which were involved in varieties of chemical bonds formation such as C–H [42,43,44], C–C [45,46,47,48,49,50,51,52,53], C–O [54,55,56,57,58], C–S [59,60], C–N [61,62,63,64,65], and C–F [66,67] bond coupling. However, hydrogen atom transfer-triggered the hydrofunctionalization of alkenes, leading to internal alkynes using acetylenic sulfones as alkyne source was less explored. In 2006, Renaud and coworkers disclosed a radical-mediated alkynylation of alkenes to yield internal alkynes under the initiation of di-*tert*-butylhyponitrite, wherein the in situ hydroboration of the alkenes contributed to the excellent anti-Markovnikov selectivity (Scheme 1c) [68]. Herein, we developed an iron-catalyzed strategy to synthesize the internal alkynes with Markovnikov selectivity from readily available alkenes via a MHAT process (Scheme 1d).

## 2. Results

To start our investigation, we probed the reaction employing alkene **1a** (0.3 mmol) and acetylenic sulfone **2a** (0.2 mmol) as model substrates in the presence of Fe(acac)_3_ (30 mol%), PhSiH_3_ (2.0 equiv) in a mixed solvent. As expected, the desired internal alkyne **3a** bearing a quaternary carbon center could be obtained with 81% yield (Table 1, Entry 1). Some other acetylenic sulfones **2a′**–**2a′′′′′** were investigated, and worse results were obtained (Table 1, Entries 2–Entries 6). Additionally, only 62% yield of **3a** was generated if the reaction was operated in EtOH without the addition of (CH_2_OH)_2_, which showed that (CH_2_OH)_2_ played an irreplaceable role contributing to the high efficiency of the transformation (Table 1, Entry 7), because it could suppress the formation of PhSi(OEt)_3_ [46]. Additionally, the yield of desired product **3a** was reduced to 60% with the amount of Fe(acac)_3_ decreasing to 20 mol% (Table 1, Entry 8). After screening of other catalysts including In(acac)_3_, Co(acac)_3_ and FeCl_3_, it was shown that In(acac)_3_ and Co(acac)_3_ were completely ineffective and FeCl_3_ was of modest efficiency, resulting in the alkyne product **3a** with a 45% yield (Table 1, Entries 9–Entries 11). Notably, an apparent decrease in the yield was observed when alkene **1a** (0.2 mmol) and acetylenic sulfone **2a** (0.3 mmol) participated in the reaction (Table 1, Entry 12).

**Figure 1:**
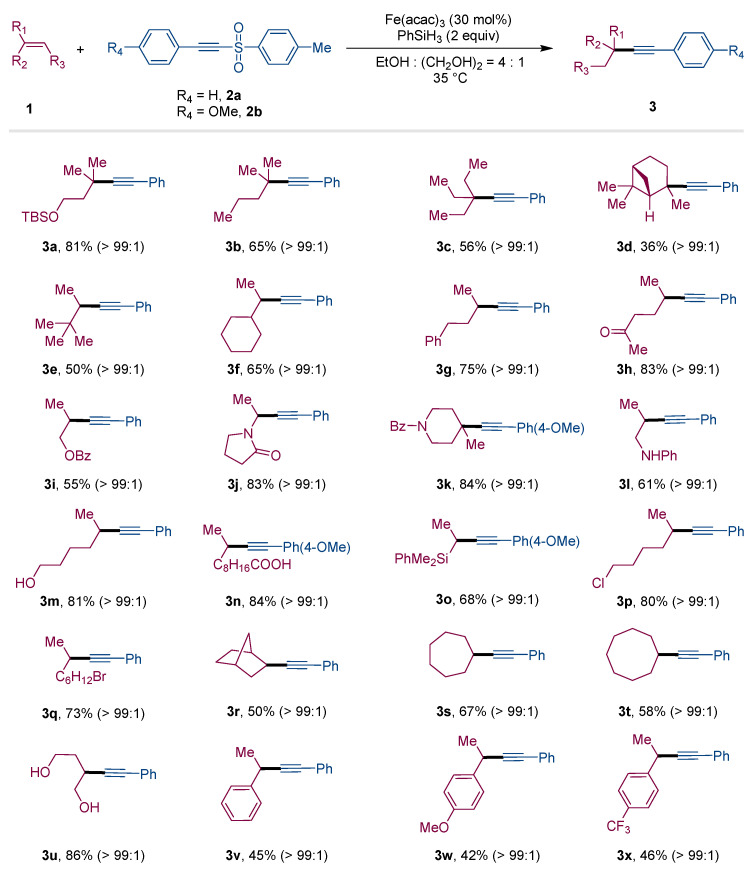
Substrate scope of alkenes ^a^. ^a^ Standard conditions: **1** (0.3 mmol, 1.5 equiv), **2a** or **2b** (0.2 mmol, 1.0 equiv), Fe(acac)_3_ (30 mol%), PhSiH_3_ (0.4 mmol, 2.0 equiv), in EtOH (0.8 mL) and (CH_2_OH)_2_ (0.2 mL) at 35 °C for 12 h, isolated yields.

With the optimal conditions in hand, we then examined the scope of iron-catalyzed SOMOphilic alkynylation, keeping **2a** and **2b** as radical acceptors, which is presented in Figure 1. These simple and mild conditions turned out to be compatible with a wide range of alkenes with exquisite functional group tolerance. *β*-methyl alkenes were investigated as suitable substrates to react with **2a**, affording the substituted alkynes **3a**–**3d** bearing quaternary carbons in modest to good yields. Moreover, alkenes bearing bulky groups also worked well to provide the corresponding alkynes **3e**–**3f** in satisfactory yields. Since this reaction’s conditions were gentle, alkenes bearing a wide of functional groups such as phenyl (**1g**), carbonyl (**1h**), ester (**1i**), amide (**1j**, **1k**), amine (**1l**), hydroxyl (**1m**), carboxyl (**1n**), silicon (**1o**) groups underwent the MHAT-promoted alkynylation in 55% to 84% yields. Notably, although the reactions were operated in mixed alcohols, the alkenes bearing halide atoms performed well, generating desired alkynes **3p**–**3q** in good yields, which could be applied for the further transformations. In addition, the reactions of internal alkenes with alkyne reagent **2a** were operated smoothly, leading to the formation of the alkynylation products **3r**–**3u** in 50% to 86% yields. The styrene derivatives could also be treated as suitable candidates under the optimized conditions to provide the alkynes **3v–3x** in medium yields with excellent selectivity.

**Figure 2:**
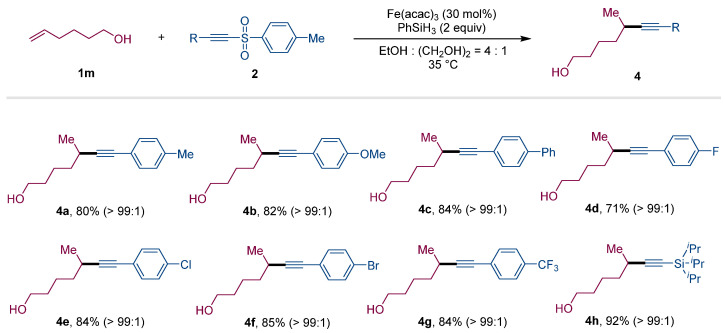
Substrate scope of acetylenic sulfones ^a^. ^a^ Standard conditions: **1m** (0.3 mmol, 1.5 equiv), **2** (0.2 mmol, 1.0 equiv), Fe(acac)_3_ (30 mol%), PhSiH_3_ (0.4 mmol, 2.0 equiv), in EtOH (0.8 mL) and (CH_2_OH)_2_ (0.2 mL) at 35 °C for 12 h, isolated yields.

Encouraged by the results of variable alkenes, we continued to investigate the scope of alkyne sources utilizing alkene **1m** as a radical precursor under the optimal conditions. Diversities of acetylenic sulfones were prepared and participated in the reaction system. As shown in Figure 2, the electron-donating groups, electron-withdrawing groups and halide atoms on the phenyl rings were tolerated. As examples, acetylenic sulfones with methyl, methoxyl, phenyl, fluoro, chloro, bromo and trifluoromethyl groups engage in the reactions, yielding the corresponding products **4a**–**4g** in 71% to 85% yields. Importantly, triisopropylsilacetylene-derived sulfone demonstrated an excellent performance, yielding the product **4h** with a 92% yield, which could be converted into the terminal alkyne under desiliconization conditions.

**Scheme 2:**
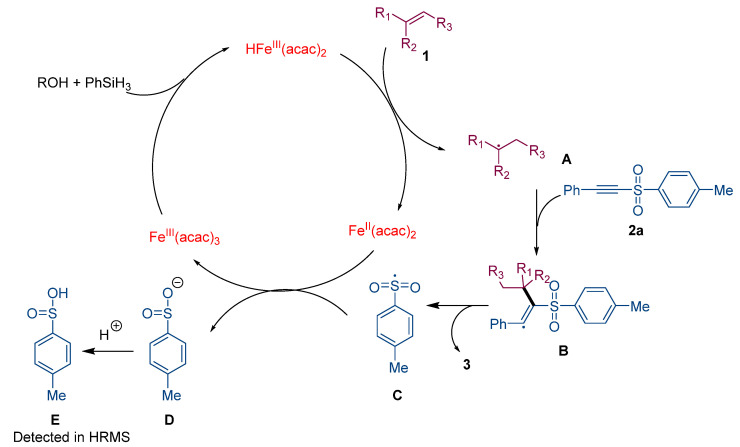
Plausible mechanism.

A tentative mechanism of this SOMOphilic alkynylation of alkenes is depicted in Scheme 2 according to the reported iron-catalyzed hydrofunctionalizations of alkenes via the MHAT process [40] and radical-mediated alkynylation [68]. Initially, Fe^III^(acac)_3_ was converted into the HFe^III^(acac)_2_ species with the interaction of PhSiH_3_ in alcohol. Then, MHAT occurred between HFe^III^(acac)_2_ and non-activated alkenes **1,** acting as a rate-determining step [69], affording carbon-centered radical **A** with excellent Markovnikov selectivity as well as Fe^II^(acac)_2_. Subsequently, anti-Michael addition of **A** onto acetylenic sulfone **2a** generated enyl radical intermediates **B**, followed by the radical-mediated desulfonation to afford the desired alkyne products **3** with a release of sulfonyl radical, achieving the alkynyl functionalization with the realization of sulfonyl radical **C**. Finally, the sulfonyl radical **C** oxidized Fe^II^(acac)_2_ to Fe^III^(acac)_3_ to fulfill the catalytic cycle, generating a sulfinic acid **E** [70], which was detected in HRMS (Figure S1 in Supplementary Materials).

## 3. Discussion

In conclusion, we developed an iron-catalyzed SOMOphilic alkynylation of non-activated alkenes with acetylenic sulfone with Markovnikov selectivity. A wide range of secondary and tertiary alkynes bearing variable functional and sensitive groups could be obtained from readily available and easily prepared starting materials by this efficient and mild MHAT strategy. Additional applications in the synthesis and modification of complex molecules or bioactive compounds are under investigation in our laboratory.

## 4. Materials and Methods

### 4.1. General Information

Unless otherwise noted, all reactions were performed under an argon atmosphere using flame-dried glassware. All new compounds were fully characterized. NMR-spectra were recorded on Bruker ARX-400 MHz or ARX-600 Associated. ^1^H NMR spectra data were reported as δ values in ppm relative to chloroform (δ 7.26) if collected in CDCl_3_. ^13^C NMR spectra data were reported as δ values in ppm relative to chloroform (δ 77.00). ^1^H NMR coupling constants were reported in Hz, and multiplicity was indicated as follows: s (singlet); d (doublet); t (triplet); q (quartet); quint (quintet); m (multiplet); dd (doublet of doublets); ddd (doublet of doublet of doublets); dddd (doublet of doublet of doublet of doublets); dt (doublet of triplets); td (triplet of doublets); ddt (doublet of doublet of triplets); dq (doublet of quartets); app (apparent); and br (broad). Mass spectra were obtained using a Micromass Q-Tof instrument (ESI) and Agilent Technologies 5973N (EI). All reactions were carried out in flame-dried 25 mL Schlenk tubes with Teflon screw caps under an argon atmosphere. Unless otherwise noted, materials obtained from commercial suppliers were used without further purification. Acetylenic sulfones **2** were prepared according to the reported procedures [29,71].

### 4.2. General Procedures of Iron-Catalyzed SOMOphilic Alkynylation

Flame-dried 10 mL Schlenk tube filled with N_2_, acetylenic sulfones **2** (0.2 mmol, 1.0 equiv) and Fe(acac)_3_ (21.2 mg, 0.06 mmol, 30 mol%) were added under N_2_, evacuated and purged with N_2_ three times. Afterwards, PhSiH_3_ (43.2 mg, 0.4 mmol, 2 equiv), non-activted alkenes **1** (33.1 mg, 0.3 mmol, 1.5 equiv) and ethanol (0.8 mL) and ethylene glycol (0.2 mL) were added via syringe. The formed mixture was stirred at 35 °C under N_2_ for 12 h, as monitored by TLC. The solution was then cooled to room temperature, and the solution was diluted with ethyl acetate and transferred to a round bottom flask. The concentrated residue was purified by column chromatography using ethyl acetate / petroleum ether as an eluent to afford the corresponding products and **3** and **4**.

### 4.3. Characterization Data for Products

*tert-Butyl((3,3-dimethyl-5-phenylpent-4-yn-1-yl)oxy)dimethylsilane* (**3a**). Colorless oil (48.9 mg, 81%): ^1^H NMR (500 MHz, CDCl_3_) δ 7.39–7.36 (m, 2H), 7.29–7.25 (m, 3H), 3.90 (t, *J* = 7.4 Hz, 2H), 1.77 (t, *J* = 7.4 Hz, 2H), 1.31 (s, 6H), 0.91 (s, 9H), 0.09 (s, 6H); ^13^C NMR (126 MHz, CDCl_3_) δ 131.5, 128.1, 127.4, 123.9, 96.6, 80.6, 61.0, 45.6, 30.3, 29.8, 26.0, 18.3, −5.2; HRMS *m*/*z* (ESI) calcd for C_19_H_30_N_a_OSi (M + Na)^+^ 325.1958, found 325.1960.

*(3, 3-Dimethylhex-1-yn-1-yl)benzene* (**3b**). Colorless oil (24.2 mg, 65%): ^1^H NMR (400 MHz, CDCl_3_) δ 7.39–7.36 (m, 2H), 7.27–7.24 (m, 3H), 1.55–1.42 (m, 2H), 1.47–1.42 (m, 2H), 1.27 (s, 6H), 0.95 (t, *J* = 7.1 Hz, 3H); ^13^C NMR (101 MHz, CDCl_3_) δ 131.5, 128.1, 127.3, 124.2, 97.6, 80.2, 45.9, 31.7, 29.3, 18.7, 14.6; HRMS *m*/*z* (ESI) calcd for C_14_H_18_N_a_ (M + Na)^+^ 209.1301, found 209.1305.

*(3,3-Diethylpent-1-yn-1-yl)benzene* (**3c**). Colorless oil (22.4 mg, 56%): ^1^H NMR (400 MHz, CDCl_3_) δ 7.41–7.38 (m, 2H), 7.29–7.26 (m, 3H), 1.53 (q, *J* = 7.5 Hz, 6H), 0.97 (t, *J* = 7.5 Hz, 9H); ^13^C NMR (101 MHz, CDCl_3_) δ 131.6, 128.1, 127.2, 124.4, 96.0, 82.23, 39.9, 29.8, 8.8; HRMS *m*/*z* (ESI) calcd for C_15_H_21_ (M + H)^+^ 201.1638, found 201.1639.

*(1S,5R)-2,6,6-Trimethyl-2-(phenylethynyl)bicyclo[3.1.1]heptane* (**3d**). Colorless oil (17.1 mg, 36%): ^1^H NMR (400 MHz, CDCl_3_) δ 7.39–7.36 (m, 2H), 7.26–7.23 (m, 3H), 5.41 (d, *J* = 4.5 Hz, 1H), 2.17 (d, *J* = 18.8 Hz, 1H), 2.03–1.96 (m, 4H), 1.66 (d, *J* = 1.9 Hz, 3H), 1.48–1.42 (m, 2H), 1.30 (s, 3H), 1.26 (s, 3H); ^13^C NMR (101 MHz, CDCl_3_) δ 131.6, 128.1, 127.3, 120.8, 96.6, 81.0, 43.9, 34.7, 31.0, 29.7, 27.5, 27.3, 27.0, 24.8, 23.3; HRMS *m*/*z* (ESI) calcd for C_18_H_23_ (M + H)^+^ 239.1794, found 239.1795.

*(3,4,4-Trimethylpent-1-yn-1-yl)benzene* (**3e**). Colorless oil (18.5 mg, 50%): ^1^H NMR (400 MHz, CDCl_3_) δ 7.41–7.38 (m, 2H), 7.28–7.26 (s, 3H), 2.45 (q, *J* = 7.1 Hz, 1H), 1.20 (d, *J* = 7.1 Hz, 3H), 1.02 (s, 9H); ^13^C NMR (101 MHz, CDCl_3_) δ 131.5, 128.1, 127.3, 124.3, 93.9, 91.8, 37.8, 33.6, 27.3, 15.9; HRMS *m*/*z* (ESI) calcd for C_14_H_18_ N_a_ (M + Na)^+^ 209.1301, found 209.1303.

*(3-Cyclohexylbut-1-yn-1-yl)benzene* (**3f**). Colorless oil (27.7 mg, 65%): ^1^H NMR (400 MHz, CDCl_3_) δ 7.41–7.39 (m, 2H), 7.29–7.26 (m, 3H), 2.53–2.50 (m, 1H), 1.95–1.90 (m, 1H), 1.81–1.74 (m, 3H), 1.69–1.65 (m, 1H), 1.36–1.13 (m, 9H); ^13^C NMR (101 MHz, CDCl_3_) δ 131.5, 128.1, 127.3, 124.2, 93.9, 81.5, 42.9, 32.5, 31.1, 29.5, 26.48, 26.45, 26.4, 18.3; HRMS *m*/*z* (ESI) calcd for C_16_H_21_ (M + H)^+^ 213.1638, found 213.1643.

*(3-Methylpent-1-yne-1,5-diyl)dibenzene* (**3g**). Colorless oil (35.3 mg, 75%): ^1^H NMR (400 MHz, CDCl_3_) δ 7.46– 7.43 (m, 2H), 7.32– 7.29 (m, 5H), 7.26–7.18 (m, 3H), 2.94–2.87 (m, 1H), 2.84–2.76 (m, 1H), 2.71–2.63 (m, 1H), 1.90–1.79 (m, 2H), 1.30 (d, *J* = 6.9 Hz, 3H); ^13^C NMR (101 MHz, CDCl_3_) δ 142.1, 131.6, 128.5, 128.3, 128.2, 127.5, 125.8, 124.0, 94.2, 81.3, 38.7, 33.7, 26.0, 21.1; HRMS *m*/*z* (ESI) calcd for C_18_H_19_ (M + H)^+^ 235.1481, found 235.1482.

*5-Methyl-7-phenylhept-6-yn-2-one* (**3h**). Colorless oil (33.0 mg, 83%): ^1^H NMR (400 MHz, CDCl_3_) δ 7.41–7.36 (m, 2H), 7.30–7.27 (m, 3H), 2.74–2.60 (m, 3H), 2.18 (s, 3H), 1.87–1.83 (m, 1H), 1.75–1.68 (m, 1H), 1.27 (d, *J* = 6.9 Hz, 3H); ^13^C NMR (101 MHz, CDCl_3_) δ 208.7, 131.5, 128.2, 127.7, 123.7, 93.4, 81.5, 41.5, 30.6, 30.1, 26.0, 21.1; HRMS *m*/*z* (ESI) calcd for C_14_H_17_O (M + H)^+^ 201.1274, found 201.1275.

*2-Methyl-4-phenylbut-3-yn-1-yl benzoate* (**3i**). Colorless oil (28.9 mg, 55%): ^1^H NMR (400 MHz, CDCl_3_) δ 8.11–8.09 (m, 2H), 7.59–7.56 (m, 1H), 7.46 (t, *J* = 7.6 Hz, 2H), 7.42–7.39 (m, 2H), 7.28 (q, *J* = 3.2, 2.7 Hz, 3H), 4.46–4.43 (m, 1H), 4.35–4.32 (m, 1H), 3.17 (q, *J* = 6.8 Hz, 1H), 1.39 (d, *J* = 6.9 Hz, 3H); ^13^C NMR (101 MHz, CDCl_3_) δ 166.4, 133.0, 131.6, 130.1, 129.6, 128.3, 128.2, 127.9, 123.3, 90.3, 81.9, 67.9, 26.7, 17.7; HRMS *m*/*z* (ESI) calcd for C_18_H_17_O_2_ (M + H)^+^ 265.1223, found 265.1225.

*1-(4-Phenylbut-3-yn-2-yl)pyrrolidin-2-one* (**3j**). Yellow oil (30.2 mg, 71%): ^1^H NMR (400 MHz, CDCl_3_) δ 7.42–7.39 (m, 2H), 7.31–7.29 (m, 3H), 5.30 (q, *J* = 7.1 Hz, 1H), 3.66–3.60 (m, 1H),3.49–3.43 (m, 1H), 2.44–2.40 (m,2H), 2.08–2.04 (m, 2H), 1.44 (d, *J* = 7.0 Hz, 3H); ^13^C NMR (101 MHz, CDCl_3_) δ 173.9, 131.7, 128.3, 128.2, 122.6, 87.3, 83.5, 42.8, 39.3, 31.2, 19.8, 17.7; HRMS *m*/*z* (ESI) calcd for C_14_H_16_NO (M + H)^+^ 214.1226, found 214.1230.

*(4-((4-Methoxyphenyl)ethynyl)-4-methylpiperidin-1-yl)(phenyl)methanone* (**3k**). Colorless oil (56.1 mg, 84%): ^1^H NMR (400 MHz, CDCl_3_) δ 7.41–7.38 (m, 5H), 7.36–7.32 (m, 2H), 6.84–6.81 (m, 2H), 4.65 (d, *J* = 13.4 Hz, 1H), 3.80 (s, 1H), 3.67–3.65 (m, 1H), 3.45 (t, *J* = 13.1 Hz,1H), 3.24 (d, *J* = 13.3 Hz, 1H), 1.89 (d, *J* = 13.2 Hz,1H), 1.70–1.69 (m, 1H), 1.55 (s, 1H), 1.43–1.39 (m, 1H), 1.35 (s,3H); ^13^C NMR (101 MHz, CDCl_3_) δ 170.3, 159.3, 136.3, 132.9, 129.5, 128.4, 126.9, 115.4, 113.9, 92.0, 83.2, 55.3, 45.5, 39.8, 39.3, 38.4, 32.3, 29.6; HRMS *m*/*z* (ESI) calcd for C_22_H_24_NO_2_ (M + H)^+^ 334.1802, found 334.1803.

*N*-(2-methyl-4-phenylbut-3-yn-1-yl)aniline (**3l**). Colorless oil (28.7 mg, 61%): ^1^H NMR (400 MHz, CDCl_3_) δ 7.44–7.29 (m, 5H), 7.23–7.18 (m, 2H), 6.74–6.67 (m, 3H), 4.05 (br s, 1H), 3.32–3.22 (m, 2H), 3.05–3.00 (m, 1H), 1.34 (d, *J* = 6.9 Hz, 3H); ^13^C NMR (101 MHz, CDCl_3_) δ 147.9, 131.6, 129.3, 128.2, 127.8, 123.4, 117.6, 113.1, 92.0, 82.2, 49.5, 26.8, 18.7; HRMS *m*/*z* (ESI) calcd for C_17_H_18_N (M + H)^+^ 236.1434, found 236.1435.

*5-Methyl-7-phenylhept-6-yn-1-ol* (**3m**). Colorless oil (32.9 mg, 81%): ^1^H NMR (400 MHz, CDCl_3_) δ 7.40–7.38 (m, 2H), 7.29–7.26 (m, 3H), 3.69–3.66 (m, 2H), 2.68–2.63 (m, 1H), 1.63–1.61 (m, 2H), 1.59–1.50 (m, 4H), 1.26 (d, *J* = 6.9 Hz, 3H); ^13^C NMR (101 MHz, CDCl_3_) δ 131.5, 128.1, 127.5, 124.0, 94.5, 80.8, 62.9, 36.7, 32.6, 26.5, 23.62, 21.1; HRMS *m*/*z* (ESI) calcd for C_14_H_19_O (M + H)^+^ 203.1430, found 203.1433.

*12-(4-Methoxyphenyl)-10-methyldodec-11-ynoic acid* (**3n**). Colorless oil (52.4 mg, 84%): ^1^H NMR (400 MHz, CDCl_3_) δ 7.34–7.30 (m, 2H), 6.82–6.79 (m, 2H), 3.79 (s, 3H), 2.65–2.57 (m, 1H), 2.34 (t, *J* = 7.5 Hz, 2H), 1.65–1.59 (m, 2H), 1.53–1.41 (m, 4H), 1.32 (s, 8H), 1.23 (d, *J* = 6.9 Hz, 3H); ^13^C NMR (101 MHz, CDCl_3_) δ 179.6, 158.9, 132.8, 116.3, 113.7, 93.2, 80.3, 55.2, 37.1, 34.0, 29.4, 29.3, 29.2, 29.0, 27.4, 26.5, 24.7, 21.2; HRMS *m*/*z* (ESI) calcd for C_20_H_29_O_3_ (M + H)^+^ 317.2111, found 317.2115.

*(4-(4-Methoxyphenyl)but-3-yn-2-yl)dimethyl(phenyl)silane* (**3o**). Colorless oil (40.1 mg, 68%): ^1^H NMR (400 MHz, CDCl_3_) δ 7.63–7.60 (m, 2H), 7.40–7.36 (m, 3H), 7.30–7.27 (m, 2H), 6.81 (d, *J* = 8.9 Hz, 2H), 3.80 (s, 3H), 2.10 (q, *J* = 7.2 Hz, 1H), 1.22 (d, *J* = 7.3 Hz, 3H), 0.43 (s, 6H); ^13^C NMR (101 MHz, CDCl_3_) δ 158.7, 136.8, 134.1, 132.7, 129.3, 127.7, 116.9, 113.7, 91.9, 80.3, 55.2, 15.0, 13.5, -4.7, -5.4; HRMS *m*/*z* (ESI) calcd for C_19_H_22_NaOSi (M + Na)^+^ 317.1332, found 317.1332.

*(7-Chloro-3-methylhept-1-yn-1-yl)benzene* (**3p**). Colorless oil (35.1 mg, 80%): ^1^H NMR (400 MHz, CDCl_3_) δ 7.40–7.38 (m, 2H), 7.28–7.26 (m, 3H), 3.57 (t, *J* = 6.7 Hz, 2H), 2.7–2.62 (m, 1H), 1.86– 1.79 (m, 2H), 1.72–1.59 (m, 2H), 1.56–1.51 (m, 2H), 1.26 (d, *J* = 6.9 Hz, 3H); ^13^C NMR (101 MHz, CDCl_3_) δ 131.6, 128.2, 127.5, 123.9, 94.2, 81.0, 45.0, 36.2, 32.4, 26.4, 24.8, 21.1; HRMS *m*/*z* (ESI) calcd for C_14_H_18_Cl (M + H)^+^ 221.1092, found 221.1093.

*(9-Bromo-3-methylnon-1-yn-1-yl)benzene* (**3q**). Colorless oil (42.5 mg, 73%): ^1^H NMR (400 MHz, CDCl_3_) δ 7.41–7.39 (m, 2H), 7.29–7.27 (m, 3H), 3.42 (t, *J* = 6.8 Hz, 2H), 2.69–2.60 (m, 1H), 2.91–2.84 (m, 2H), 1.54–1.44 (m, 6H), 1.42–1.34 (m, 2H), 1.26 (d, *J* = 6.9 Hz, 3H); ^13^C NMR (101 MHz, CDCl_3_) δ 131.5, 128.1, 127.4, 124.0, 94.6, 80.8, 36.8, 34.0, 32.8, 28.6, 28.1, 27.2, 26.5, 21.1; HRMS *m*/*z* (ESI) calcd for C_16_H_22_Br (M + H)^+^ 293.0899, found 293.0902.

*(1S,4R)-2-(Phenylethynyl)bicyclo[2.2.1]heptane* (**3r**). Colorless oil (19.4 mg, 50%): ^1^H NMR (400 MHz, CDCl_3_) δ 7.42–7.35 (m, 2H), 7.29–7.23 (m, 3H), 2.47–2.45 (m, 1H), 2.41 (d, *J* = 3.7 Hz, 1H), 2.31 (d, *J* = 4.3 Hz, 1H), 1.72–1.65 (m, 2H), 1.56–1.47 (m, 2H), 1.27–1.16 (m, 4H); ^13^C NMR (101 MHz, CDCl_3_) δ 131.5, 128.1, 127.3, 124.2, 95.7, 80.1, 43.7, 39.4, 36.7, 36.2, 33.6, 28.81, 28.79; HRMS *m*/*z* (ESI) calcd for C_15_H_17_ (M + H)^+^ 197.1325, found 197.1328.

*(Phenylethynyl)cycloheptane* (**3s**). Colorless oil (26.7 mg, 67%): ^1^H NMR (400 MHz, CDCl_3_) δ 7.41–1.38 (m, 2H), 7.30–7.24 (m, 3H), 2.84–2.78 (m, 1H), 1.95–1.87 (m, 2H), 1.81–1.72 (m, 4H), 1.64–1.48 (m, 6H); ^13^C NMR (101 MHz, CDCl_3_) δ 131.5, 128.1, 127.3, 124.2, 95.2, 80.8, 34.7, 31.7, 27.9, 25.6; HRMS *m*/*z* (ESI) calcd for C_15_H_18_Na (M + Na)^+^ 221.1301, found 221.1302.

*(Phenylethynyl)cyclooctane* (**3t**). Colorless oil (24.5 mg, 58%): ^1^H NMR (400 MHz, CDCl_3_) δ 7.40–7.37 (m, 2H), 7.27–7.26 (m, 3H), 2.82–2.76 (m, 1H), 1.98–1.91 (m, 2H), 1.81– 1.72 (m, 4H), 1.56–1.53 (m, 8H); ^13^C NMR (101 MHz, CDCl_3_) δ 131.5, 128.1, 127.3, 124.2, 31.6, 30.7, 29.7, 27.4, 25.4, 24.5; HRMS *m*/*z* (ESI) calcd for C_16_H_21_ (M + H)^+^ 213.1638, found 213.1641.

*2-(Phenylethynyl)butane-1,4-diol* (**3u**). Colorless oil (32.7 mg, 86%): ^1^H NMR (400 MHz, CDCl_3_) δ 7.42–7.40 (m, 2H), 7.30–7.28 (m, 3H), 3.95–3.90 (m, 1H), 3.87–3.84 (m, 1H), 3.77–3.72 (m, 2H), 3.03–3.00 (m, 1H), 2.47 (br s, 2H), 1.94–1.84 (m, 2H); ^13^C NMR (101 MHz, CDCl_3_) δ 131.7, 128.3, 128.1, 123.0, 89.1, 83.8, 65.4, 60.6, 34.5, 33.2; HRMS *m*/*z* (ESI) calcd for C_12_H_15_O_2_ (M + H)^+^ 191.1067, found 191.1068.

*But-1-yne-1,3-diyldibenzene* (**3v**). Colorless oil (18.5 mg, 45%): ^1^H NMR (400 MHz, CDCl_3_) δ 7.47–7.44 (m, 5H), 7.36–7.33 (m, 2H), 7.30–7.28 (m, 3H), 3.99 (q, *J* = 7.2 Hz, 1H), 1.59 (d, *J* = 7.1 Hz, 3H); ^13^C NMR (101 MHz, CDCl_3_) δ 131.6, 130.2, 128.5, 128.2, 127.7, 126.9, 126.6, 123.7, 92.6, 32.5, 24.5; HRMS *m*/*z* (ESI) calcd for C_16_H_15_ (M + H)^+^ 207.1168, found 207.1171.

*1-Methoxy-4-(4-phenylbut-3-yn-2-yl)benzene* (**3w**). Colorless oil (19.8 mg, 42%): ^1^H NMR (400 MHz, CDCl_3_) δ 7.47–7.43 (m, 2H), 7.38–7.36 (m, 2H), 7.32–7.28 (m, 3H), 6.91–6.87 (m, 2H), 3.95 (q, *J* = 7.1 Hz, 1H), 3.81 (s, 3H), 1.56 (d, *J* = 7.1 Hz, 3H); ^13^C NMR (101 MHz, CDCl_3_) δ 158.3, 135.5, 131.6, 128.2, 127.9, 127.7, 123.8, 113.9, 92.9, 82.2, 55.3, 31.6, 24.6; HRMS *m*/*z* (ESI) calcd for C_17_H_17_O (M + H)^+^ 237.1274, found 237.1276.

*1-(4-Phenylbut-3-yn-2-yl)-4-(trifluoromethyl)benzene* (**3x**). Colorless oil (25.3 mg, 46%): ^1^H NMR (400 MHz, CDCl_3_) δ 7.62–7.56 (m, 4H), 7.46–7.43 (m, 2H), 7.33–7.29 (m, 2H), 4.04 (q, *J* = 7.1 Hz, 1H), 1.60 (d, *J* = 7.2 Hz, 3H); ^13^C NMR (101 MHz, CDCl_3_) δ 147.3, 131.6, 128.3, 128.0, 127.3, 125.5 (q, *J* = 3.9 Hz), 123.3, 91.4, 83.1, 32.4, 24.3; ^19^F NMR (376 MHz, Chloroform-*d*) δ -62.4; HRMS *m*/*z* (ESI) calcd for C_17_H_14_F_3_ (M + H)^+^ 275.1042, found 275.1045.

*5-Methyl-7-(p-tolyl)hept-6-yn-1-ol* (**4a**). Colorless oil (34.6 mg, 80%): ^1^H NMR (400 MHz, CDCl_3_) δ 7.29–7.27 (m, 2H), 7.10–7.07 (m, 2H), 3.69–3.65 (m, 2H), 2.69–2.60 (m, 1H), 2.33 (s, 3H), 1.66–1.52 (m, 6H), 1.25 (d, *J* = 6.9 Hz, 3H); ^13^C NMR (101 MHz, CDCl_3_) δ 137.4, 131.4, 128.9, 120.9, 93.6, 80.9, 62.9, 36.78, 32.6, 26.5, 23.6, 21.4, 21.1; HRMS *m*/*z* (ESI) calcd for C_15_H_21_O (M + H)^+^ 217.1587, found 217.1589.

*7-(4-Methoxyphenyl)-5-methylhept-6-yn-1-ol* (**4b**). Colorless oil (38.2 mg, 82%): ^1^H NMR (400 MHz, CDCl_3_) δ 7.33–7.31 (m, 2H), 6.82–6.79 (m, 2H), 3.79 (s, 3H), 3.69–3.65 (m, 2H), 2.68–2.59 (m, 1H), 1.63–1.51 (m, 6H), 1.24 (d, *J* = 6.9 Hz, 3H); ^13^C NMR (101 MHz, CDCl_3_) δ 159.0, 132.9, 116.1, 113.8, 92.8, 80.5, 62.9, 55.2, 36.8, 32.6, 26.5, 23.6, 21.2; HRMS *m*/*z* (ESI) calcd for C_15_H_20_NaO_2_ (M + Na)^+^ 255.1356, found 255.1359.

*7-([1,1′-Biphenyl]-4-yl)-5-methylhept-6-yn-1-ol* (**4c**). Colorless oil (47.0 mg, 84%): ^1^H NMR (400 MHz, CDCl_3_) δ 7.60–7.57 (m, 2H), 7.54–7.51 (m, 2H), 7.48–7.42 (m, 2H), 7.37–7.34 (m, 1H), 3.71–3.67 (m, 2H), 2.73–2.65 (m, 1H), 1.66–1.56 (m, 6H), 1.28 (d, *J* = 6.9 Hz, 3H); ^13^C NMR (101 MHz, CDCl_3_) δ 140.5, 140.2, 131.9, 128.8, 127.4, 126.9, 126.8, 122.9, 95.2, 80.7, 62.9, 36.7, 32.6, 26.6, 23.7, 21.1; HRMS *m*/*z* (ESI) calcd for C_20_H_23_ (M + H)^+^ 279.1743, found 279.1745.

*7-(4-Fluorophenyl)-5-methylhept-6-yn-1-ol* (**4d**). Colorless oil (31.5 mg, 71%): ^1^H NMR (400 MHz, CDCl_3_) δ 7.37–7.34 (m, 2H), 6.98–6.95 (m, 2H), 3.67 (t, *J* = 6.0 Hz, 2H), 2.66–2.61 (m, 1H), 1.65–1.48 (m, 6H), 1.25 (d, *J* = 6.9 Hz, 3H); ^13^C NMR (101 MHz, CDCl_3_) δ 162.0 (d, *J* = 248.1 Hz), 133.3 (d, *J* = 8.1 Hz), 115.3 (d, *J* = 22.4 Hz), 94.1, 79.8, 62.9, 36.7, 32.5, 26.5, 23.6, 21.0; ^19^F NMR (376 MHz, CDCl_3_) δ -112.6; HRMS *m*/*z* (ESI) calcd for C_14_H_18_FO (M + H)^+^ 221.1336, found 221.1339.

*7-(4-Chlorophenyl)-5-methylhept-6-yn-1-ol* (**4e**). Colorless oil (39.9 mg, 84%): ^1^H NMR (400 MHz, CDCl_3_) δ 7.32–7.29 (m, 2H), 7.26–7.23 (m, 2H), 3.68–3.65 (m, 2H), 2.62–2.67 (m, 1H), 1.63–1.50 (m, 6H), 1.25 (d, *J* = 6.9 Hz, 3H); ^13^C NMR (101 MHz, CDCl_3_) δ 133.4, 132.8, 128.4, 122.5, 95.5, 79.8, 62.9, 36.6, 32.5, 26.5, 23.6, 21.0; HRMS *m*/*z* (ESI) calcd for C_14_H_18_ClO (M + H)^+^ 237.1041, found 237.1045.

*7-(4-Bromophenyl)-5-methylhept-6-yn-1-ol* (**4f**). Colorless oil (47.8 mg, 85%): ^1^H NMR (400 MHz, CDCl_3_) δ 7.41–7.39 (m, 2H), 7.26–7.23 (m, 2H), 3.68–3.65 (m, 2H), 2.68–2.59 (m, 1H), 1.65–1.52 (m, 6H), 1.24 (d, *J* = 6.9 Hz, 3H); ^13^C NMR (101 MHz, CDCl_3_) δ 133.0, 131.3, 122.9, 121.5, 95.7, 79.9, 62.9, 36.6, 32.5, 26.6, 23.6, 20.9; HRMS *m*/*z* (ESI) calcd for C_14_H_18_BrO (M + H)^+^ 281.0536, found 281.0538.

*Methyl-7-(4-(trifluoromethyl)phenyl)hept-6-yn-1-ol* (**4g**). Colorless oil (45.4 mg, 84%): ^1^H NMR (400 MHz, CDCl_3_) δ 7.29–7.27 (m, 2H), 7.10–7.07 (m, 2H), 3.69–3.65 (m, 2H), 2.69–2.60 (m, 1H), 2.33 (s, 3H), 1.66–1.52 (m,6H), 1.25 (d, *J* = 6.9 Hz, 3H); ^13^C NMR (101 MHz, CDCl_3_) δ 139.4, 131.8, 129.2 (q, *J* = 32.6 Hz), 125.1 (q, *J* = 3.7 Hz), 124.0 (q, *J* = 273.5 Hz), 97.3, 79.8, 62.9, 36.5, 32.5, 26.6, 23.6, 20.9; ^19^F NMR (376 MHz, CDCl_3_) δ -62.7; HRMS *m*/*z* (ESI) calcd for C_15_H_17_F_3_NaO (M + Na)^+^ 293.1124, found 293.1125.

*5-Methyl-7-(triisopropylsilyl)hept-6-yn-1-ol* (**4h**). Colorless oil (51.9 mg, 92%): ^1^H NMR (400 MHz, CDCl_3_) δ 3.64 (t, *J* = 6.3 Hz, 2H), 2.51–2.43 (m, 1H), 1.65–1.42 (m, 6H), 1.17 (d, *J* = 6.9 Hz, 3H), 1.09–1.00 (m, 21H); ^13^C NMR (101 MHz, CDCl_3_) δ 113.7, 79.8, 63.0, 36.7, 32.5, 26.9, 23.5, 21.3, 18.6, 11.3; HRMS *m*/*z* (ESI) calcd for C_17_H_35_OSi (M + H)^+^ 283.2452, found 283.2455.

## Figures and Tables

**Table 1:**
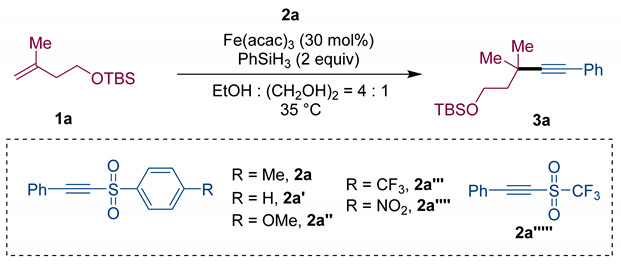
Optimization of SOMOphilic alkynylation of alkenes ^a^.

Entry	Variation from the “Standard Conditions”	Yield (%) ^b^
Entry 1	none	85 (81) ^c^
Entry 2	**2a′** instead of **2a**	80 (72) ^c^
Entry 3	**2a″** instead of **2a**	71
Entry 4	**2a‴** instead of **2a**	49
Entry 5	**2a⁗** instead of **2a**	ND
Entry 6	**2a′′′′′** instead of **2a**	ND
Entry 7	Only EtOH instead of EtOH and (CH_2_OH)_2_	62
Entry 8	Fe(acac)_3_ (20 mol%) instead of Fe(acac)_3_ (30 mol%)	60
Entry 9	In(acac)_3_ instead of Fe(acac)_3_	ND
Entry 10	Co(acac)_3_ instead of Fe(acac)_3_	ND
Entry 11	FeCl_3_ instead of Fe(acac)_3_	45
Entry 12	**1a** (0.2 mmol), **2a** (0.3 mmol)	65

^a^ Standard conditions: **1a** (0.3 mmol, 1.5 equiv), **2a** (0.2 mmol, 1.0 equiv), Fe(acac)_3_ (30 mol%), PhSiH_3_ (0.4 mmol, 2.0 equiv), in EtOH (0.8 mL) and (CH_2_OH)_2_ (0.2 mL) at 35 °C for 12 h. ^b^ Determined by GC-MS using dodecane as the internal standard. ^c^ Isolated yield in parentheses.

## Data Availability

The data presented in this study are available on request from the corresponding authors.

## Supplementary Materials

The following are available online. Figure S1: HRMS spectra of sulfinic acid **E**; 1H NMR, ^13^C NMR and ^19^F NMR spectra of starting materials and products.

## Sample Availability

Samples of the compounds are not available from the authors.
