# Supplementary material for: SOMOphilic Alkynylation of Unreactive Alkenes Enabled by Iron-Catalyzed Hydrogen Atom Transfer
Source: Molecules. 2021 Dec 22;27(1):33. doi: 10.3390/molecules27010033 (PMC8746543; doi:10.3390/molecules27010033)
Supplement: Supplementary file 1 [file molecules-27-00033-s001.zip › molecules-1515263-supplementary.pdf]

SOMOphilic Alkynylation of Unreactive Alkenes Enabled by Iron-Catalysed Hydrogen Atom Transfer

Supporting information

Binlin Zhao [1,*], Tianxiang Zhu [1], Mengtao Ma [1], and Zhuangzhi Shi [2,*]

[1]Department of Chemistry and Material Science, College of Science, Nanjing Forestry University, Nanjing 210037, China

[2]State Key Laboratory of Coordination Chemistry, Chemistry and Biomedicine Innovation Center (ChemBIC), School of Chemistry and Chemical Engineering, Nanjing University, Nanjing 210093, China

Mechanistic Study

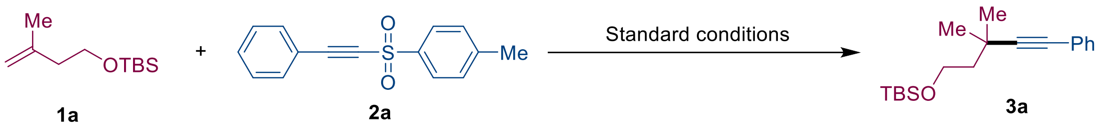

We conducted the reaction of **1a** with **2a** under standard conditions, when the reaction was finished, the resulting mixture was analyzed by HRMS, and the corresponding sulfinic acid **E** was detected (Figure S1).

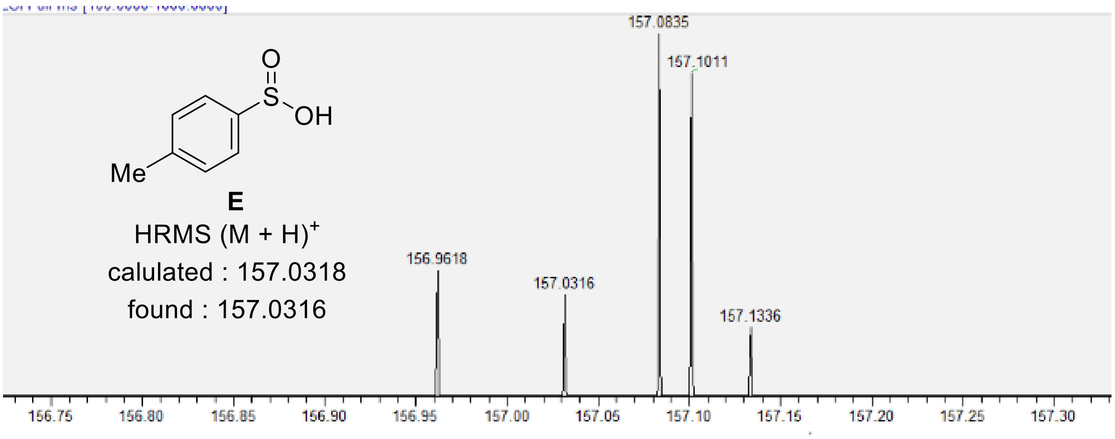

Figure S1: HRMS spectra of sulfinic acid E.

Copies of NMR Spectra

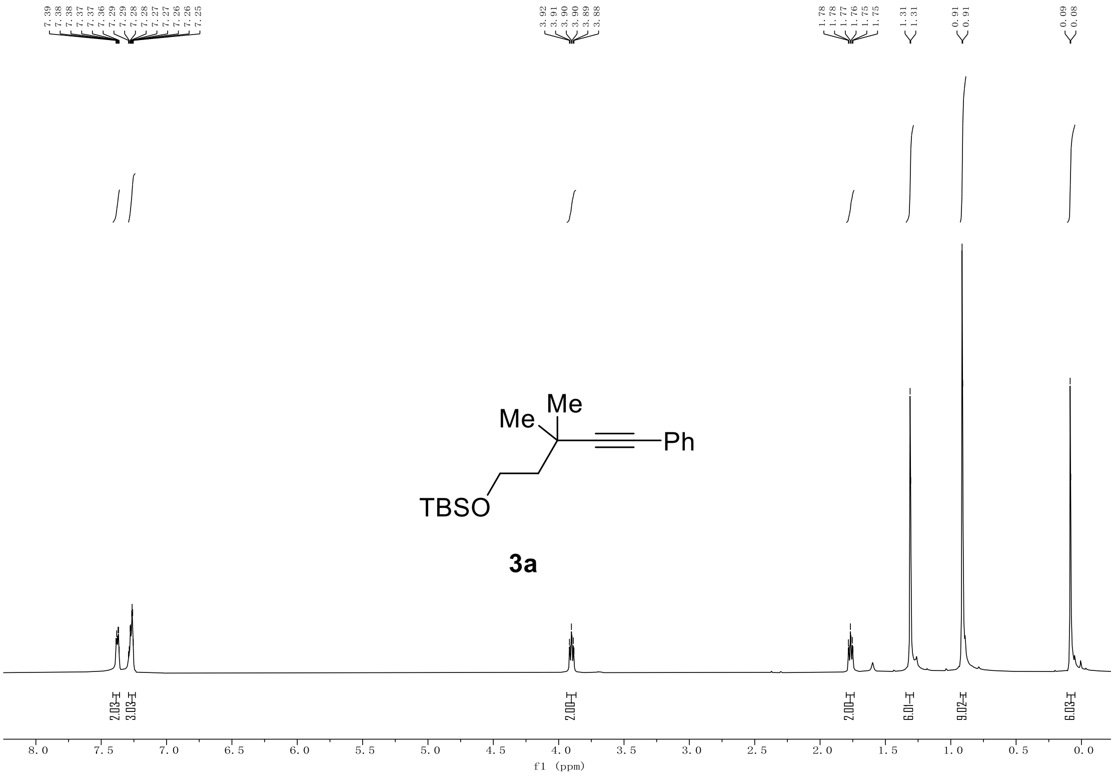

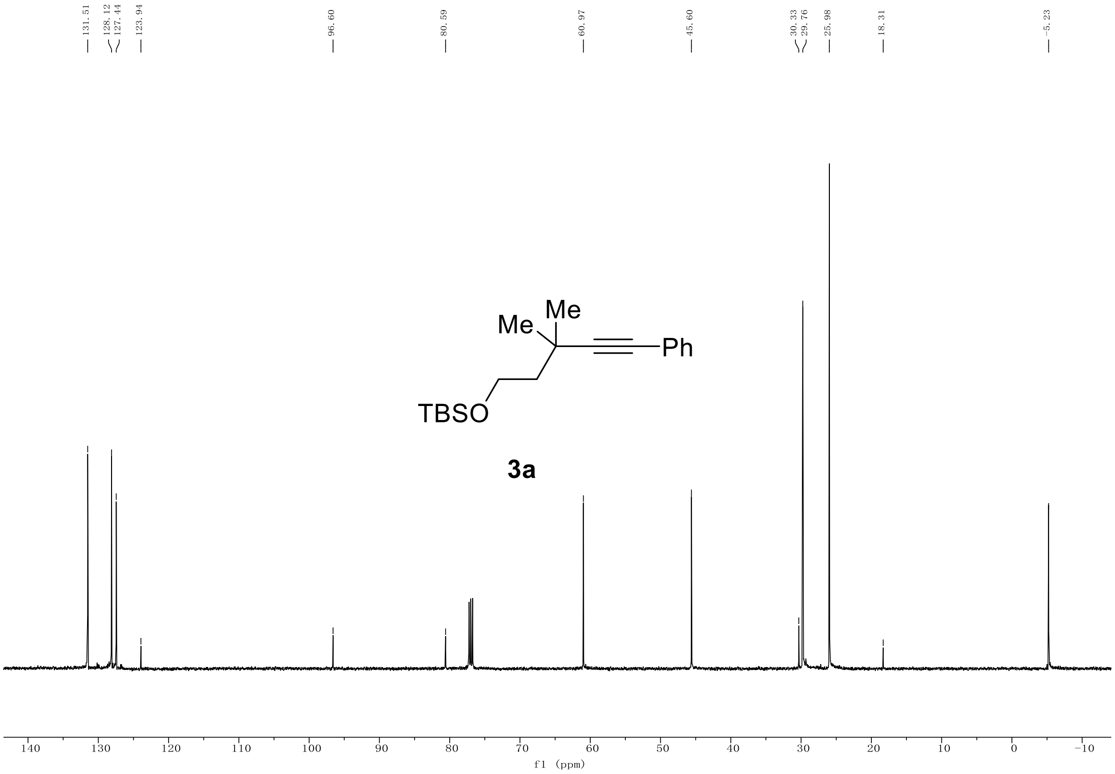

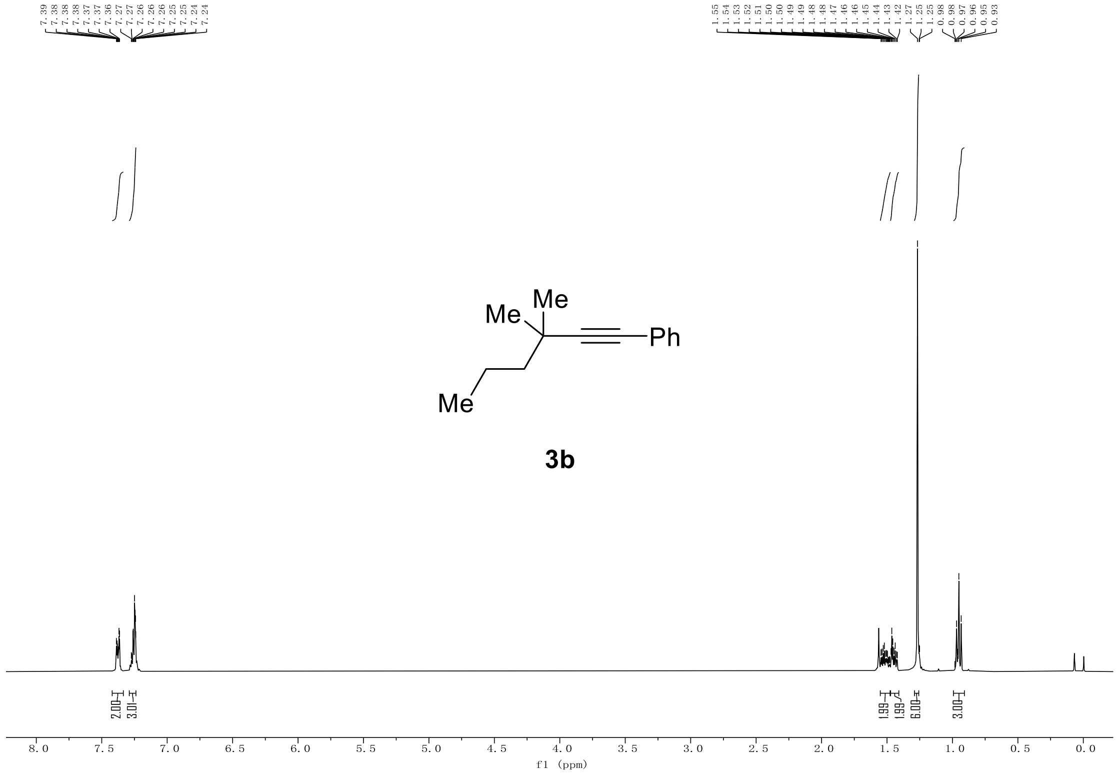
7.39 7.38 7.38 7.38 7.37 7.37 7.36 7.27 7.27 7.26 7.26 7.26 7.25 7.25 7.24 7.24
1.55 1.54 1.53 1.52 1.51 1.50 1.50 1.49 1.49 1.48 1.48 1.47 1.46 1.46 1.45 1.44 1.43 1.42 1.27 1.25 1.25 0.98 0.98 0.97 0.96 0.95 0.93
Me
Me
Ph
Me
3b
2.00
3.01
1.99
1.99
6.00
3.00
8.0 7.5 7.0 6.5 6.0 5.5 5.0 4.5 4.0 3.5 3.0 2.5 2.0 1.5 1.0 0.5 0.0
f1 (ppm)

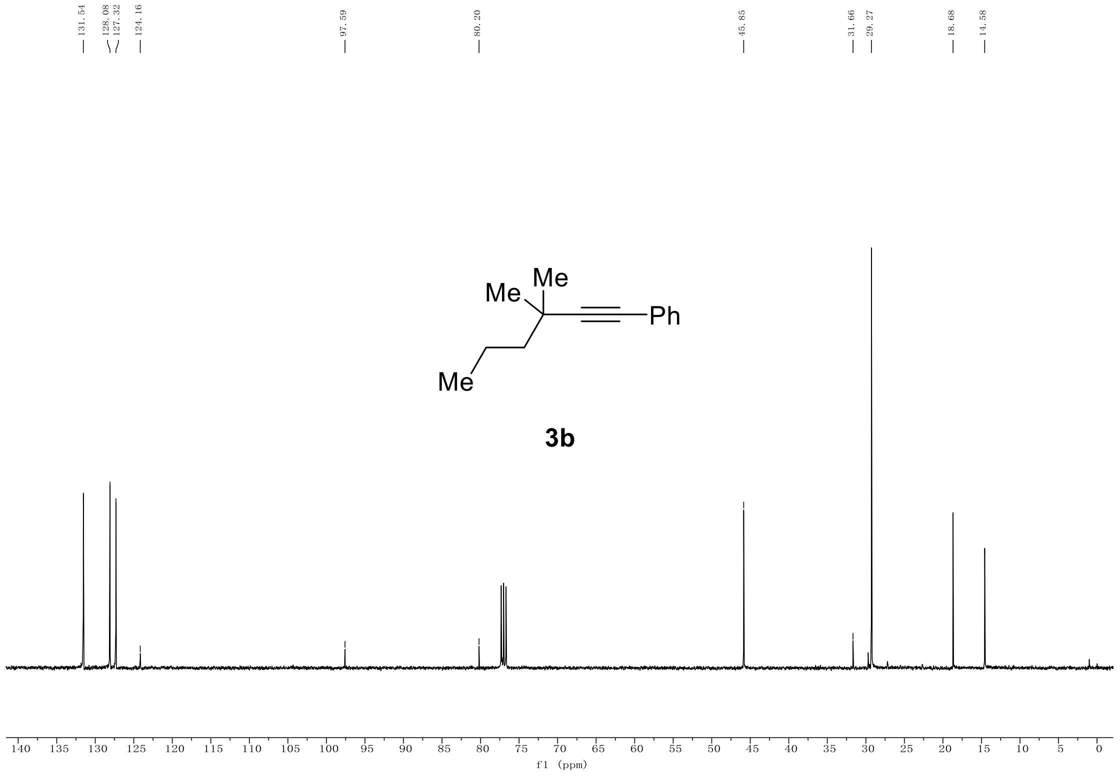
131.54
128.08
127.32
124.16
97.59
80.20
45.85
31.66
29.27
18.68
14.58
Me
Me
Ph
Me
3b
140 135 130 125 120 115 110 105 100 95 90 85 80 75 70 65 60 55 50 45 40 35 30 25 20 15 10 5 0
f1 (ppm)

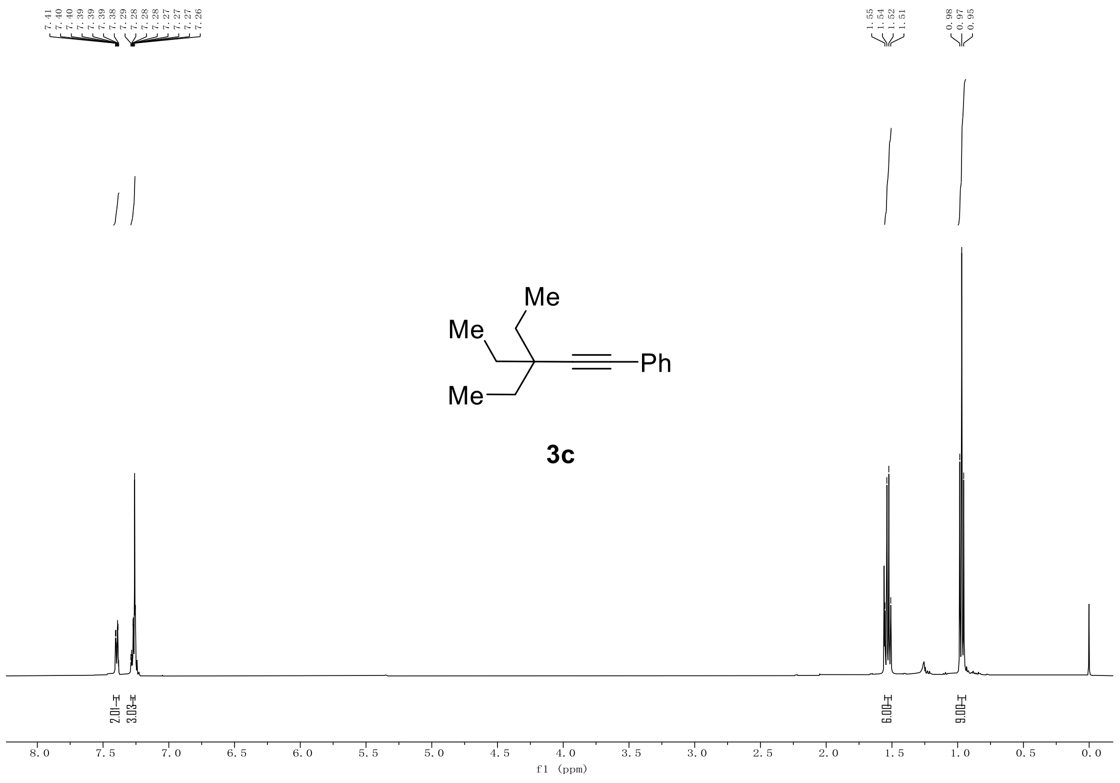

7.41 7.40 7.40 7.39 7.39 7.39 7.38 7.29 7.28 7.28 7.28 7.28 7.27 7.27 7.27 7.26
1.55 1.54 1.52 1.51
0.98 0.97 0.95
Me
Me
Ph
Me
3c
2.01
3.03
6.00
9.00
8.0 7.5 7.0 6.5 6.0 5.5 5.0 4.5 4.0 3.5 3.0 2.5 2.0 1.5 1.0 0.5 0.0
f1 (ppm)

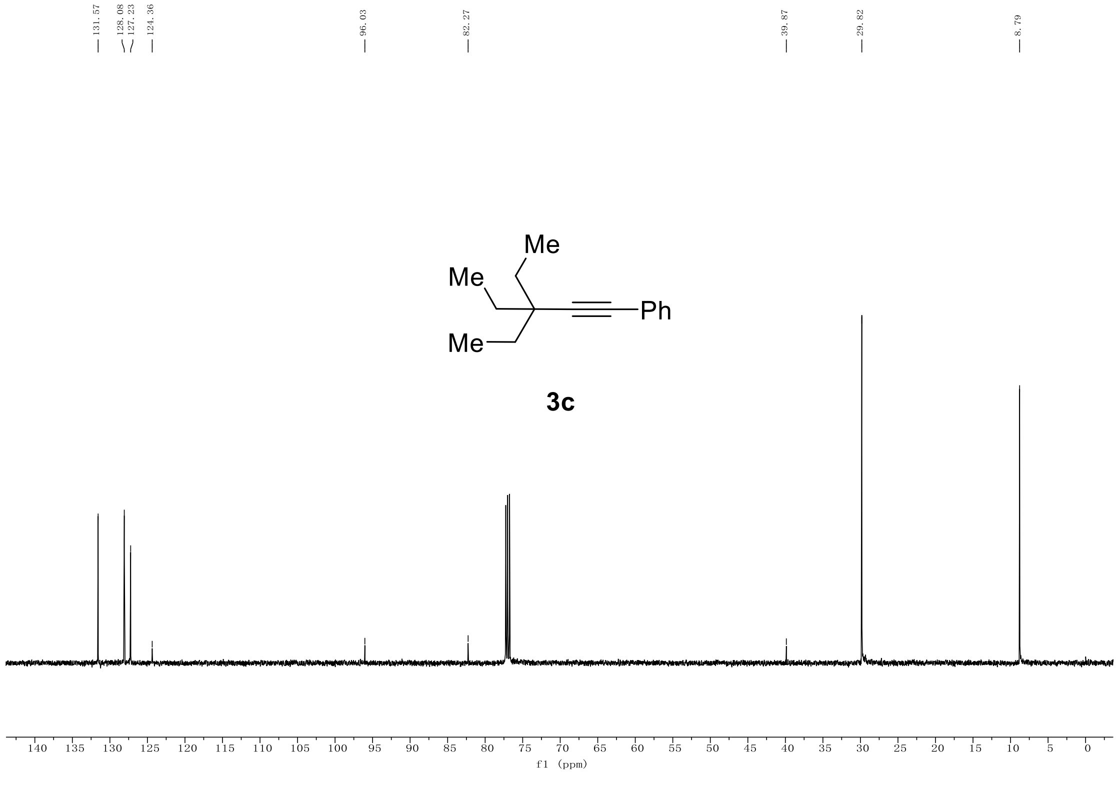

131.57 128.08 127.23 124.36
96.03
82.27
39.87
29.82
8.79
Me
Me
Ph
Me
3c
140 135 130 125 120 115 110 105 100 95 90 85 80 75 70 65 60 55 50 45 40 35 30 25 20 15 10 5 0
f1 (ppm)

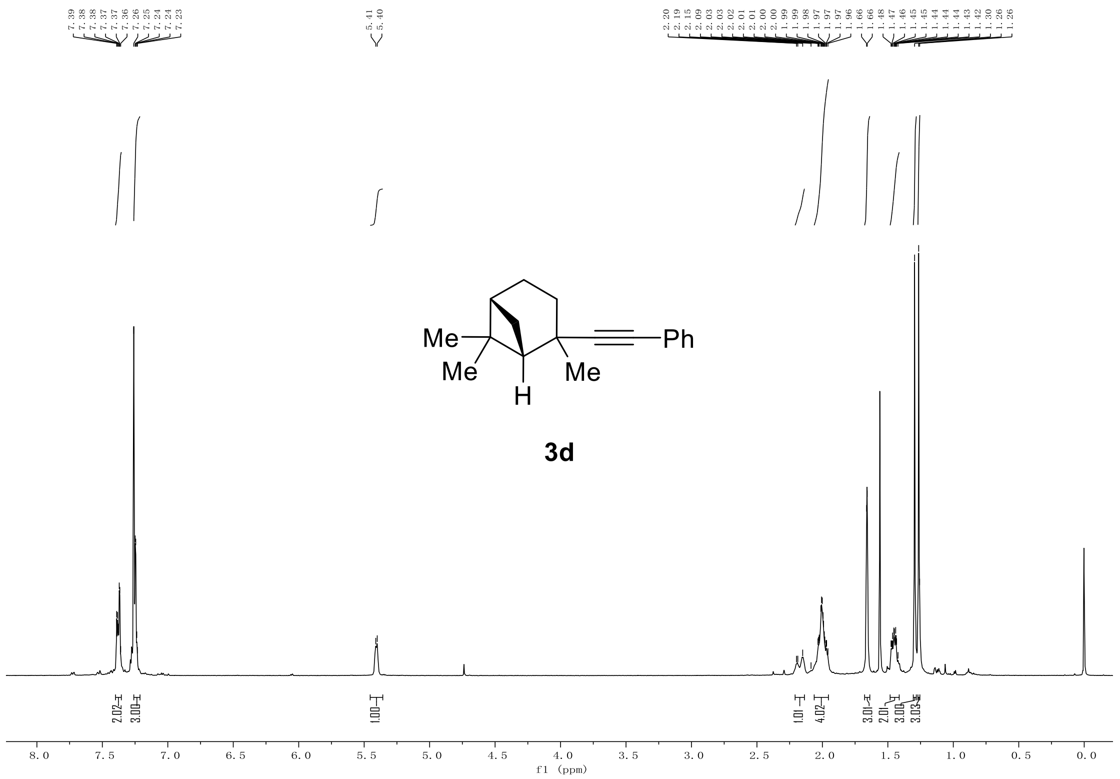
7.39
7.38
7.38
7.37
7.37
7.36
7.26
7.25
7.24
7.24
7.24
7.23
5.41
5.40
2.20
2.19
2.15
2.09
2.03
2.03
2.02
2.01
2.01
2.00
2.00
1.99
1.99
1.98
1.97
1.97
1.97
1.96
1.66
1.56
1.48
1.47
1.46
1.45
1.45
1.44
1.44
1.44
1.43
1.42
1.30
1.28
Me
Me
Me
H
Ph
3d
2.02
3.00
1.00
1.01
4.02
3.01
2.01
3.00
3.03
f1 (ppm)

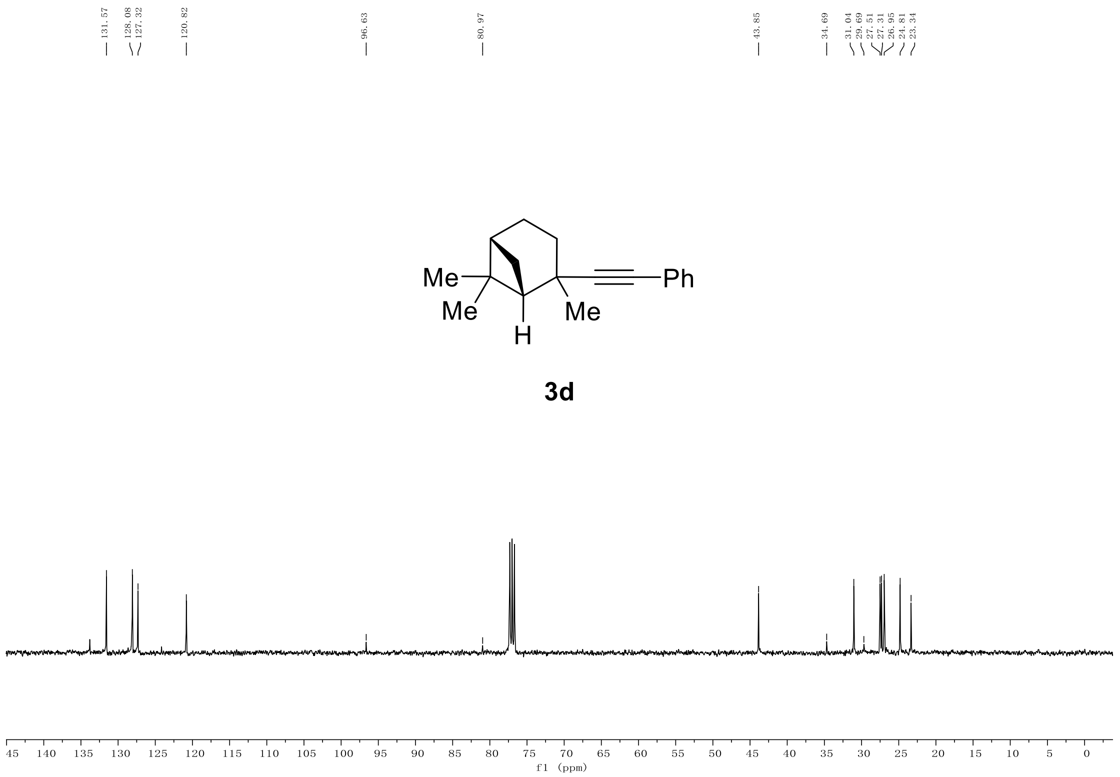
131.57
128.08
127.32
120.82
96.63
80.97
43.85
34.69
31.04
29.69
27.51
27.31
26.95
24.81
23.34
Me
Me
Me
H
Ph
3d
f1 (ppm)

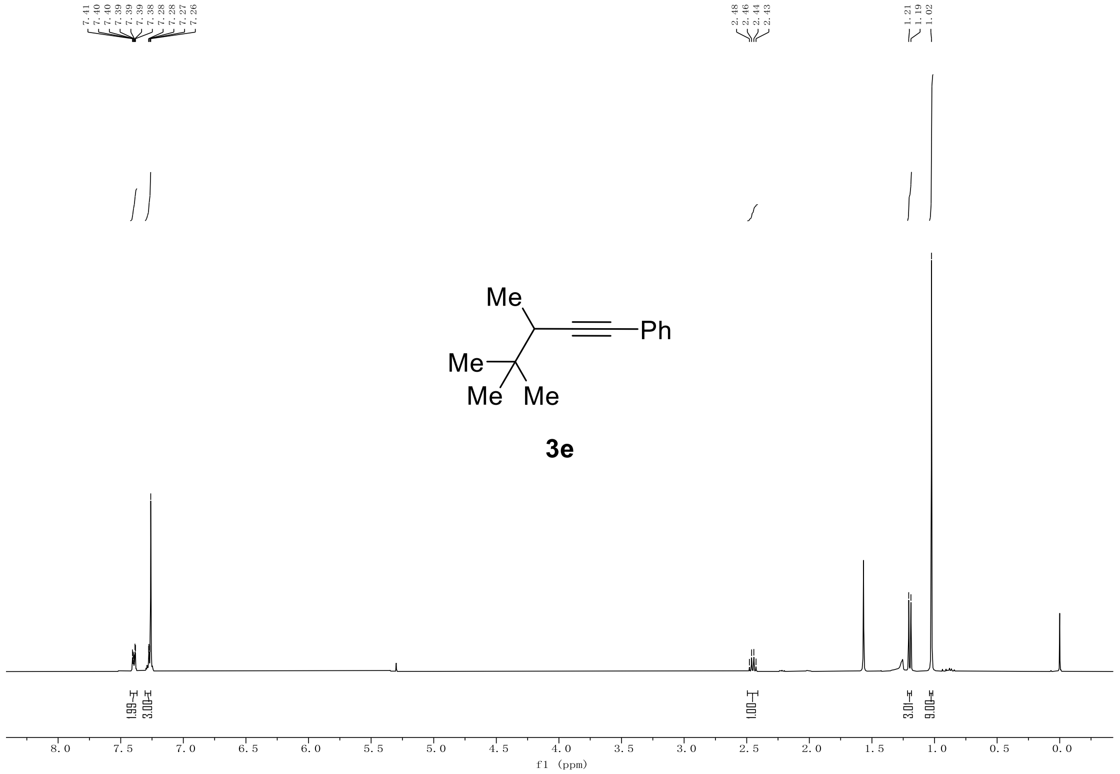
7.41 7.40 7.40 7.39 7.39 7.39 7.38 7.28 7.28 7.27 7.26
2.48 2.46 2.44 2.43
1.21 1.19 1.02
Me
Ph
Me
Me Me
3e
1.99
3.00
1.00
3.01
9.00
8.0 7.5 7.0 6.5 6.0 5.5 5.0 4.5 4.0 3.5 3.0 2.5 2.0 1.5 1.0 0.5 0.0
f1 (ppm)

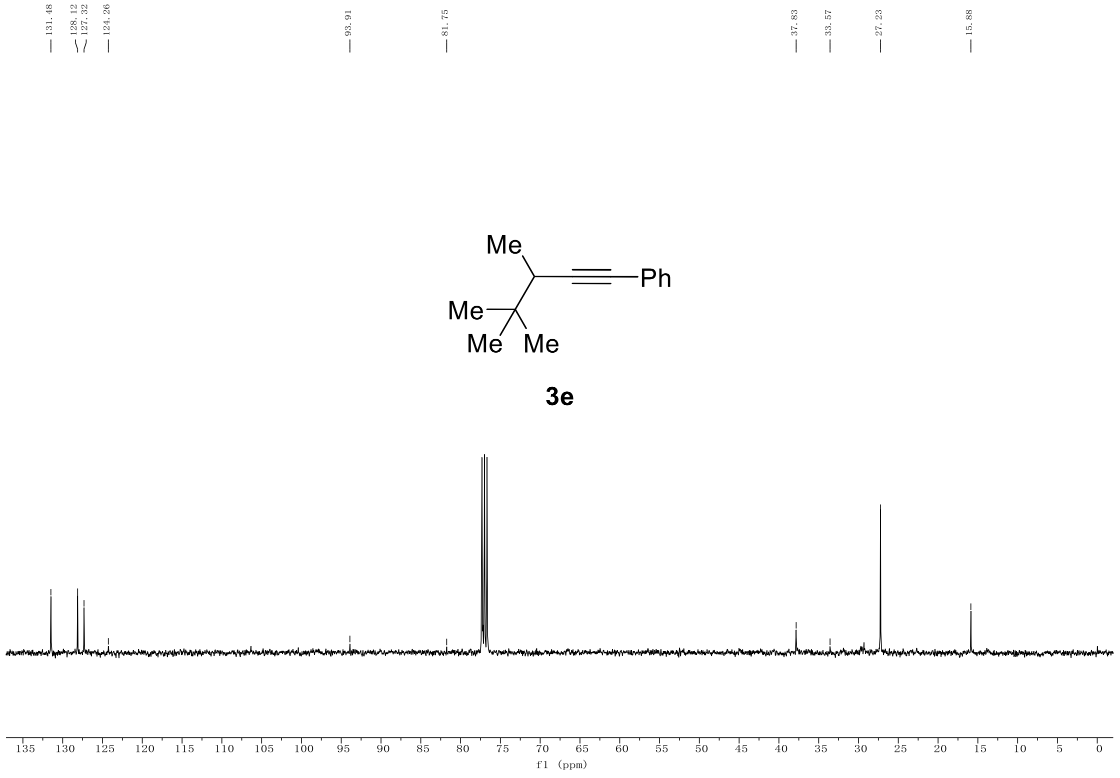
131.48 128.12 127.32 124.26
93.91
81.75
37.83 33.57 27.23 15.88
Me
Ph
Me
Me Me
3e
135 130 125 120 115 110 105 100 95 90 85 80 75 70 65 60 55 50 45 40 35 30 25 20 15 10 5 0
f1 (ppm)

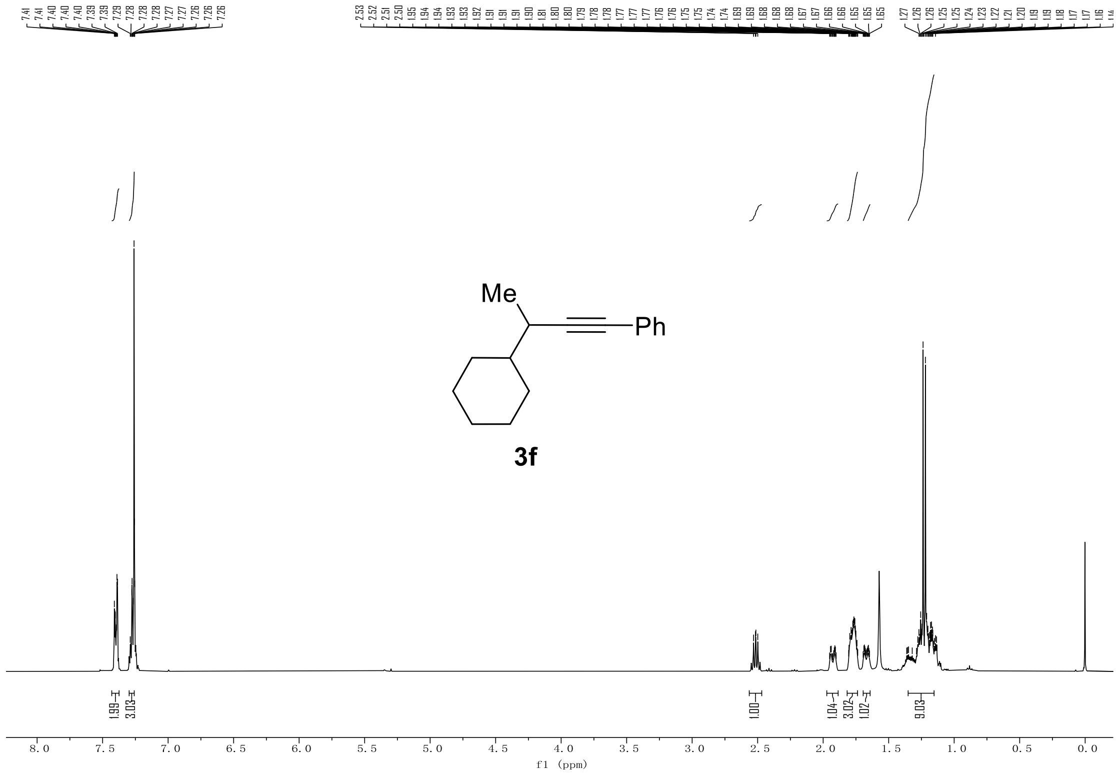
Me
Ph
3f
1.99
3.03
1.00
1.04
3.02
1.02
9.03
8.0 7.5 7.0 6.5 6.0 5.5 5.0 4.5 4.0 3.5 3.0 2.5 2.0 1.5 1.0 0.5 0.0
f1 (ppm)

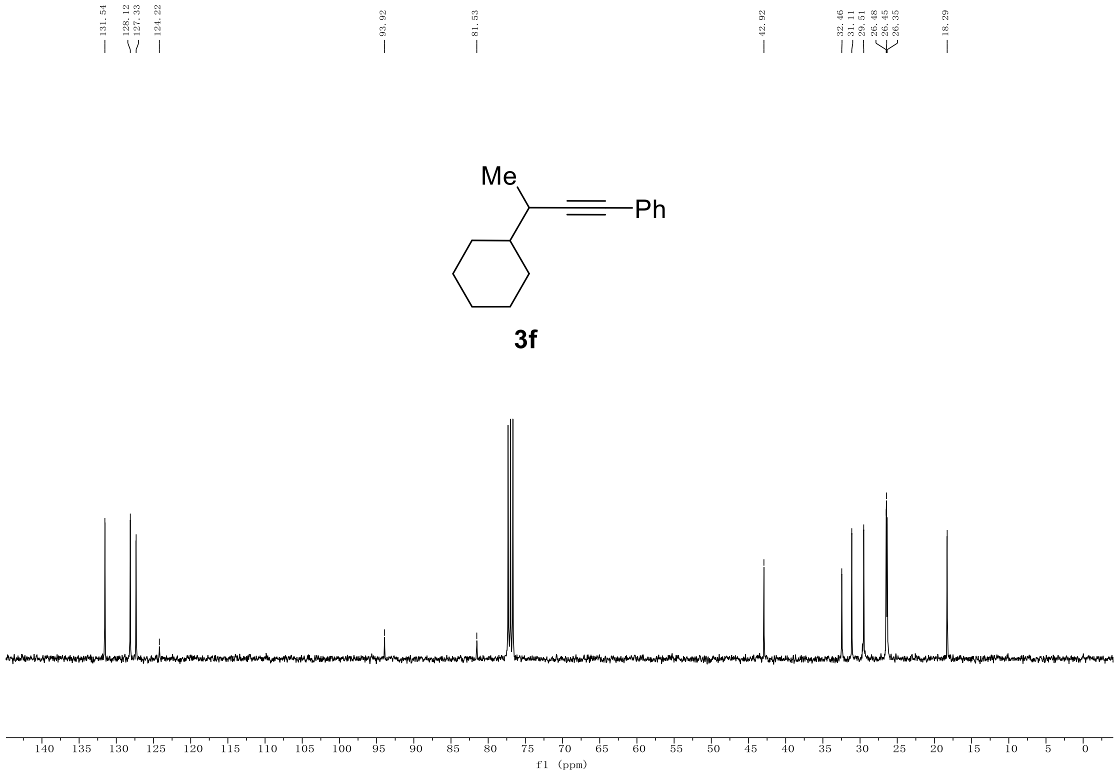
131.54
128.12
127.33
124.22
93.92
81.53
42.92
32.46
31.11
29.51
26.48
26.45
26.35
18.29
Me
Ph
3f
140 135 130 125 120 115 110 105 100 95 90 85 80 75 70 65 60 55 50 45 40 35 30 25 20 15 10 5 0
f1 (ppm)

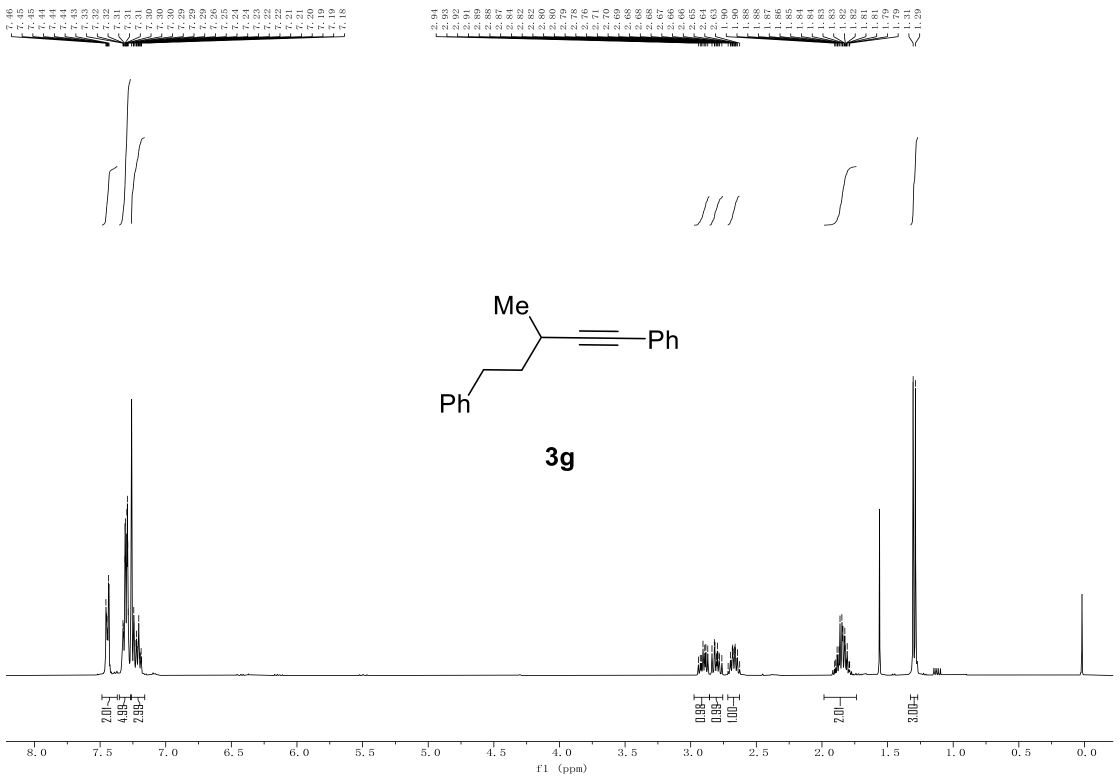
Me
Ph
Ph
3g
f1 (ppm)

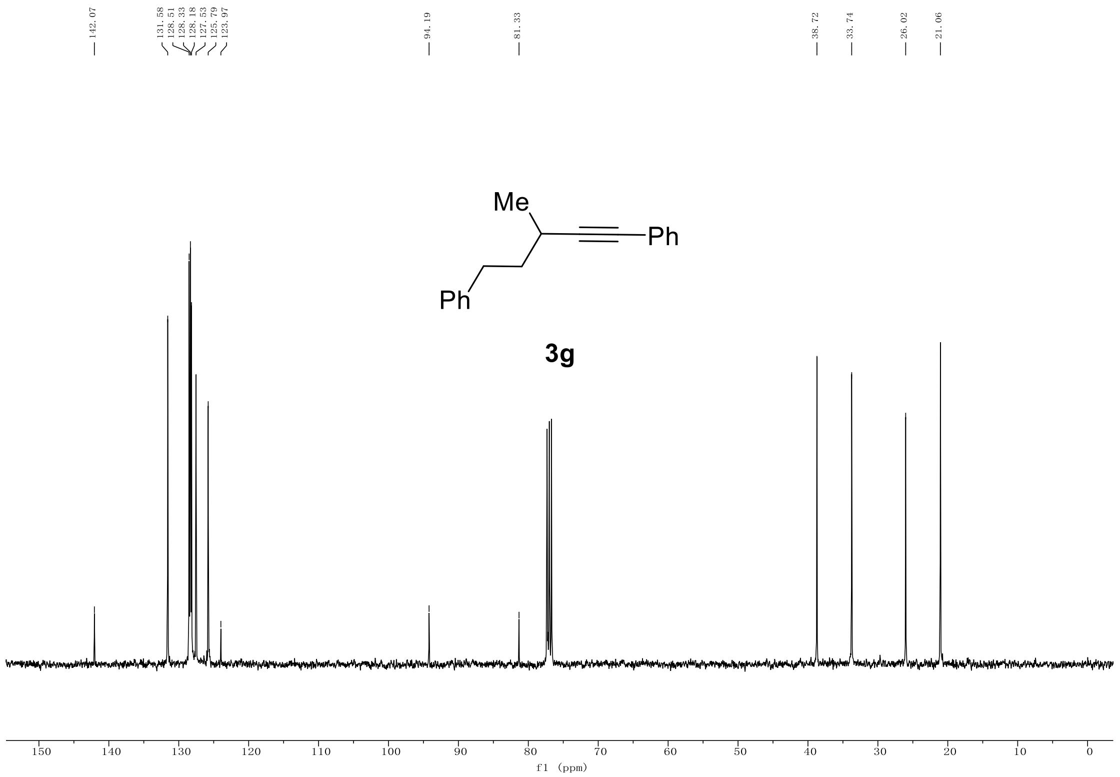
142.07
131.58
128.51
128.33
128.18
127.53
125.79
123.97
94.19
81.33
38.72
33.74
26.02
21.06
Me
Ph
Ph
3g
f1 (ppm)

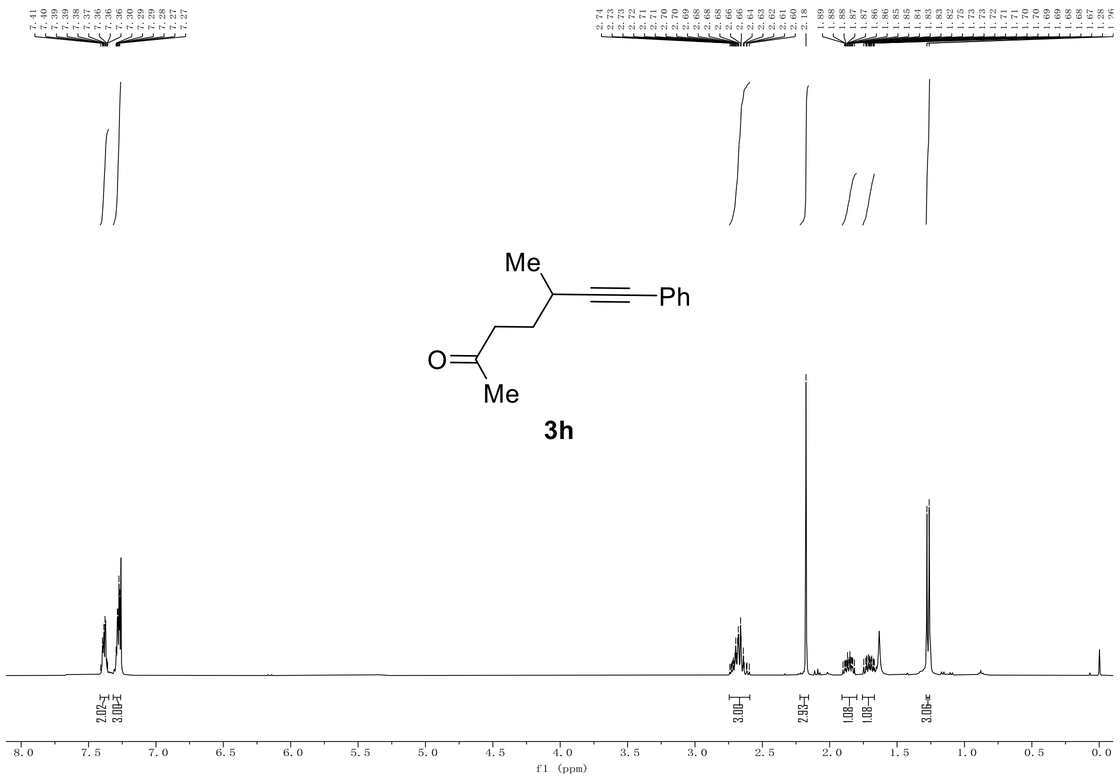
Me
Ph
O
Me
3h
f1 (ppm)

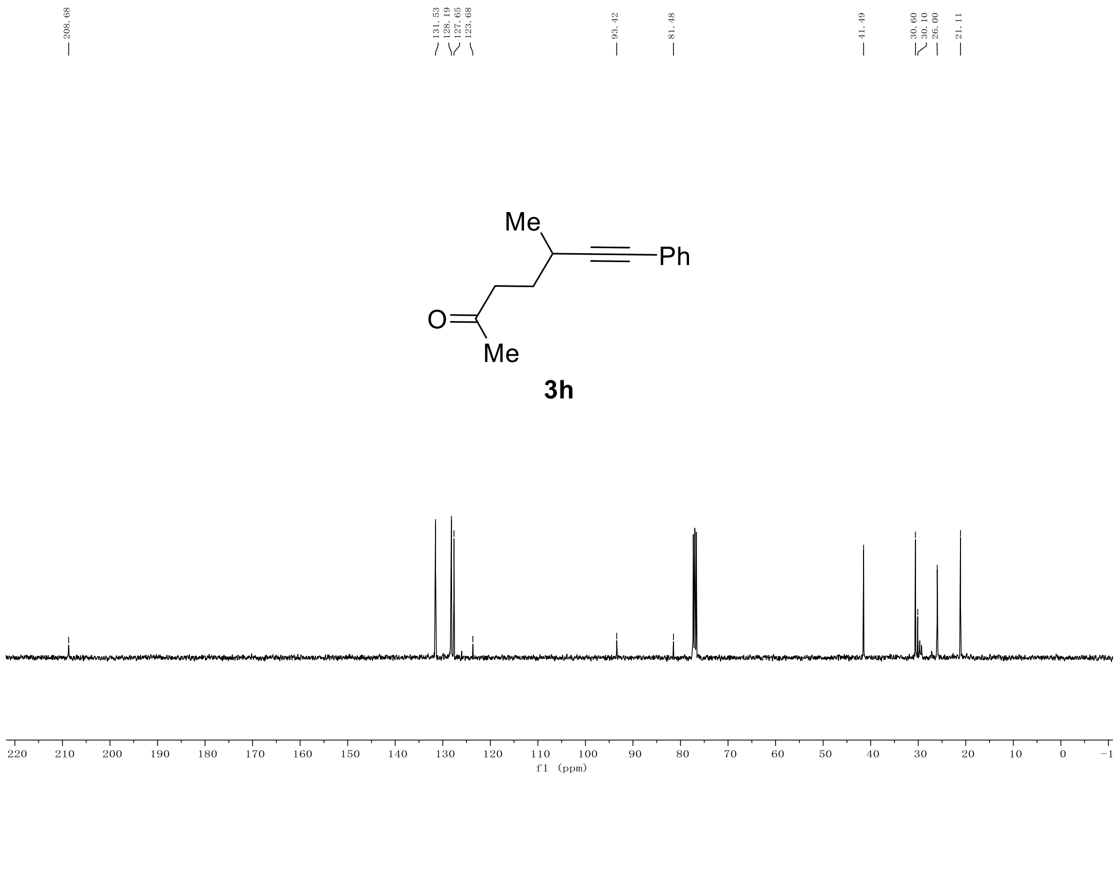
208.68
131.53
128.19
127.65
123.68
93.42
81.48
41.49
30.60
30.10
26.00
21.11
Me
Ph
O
Me
3h
f1 (ppm)

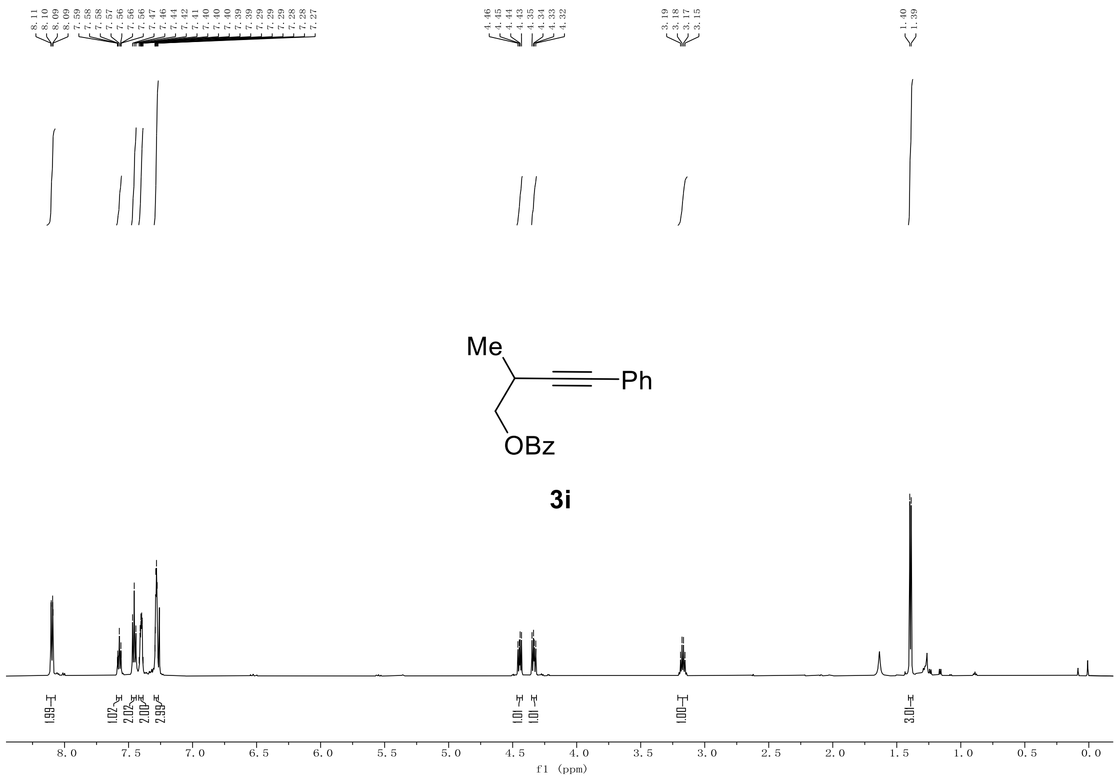
8.11 8.10 8.09 8.09 7.59 7.58 7.57 7.56 7.56 7.56 7.47 7.46 7.44 7.42 7.41 7.40 7.40 7.40 7.39 7.39 7.29 7.29 7.29 7.29 7.28 7.28 7.27
4.46 4.45 4.44 4.43 4.35 4.34 4.33 4.32
3.19 3.18 3.17 3.15
1.40 1.39
Me
Ph
OBz
3i
1.99 1.02 2.02 2.00 2.99 1.01 1.01 1.00 3.01
8.0 7.5 7.0 6.5 6.0 5.5 5.0 4.5 4.0 3.5 3.0 2.5 2.0 1.5 1.0 0.5 0.0
f1 (ppm)

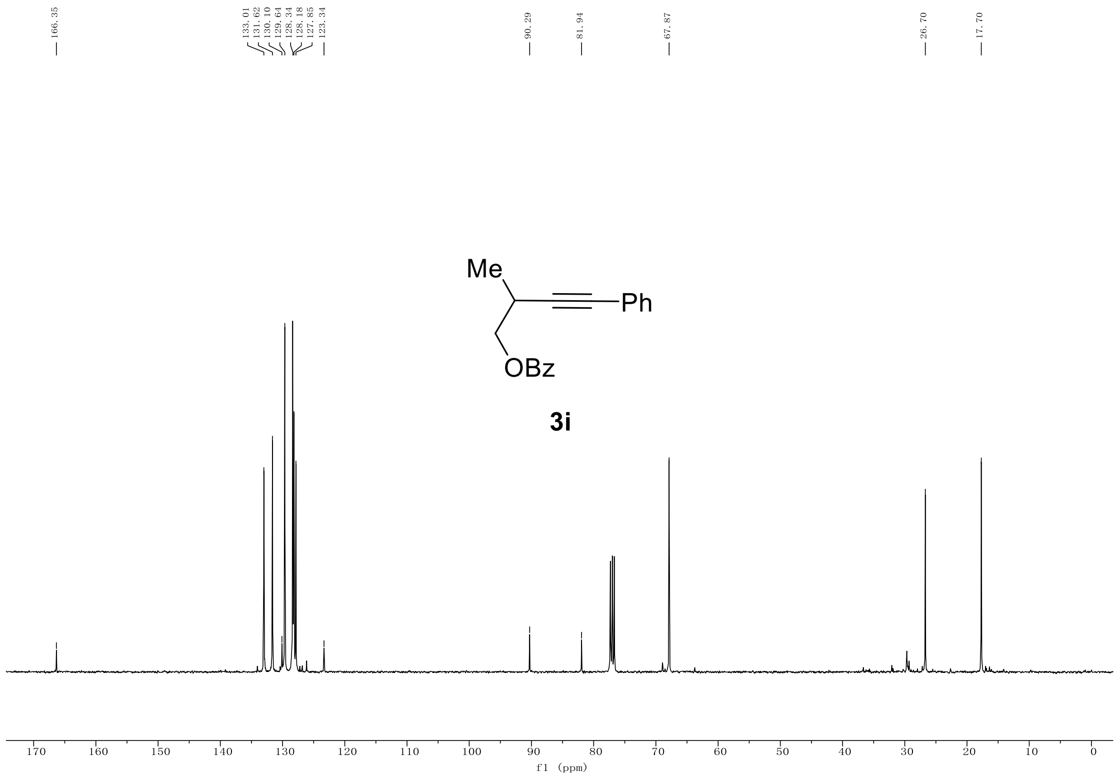
166.35
133.01 131.62 130.10 129.64 128.34 128.18 127.85 123.34
90.29
81.94
67.87
26.70
17.70
Me
Ph
OBz
3i
170 160 150 140 130 120 110 100 90 80 70 60 50 40 30 20 10 0
f1 (ppm)

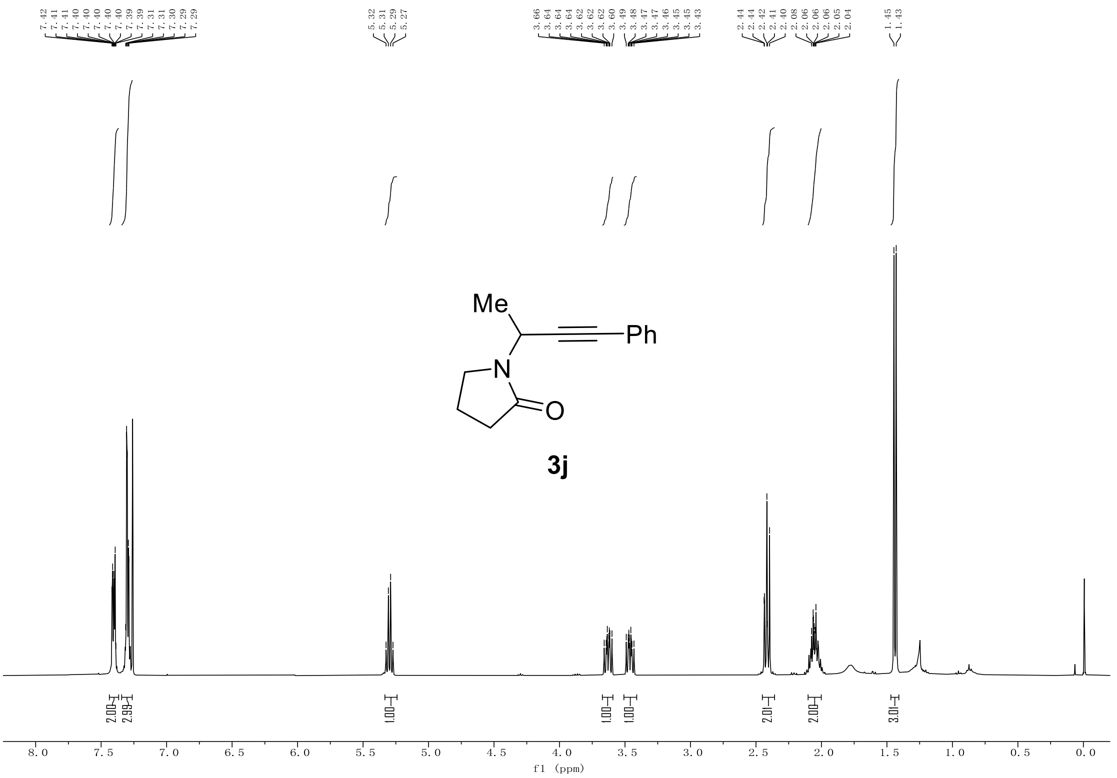
7.42 7.41 7.40 7.40 7.40 7.40 7.40 7.39 7.39 7.31 7.31 7.31 7.30 7.29 7.29
5.32 5.31 5.29 5.27
3.66 3.64 3.64 3.62 3.62 3.62 3.60 3.49 3.48 3.47 3.47 3.46 3.45 3.45 3.43
2.44 2.44 2.42 2.41 2.40 2.08 2.06 2.06 2.06 2.05 2.04
1.45 1.43
Me
N
O
Ph
3j
2.00 2.99 1.00 1.00 1.00 2.01 2.00 3.01
8.0 7.5 7.0 6.5 6.0 5.5 5.0 4.5 4.0 3.5 3.0 2.5 2.0 1.5 1.0 0.5 0.0
f1 (ppm)

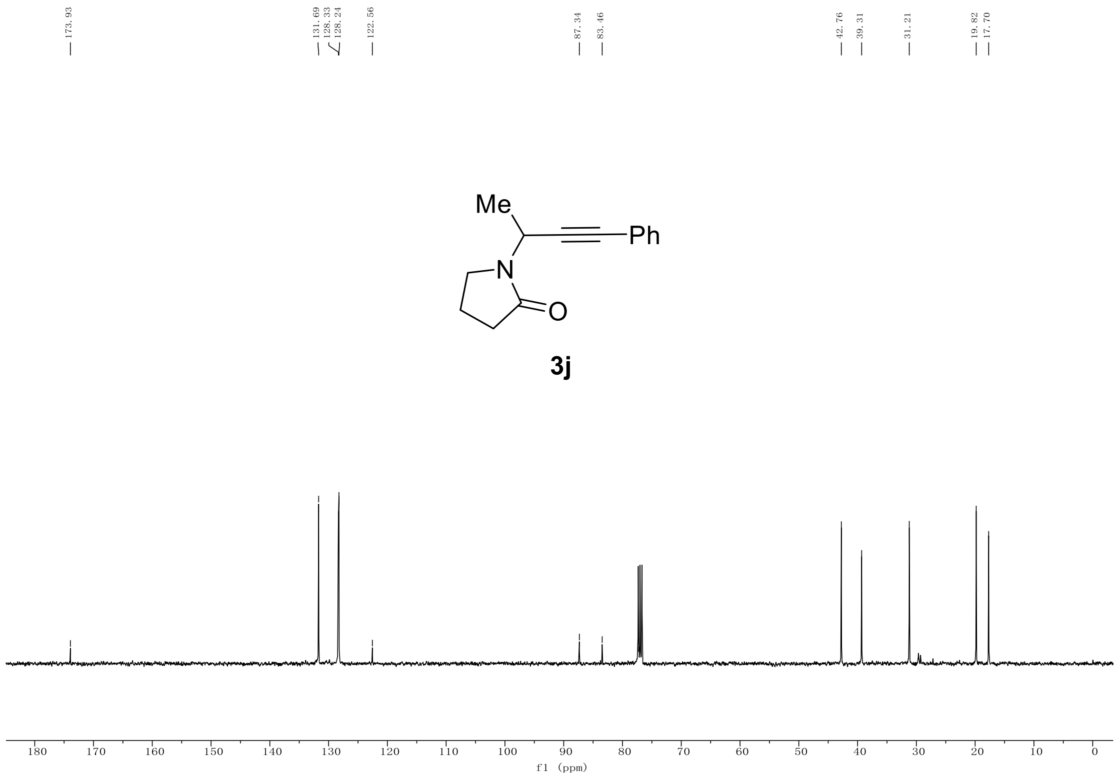
173.93
131.69 128.33 128.24
122.56
87.34 83.46
42.76 39.31
31.21
19.82 17.70
Me
N
O
Ph
3j
180 170 160 150 140 130 120 110 100 90 80 70 60 50 40 30 20 10 0
f1 (ppm)

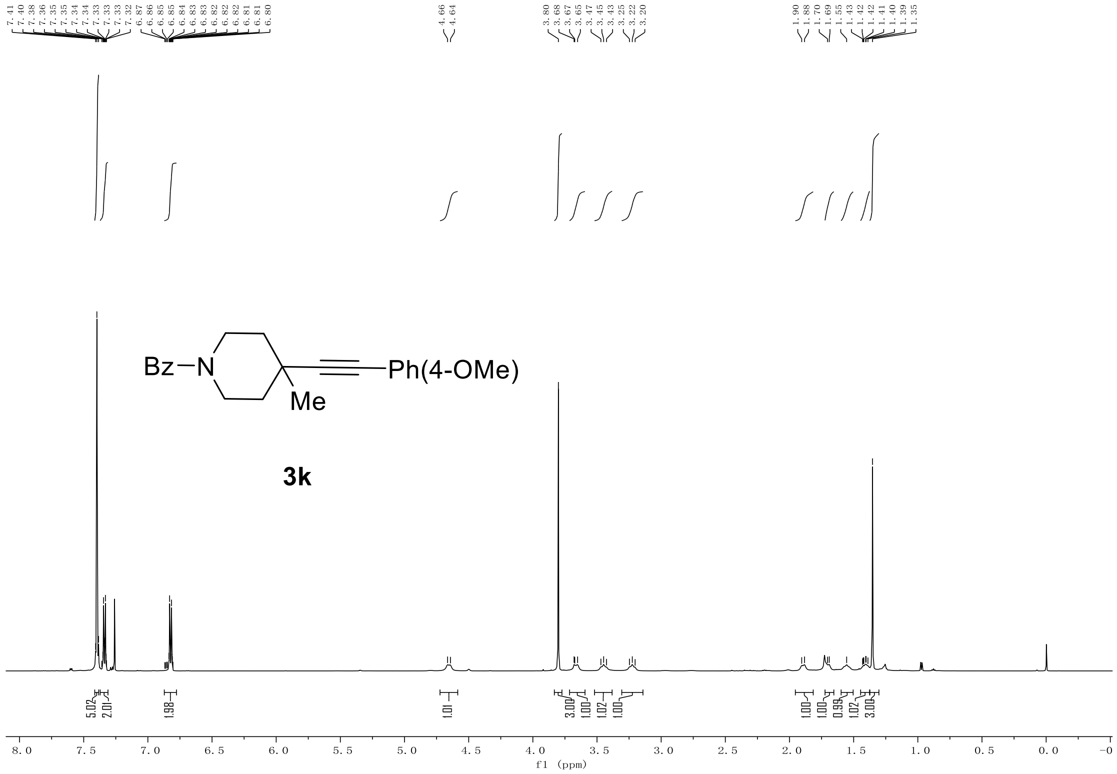
7.41 7.40 7.38 7.36 7.35 7.35 7.34 7.34 7.33 7.33 7.33 7.32 6.87 6.86 6.85 6.85 6.84 6.83 6.83 6.82 6.82 6.82 6.81 6.80
4.66 4.64
3.80 3.68 3.67 3.65 3.47 3.45 3.43 3.25 3.22 3.20
1.90 1.88 1.70 1.69 1.55 1.43 1.42 1.41 1.40 1.39 1.35
Bz–N
Ph(4-OMe)
Me
3k
5.02 2.01 1.98 1.01 3.00 1.00 1.02 1.00 1.00 1.00 0.99 1.02 3.04
f1 (ppm)

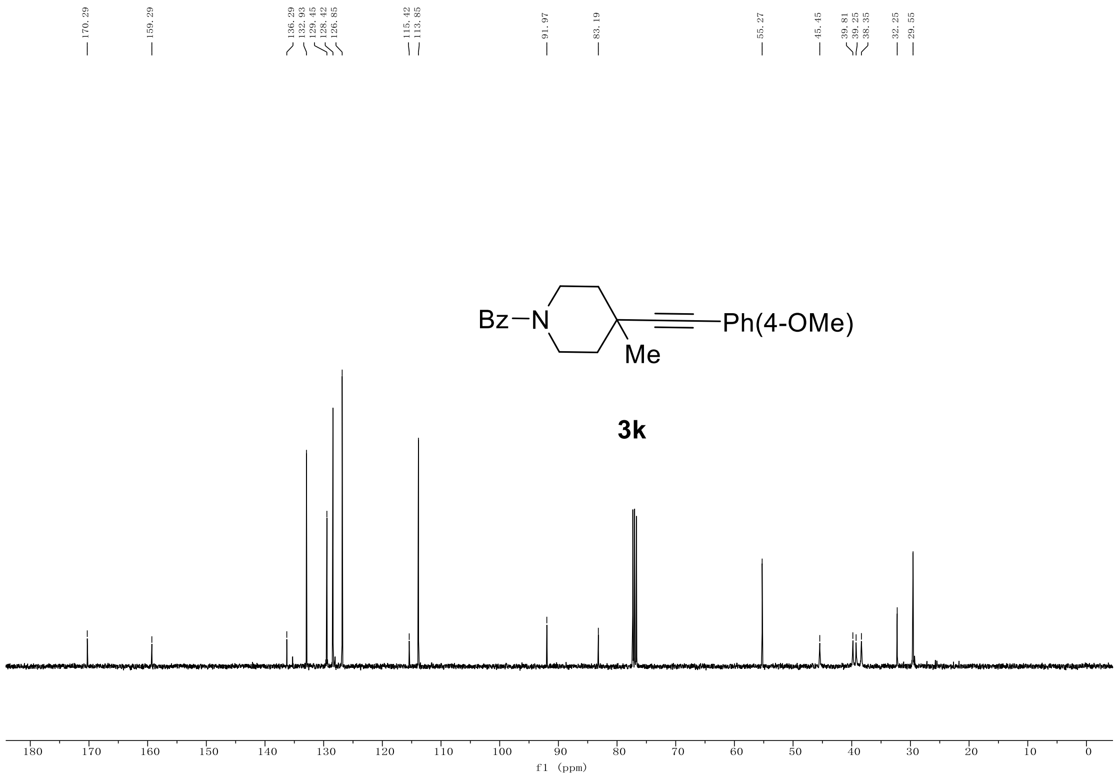
170.29
159.29
136.29 132.93 129.45 128.42 126.85
115.42 113.85
91.97
83.19
55.27
45.45
39.81 39.25 38.35
32.25 29.55
Bz–N
Ph(4-OMe)
Me
3k
f1 (ppm)

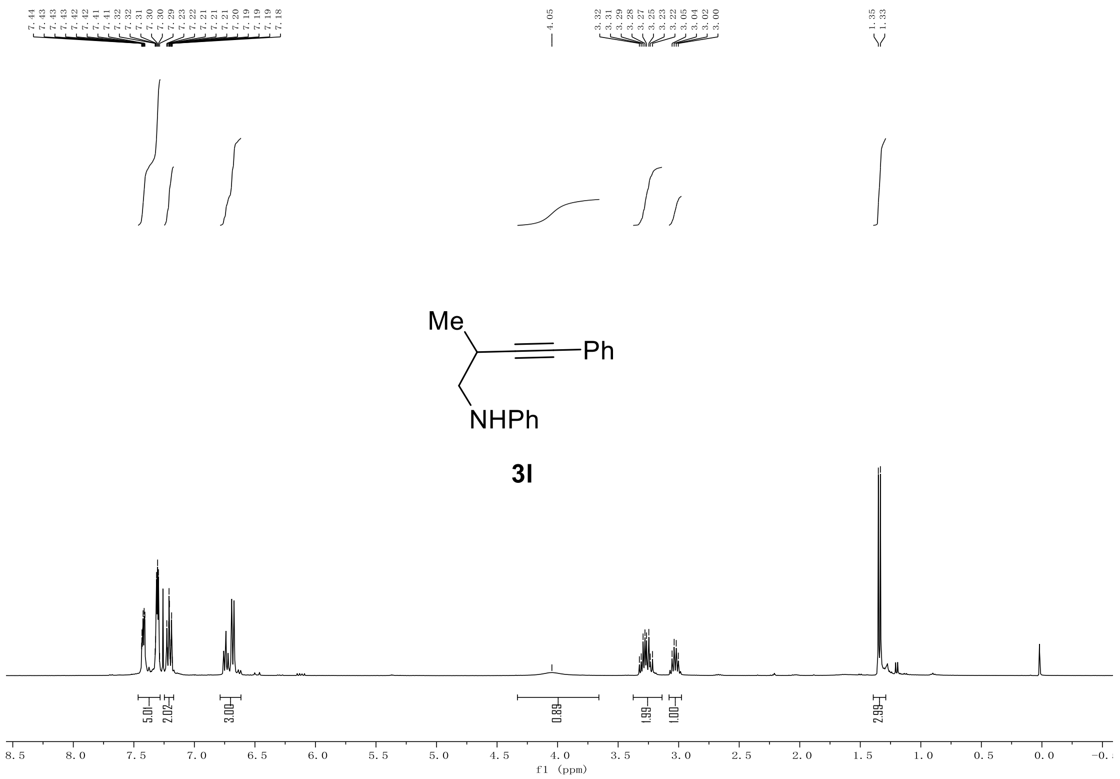
7.44 7.43 7.43 7.42 7.42 7.41 7.41 7.32 7.32 7.31 7.30 7.30 7.29 7.23 7.22 7.21 7.21 7.21 7.20 7.19 7.19 7.18
4.05
3.32 3.31 3.29 3.28 3.27 3.25 3.23 3.22 3.05 3.04 3.02 3.00
1.35 1.33
Me
Ph
NHPh
3l
5.01 2.02 3.00 0.89 1.99 1.00 2.99
f1 (ppm)

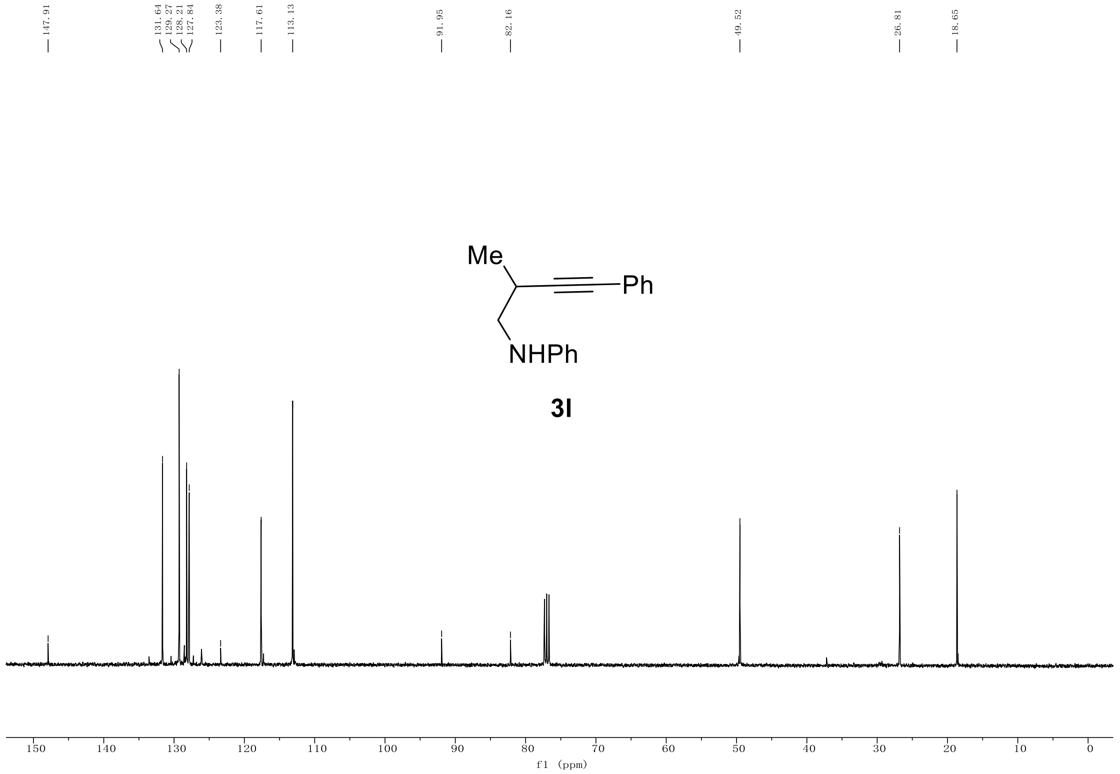
147.91
131.64 129.27 128.21 127.84
123.38
117.61
113.13
91.95
82.16
49.52
26.81
18.65
Me
Ph
NHPh
3l
f1 (ppm)

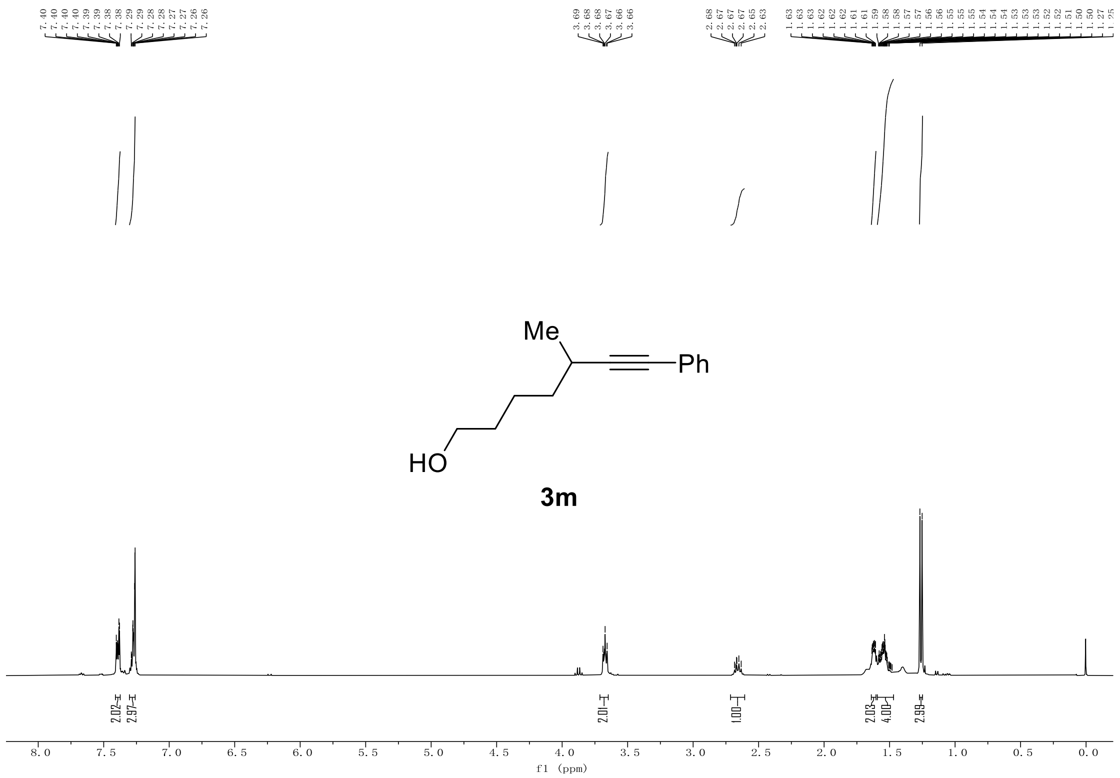
Me
Ph
HO
3m
f1 (ppm)

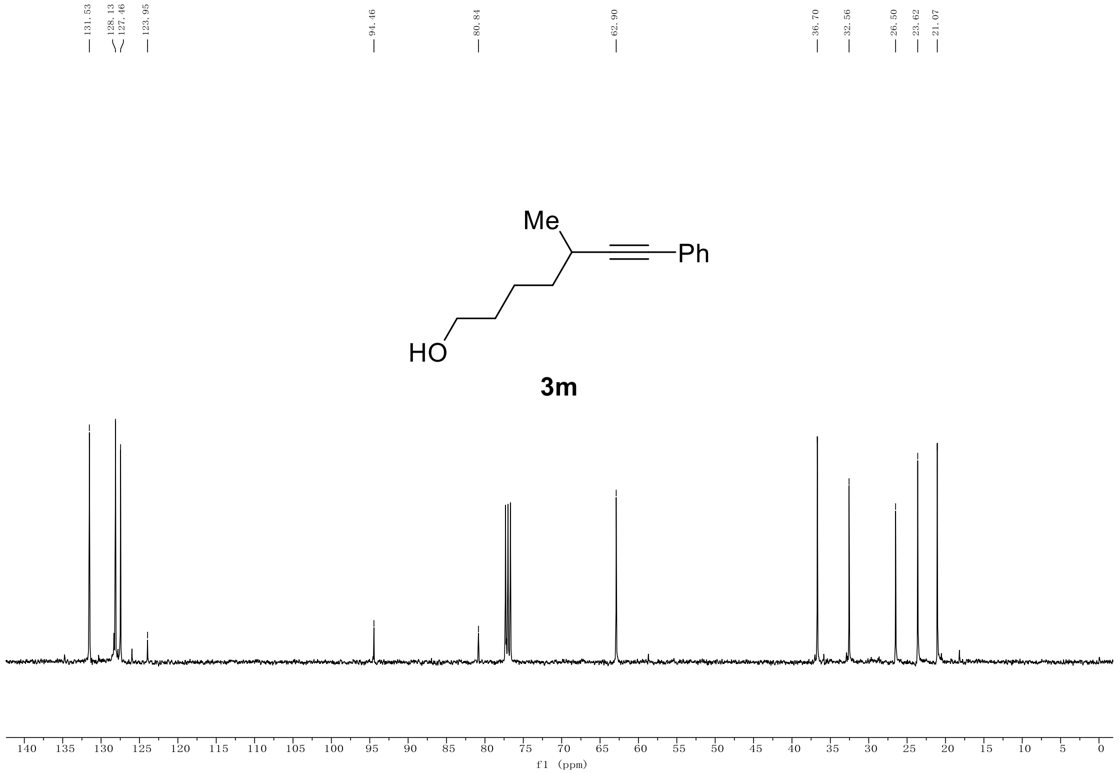
131.53
128.13
127.46
123.95
94.46
80.84
62.90
36.70
32.56
26.50
23.62
21.07
Me
Ph
HO
3m
f1 (ppm)

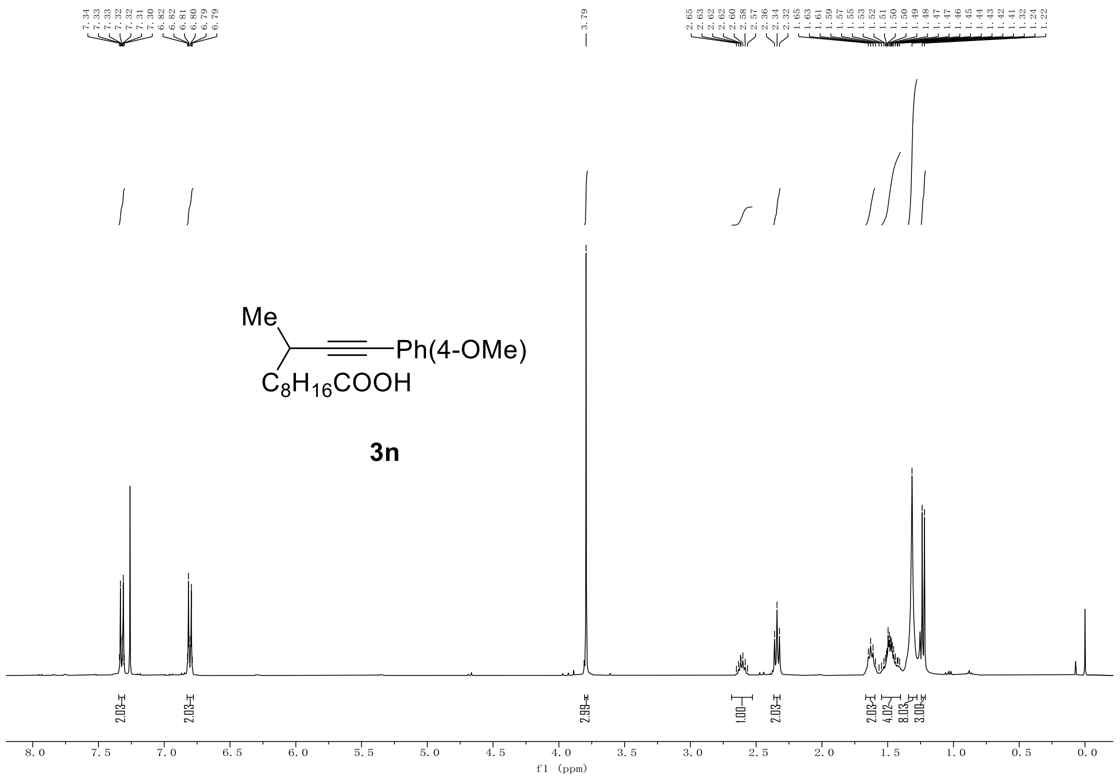
Me
Ph(4-OMe)
$C_8H_{16}COOH$
3n
f1 (ppm)

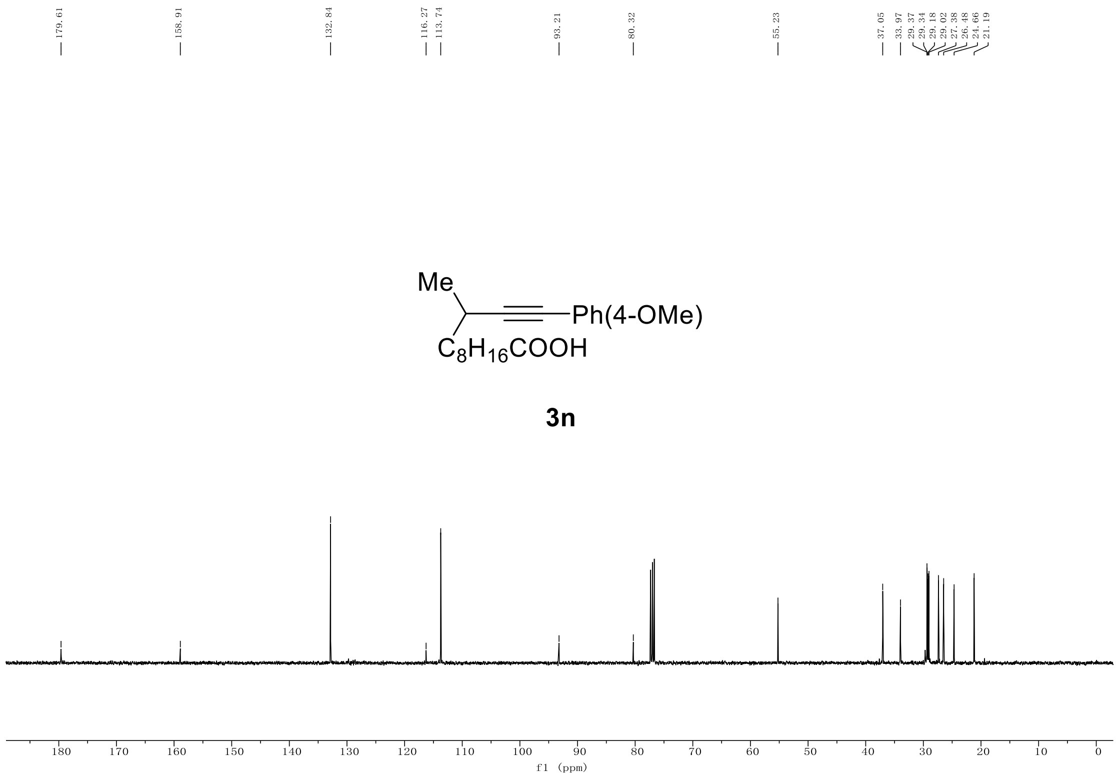
179.61
158.91
132.84
116.27
113.74
93.21
80.32
55.23
37.05
33.97
29.37
29.34
29.18
29.02
27.38
26.48
24.66
21.19
Me
Ph(4-OMe)
$C_8H_{16}COOH$
3n
f1 (ppm)

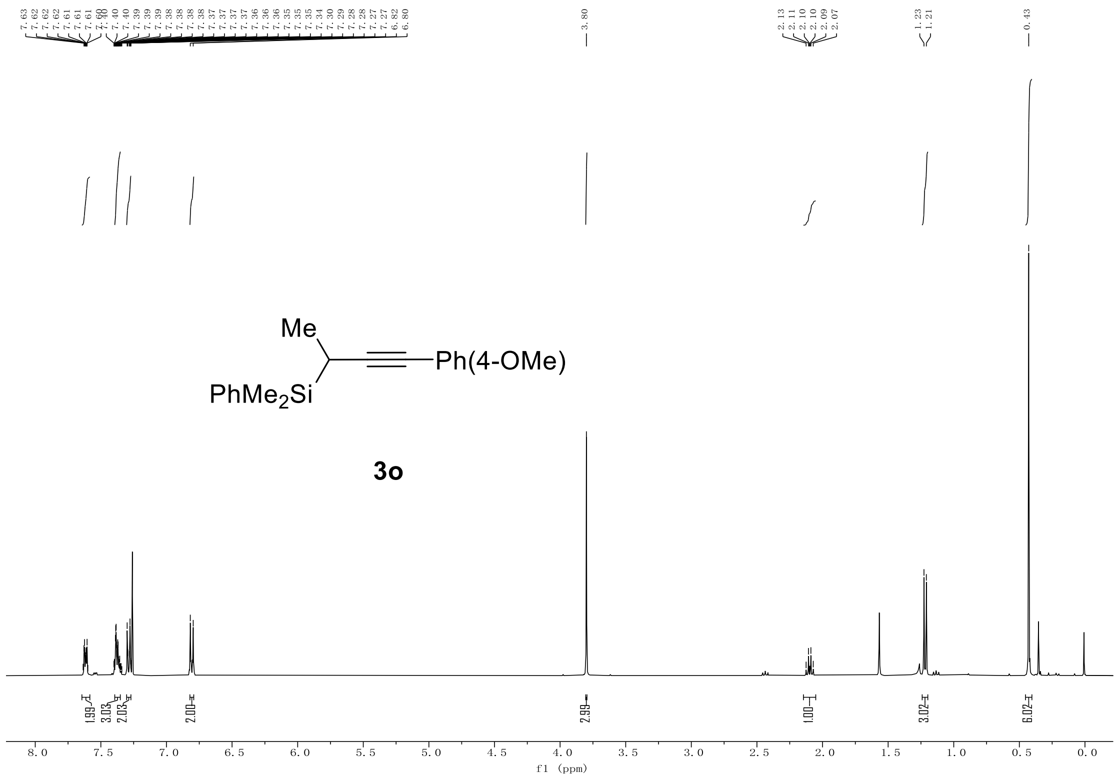
Me
PhMe2Si
Ph(4-OMe)
3o
3.80
2.13
2.11
2.10
2.10
2.09
2.07
1.23
1.21
0.43
1.99
3.03
2.03
2.00
2.99
1.00
3.02
6.02
f1 (ppm)

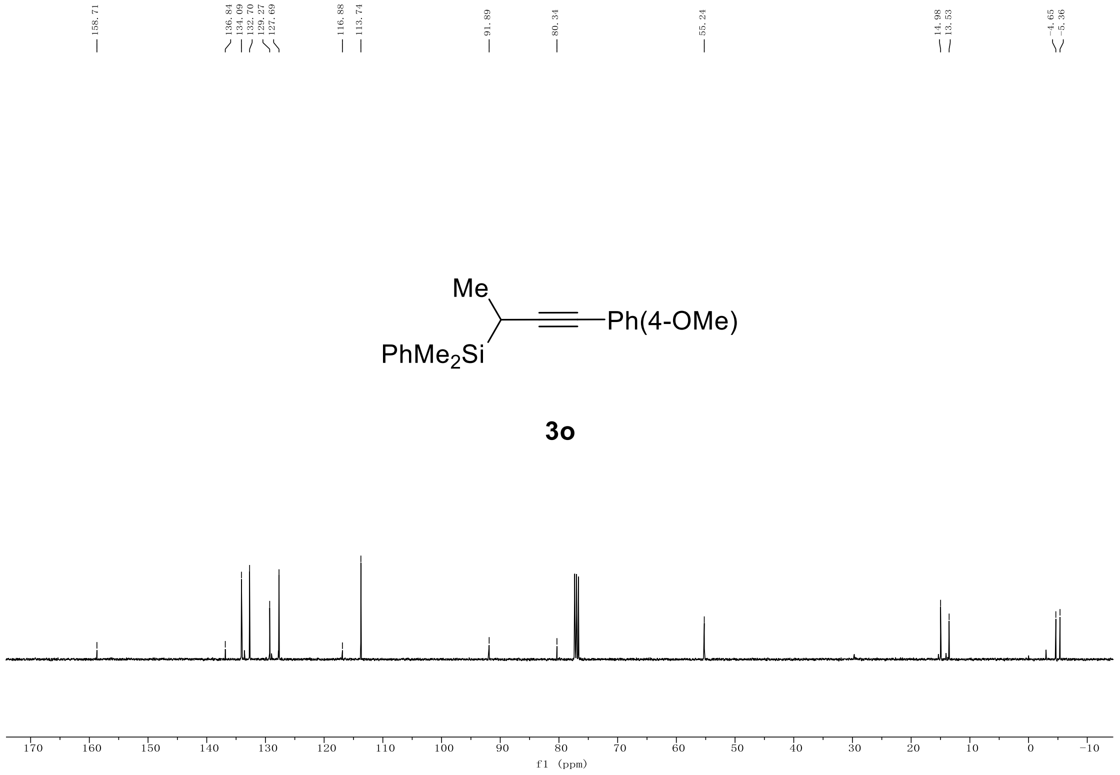
158.71
136.84
134.09
132.70
129.27
127.69
116.88
113.74
91.89
80.34
55.24
14.98
13.53
-4.65
-5.36
Me
PhMe2Si
Ph(4-OMe)
3o
f1 (ppm)

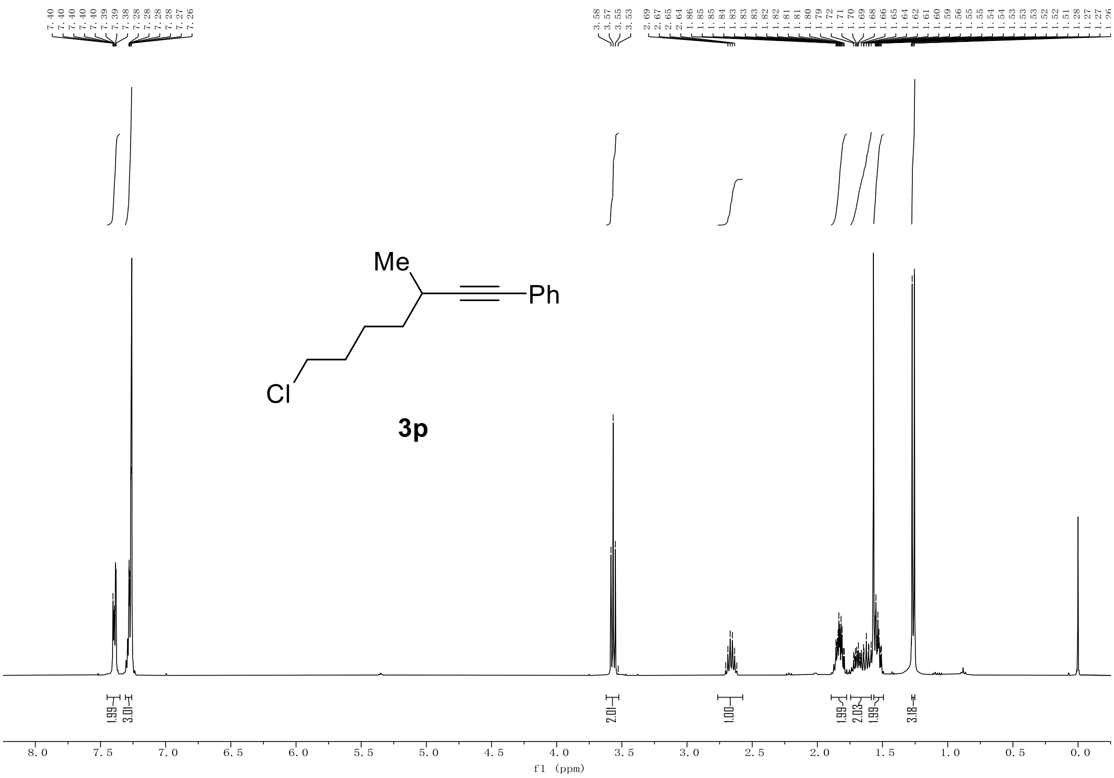
7.40 7.40 7.40 7.40 7.39 7.38 7.28 7.28 7.28 7.28 7.27 7.26
3.58 3.57 3.55 3.53
2.69 2.67 2.65 2.64 1.86 1.85 1.85 1.84 1.83 1.83 1.82 1.82 1.81 1.81 1.80 1.79 1.72 1.71 1.70 1.69 1.68 1.66 1.65 1.64 1.62 1.61 1.60 1.59 1.56 1.55 1.55 1.54 1.53 1.53 1.52 1.51 1.28 1.27 1.26
Me
Ph
Cl
3p
1.99 3.01 2.01 1.00 1.99 2.03 1.99 3.18
8.0 7.5 7.0 6.5 6.0 5.5 5.0 4.5 4.0 3.5 3.0 2.5 2.0 1.5 1.0 0.5 0.0
f1 (ppm)

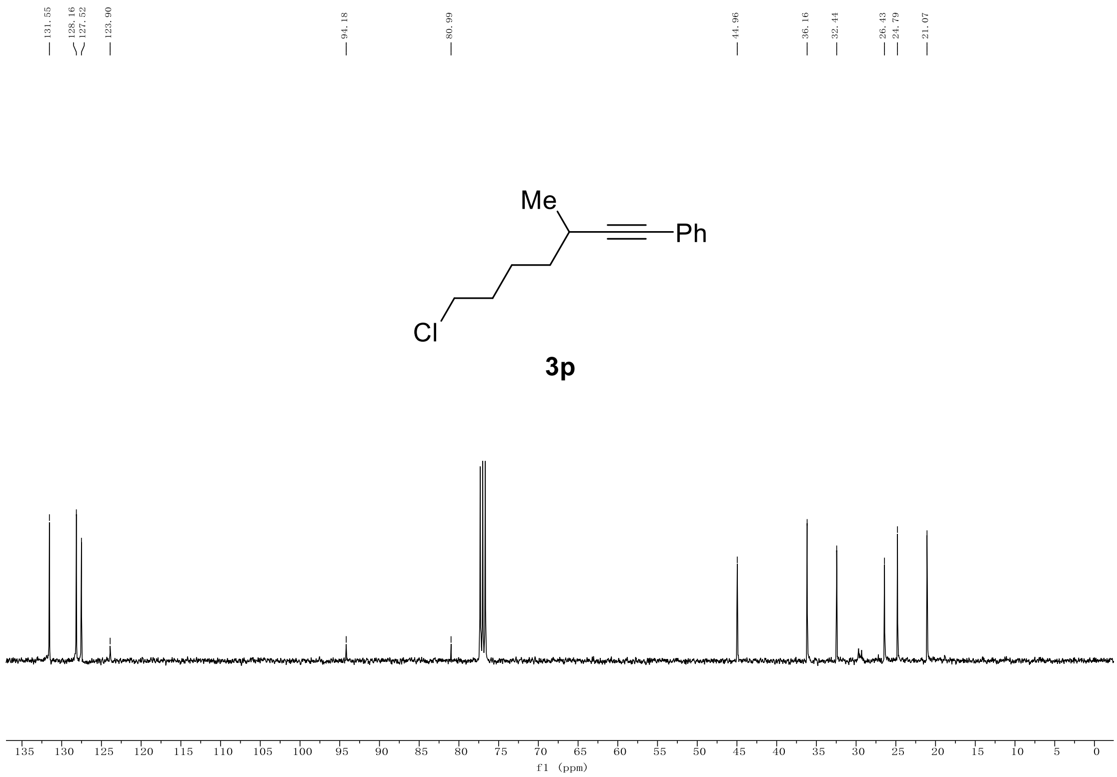
131.55 128.16 127.52 123.90
94.18
80.99
44.96
36.16 32.44
26.43 24.79
21.07
Me
Ph
Cl
3p
135 130 125 120 115 110 105 100 95 90 85 80 75 70 65 60 55 50 45 40 35 30 25 20 15 10 5 0
f1 (ppm)

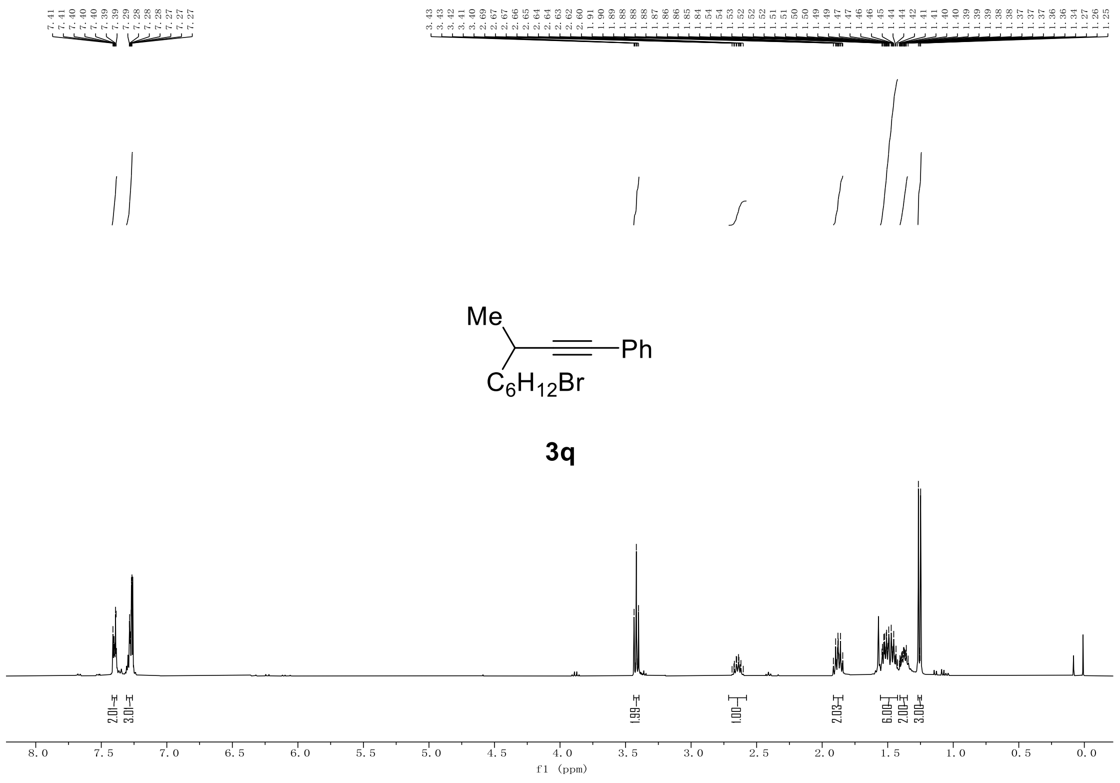
7.41
7.41
7.40
7.40
7.39
7.39
7.29
7.28
7.28
7.28
7.27
7.27
7.27
3.43
3.43
3.42
3.41
3.40
2.69
2.67
2.67
2.66
2.65
2.64
2.64
2.63
2.62
2.60
1.91
1.90
1.89
1.88
1.88
1.88
1.87
1.86
1.86
1.85
1.84
1.54
1.54
1.53
1.52
1.52
1.51
1.51
1.50
1.50
1.49
1.49
1.47
1.47
1.46
1.46
1.45
1.44
1.44
1.42
1.41
1.41
1.40
1.40
1.39
1.39
1.38
1.38
1.37
1.37
1.37
1.36
1.36
1.34
1.27
1.26
1.25
Me
Ph
C6H12Br
2.01
3.01
1.99
1.00
2.03
6.00
2.00
3.00
f1 (ppm)

3q

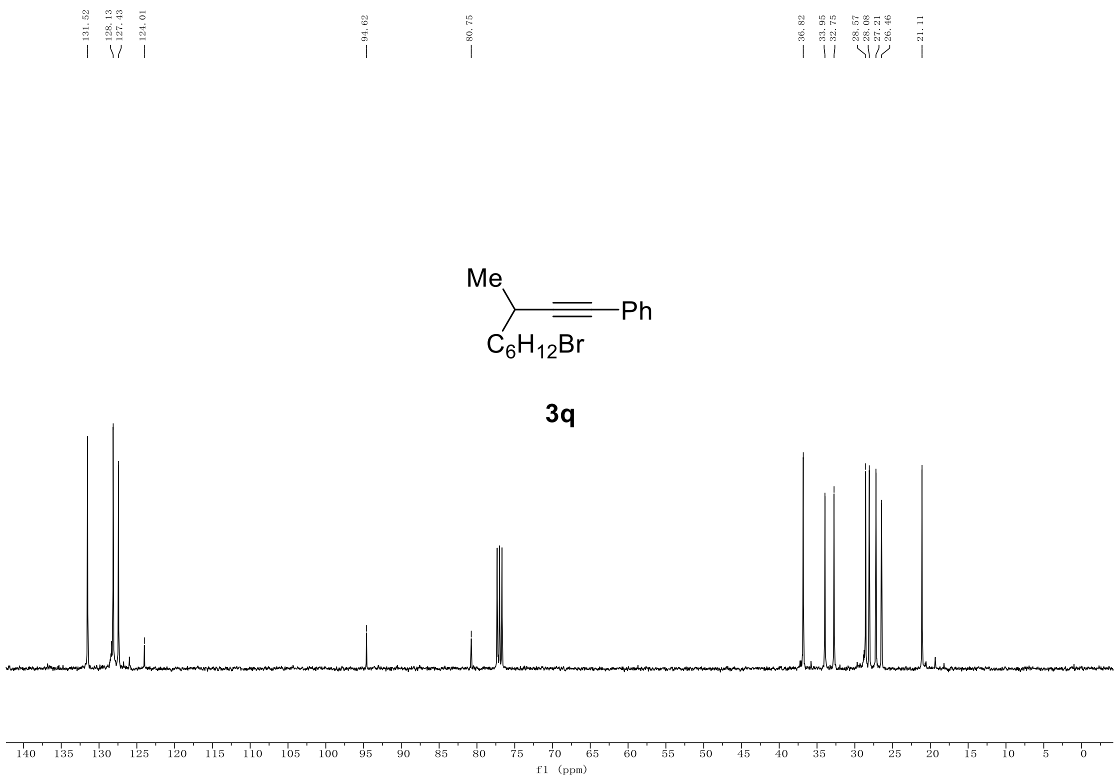
131.52
128.13
127.43
124.01
94.62
80.75
36.82
33.95
32.75
28.57
28.08
27.21
26.46
21.11
Me
Ph
C6H12Br
f1 (ppm)

3q

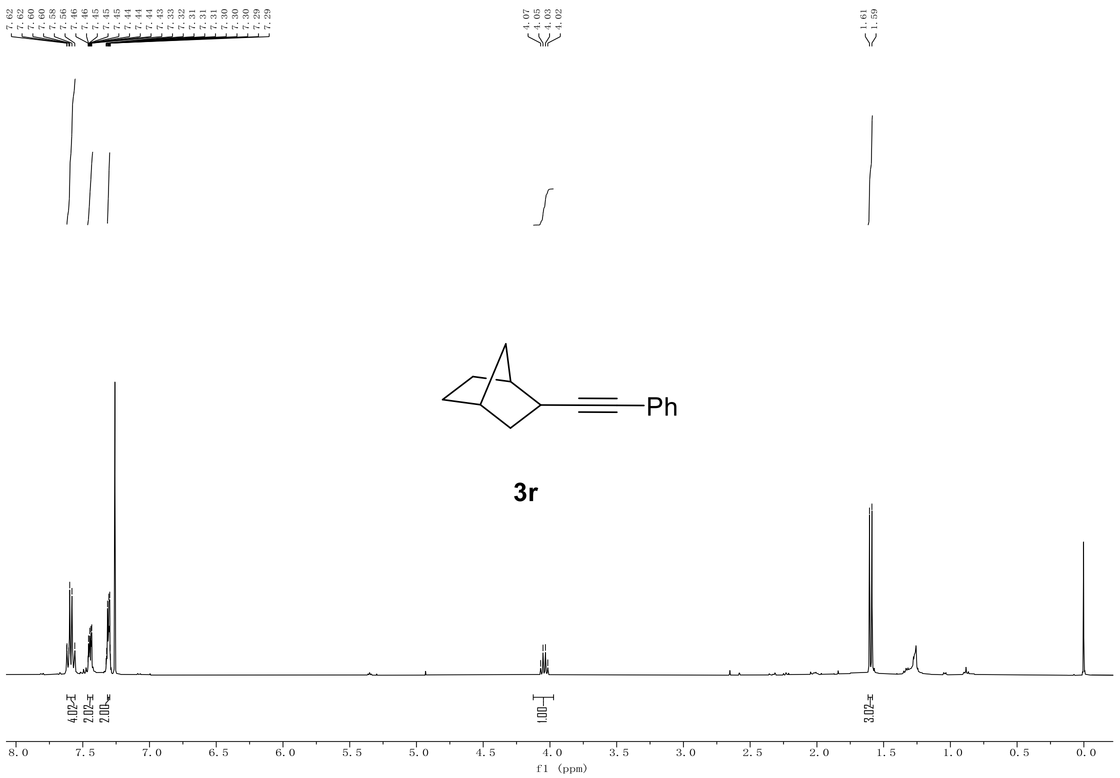
7.62 7.62 7.60 7.60 7.58 7.56 7.46 7.46 7.45 7.45 7.44 7.44 7.44 7.43 7.33 7.32 7.31 7.31 7.31 7.30 7.30 7.30 7.29 7.29
4.07 4.05 4.03 4.02
1.61 1.59
Ph
3r
4.02
2.02
2.00
1.00
3.02
8.0 7.5 7.0 6.5 6.0 5.5 5.0 4.5 4.0 3.5 3.0 2.5 2.0 1.5 1.0 0.5 0.0
f1 (ppm)

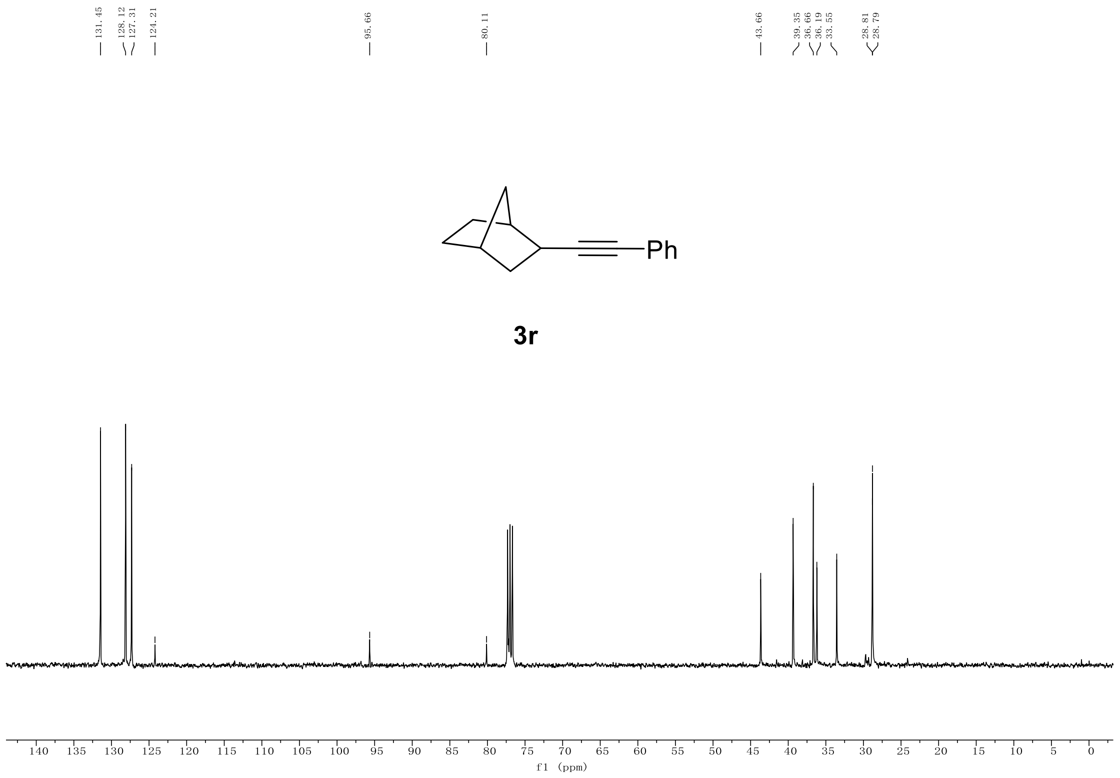
131.45
128.12
127.31
124.21
95.66
80.11
43.66
39.35
36.66
36.19
33.55
28.81
28.79
Ph
3r
140 135 130 125 120 115 110 105 100 95 90 85 80 75 70 65 60 55 50 45 40 35 30 25 20 15 10 5 0
f1 (ppm)

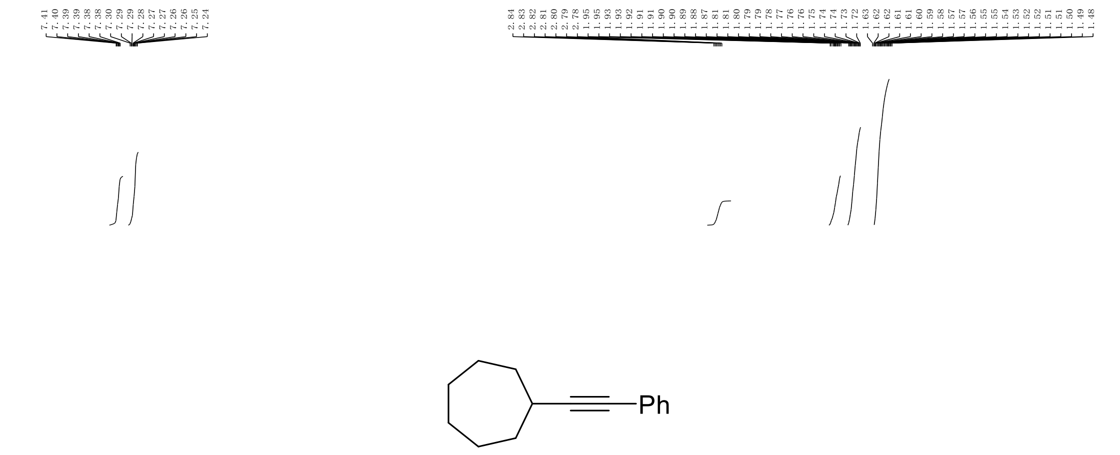
7.41 7.40 7.39 7.39 7.38 7.38 7.30 7.29 7.29 7.28 7.27 7.27 7.26 7.25 7.24
2.84 2.83 2.82 2.81 2.80 2.79 2.78 1.95 1.95 1.93 1.93 1.92 1.91 1.91 1.90 1.89 1.88 1.87 1.81 1.81 1.80 1.79 1.79 1.78 1.77 1.76 1.75 1.74 1.74 1.73 1.72 1.63 1.62 1.62 1.61 1.61 1.60 1.59 1.58 1.57 1.57 1.56 1.55 1.55 1.54 1.53 1.52 1.52 1.51 1.50 1.49 1.48
Ph

3s

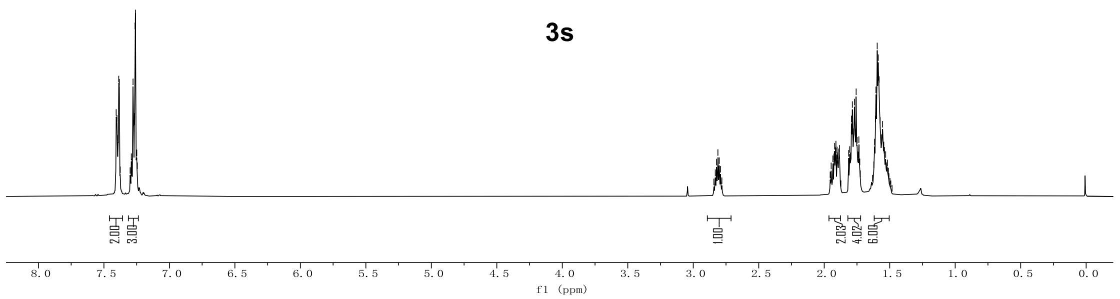
2.00
3.00
1.00
2.03
4.02
6.00
8.0 7.5 7.0 6.5 6.0 5.5 5.0 4.5 4.0 3.5 3.0 2.5 2.0 1.5 1.0 0.5 0.0
f1 (ppm)

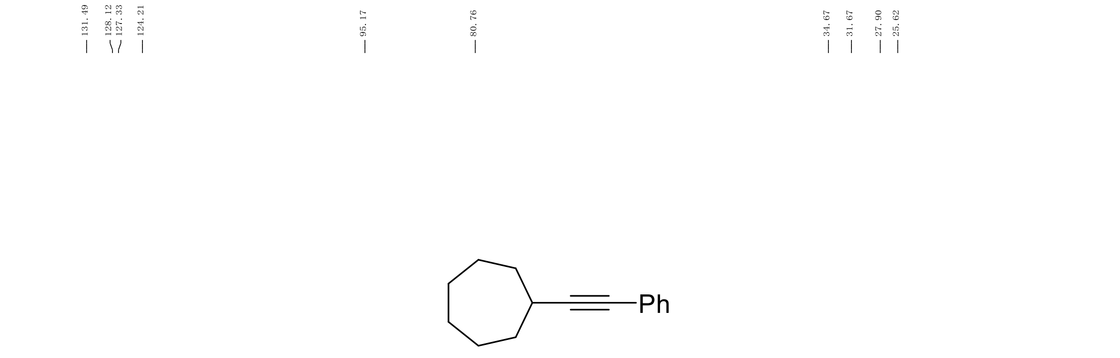
131.49
128.12
127.33
124.21
95.17
80.76
34.67
31.67
27.90
25.62
Ph

3s

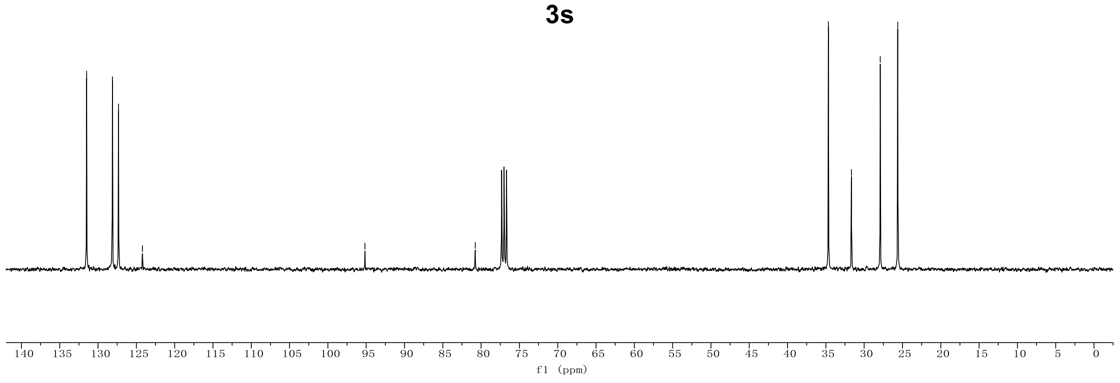
140 135 130 125 120 115 110 105 100 95 90 85 80 75 70 65 60 55 50 45 40 35 30 25 20 15 10 5 0
f1 (ppm)

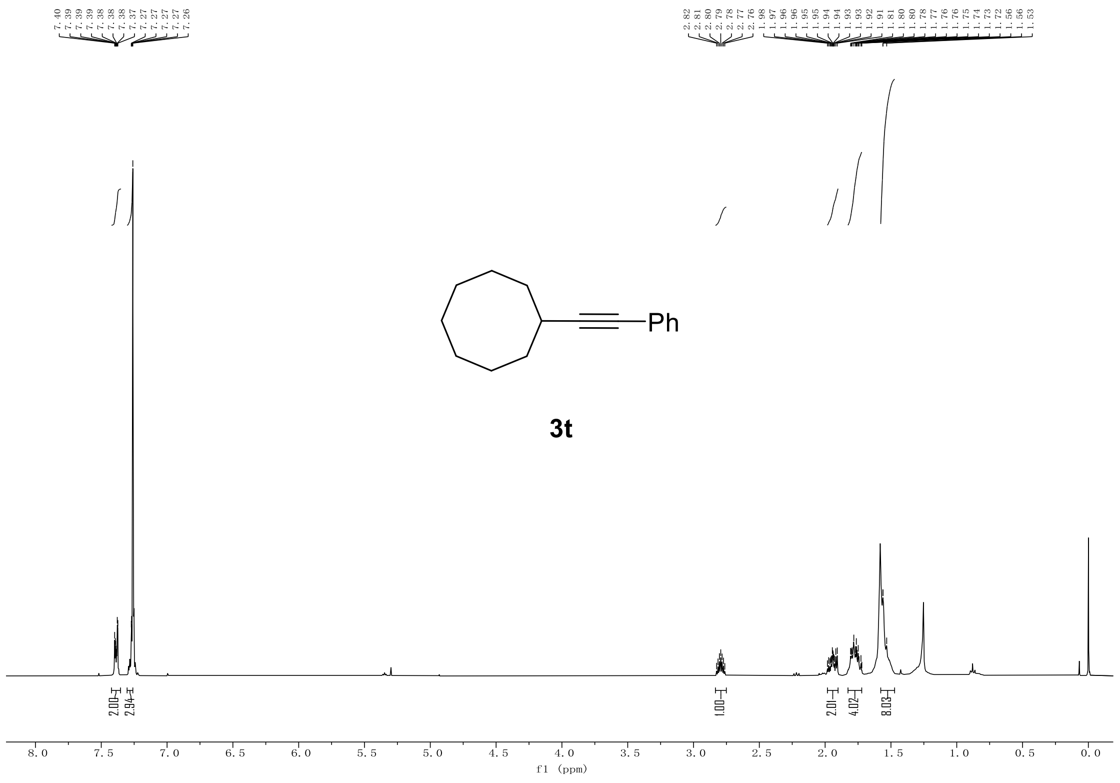

3t

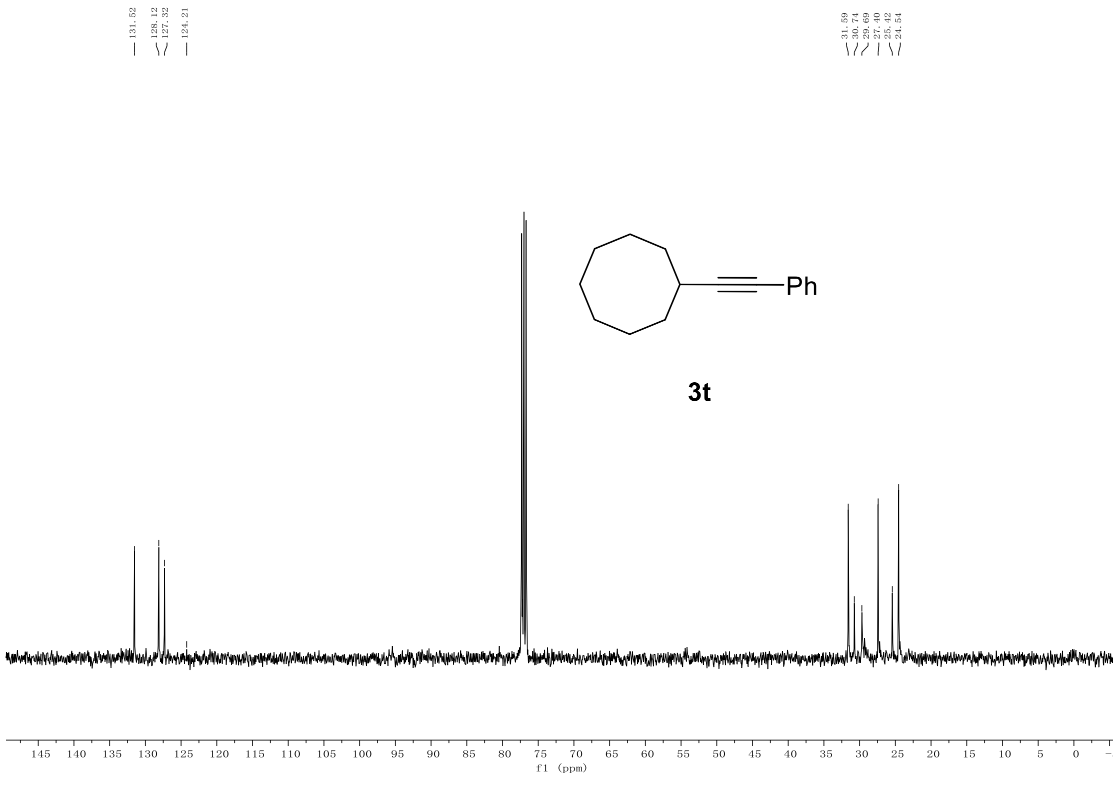

3t

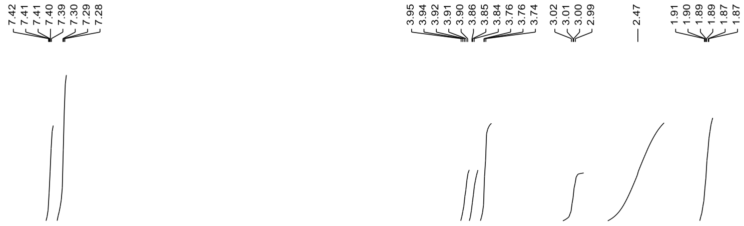

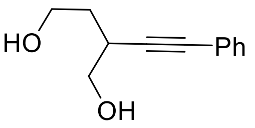

3u

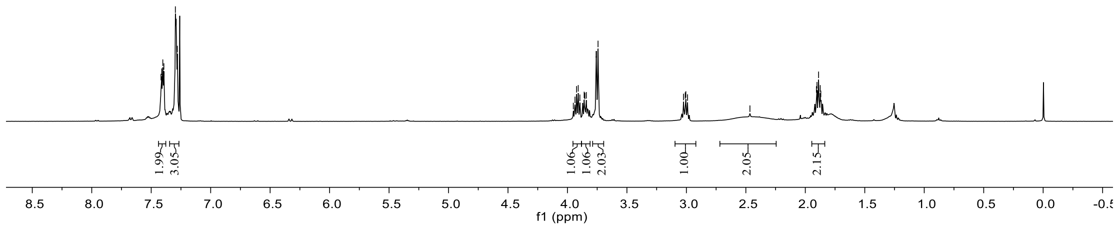

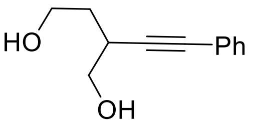

3u

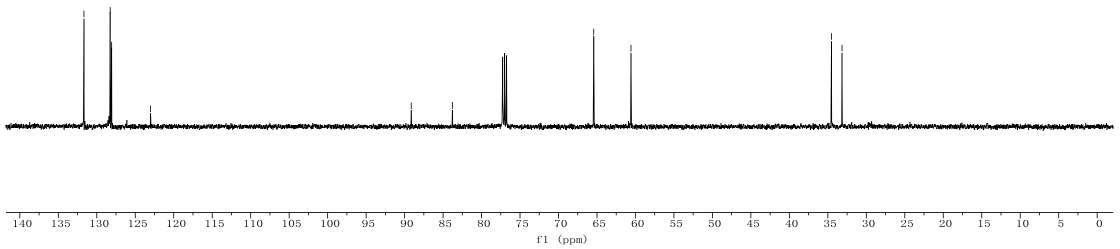

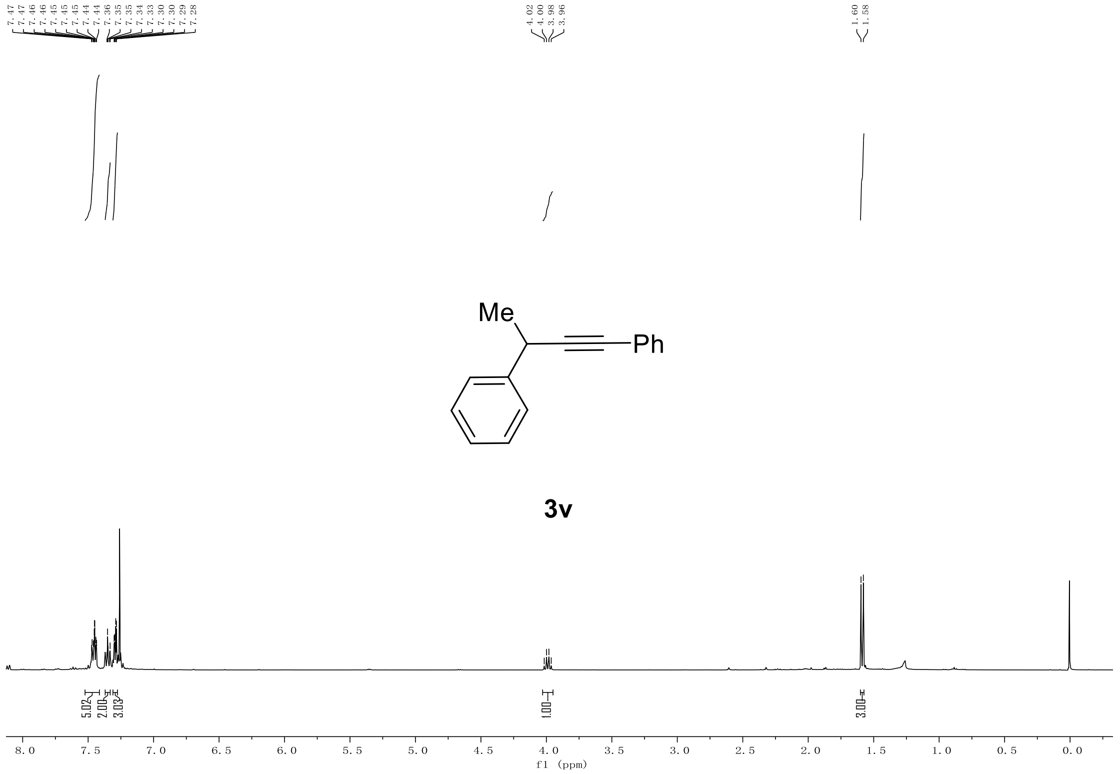
7.47
7.47
7.46
7.45
7.45
7.45
7.44
7.44
7.36
7.35
7.35
7.34
7.33
7.30
7.30
7.29
7.28
4.02
4.00
3.98
3.96
1.60
1.58
Me
Ph
3v
5.02
2.00
3.03
1.00
3.00
f1 (ppm)

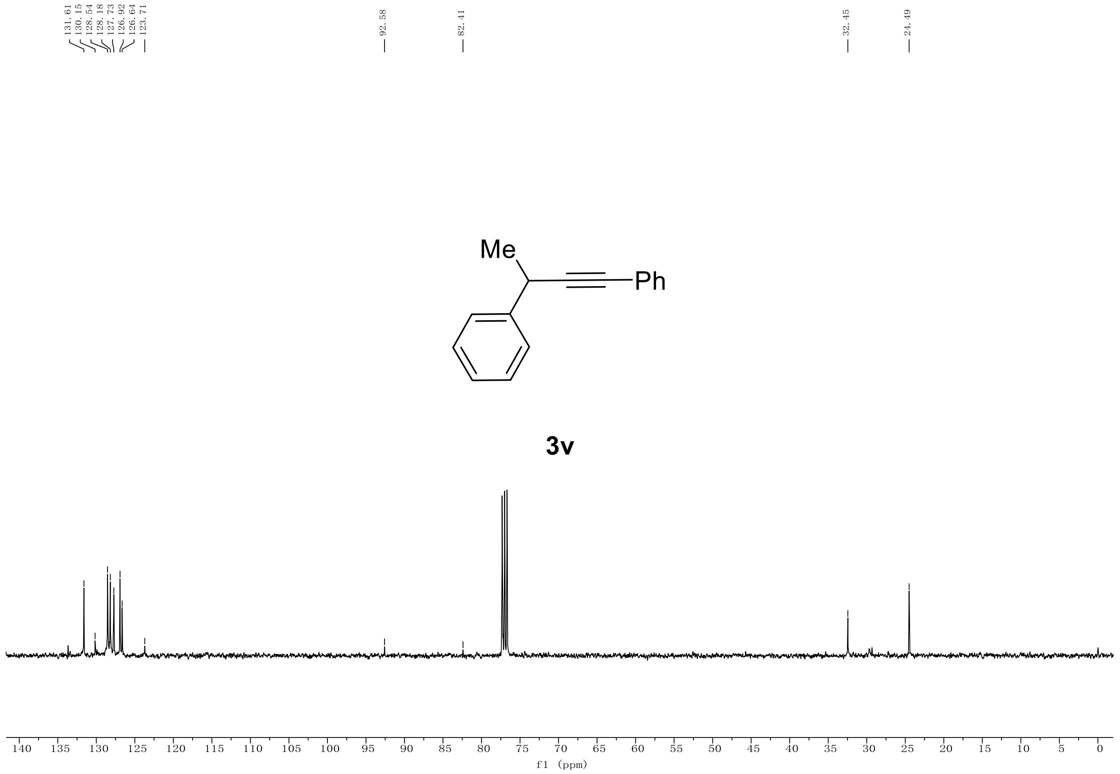
131.61
130.15
128.54
128.18
127.73
126.92
126.64
123.71
92.58
82.41
32.45
24.49
Me
Ph
3v
f1 (ppm)

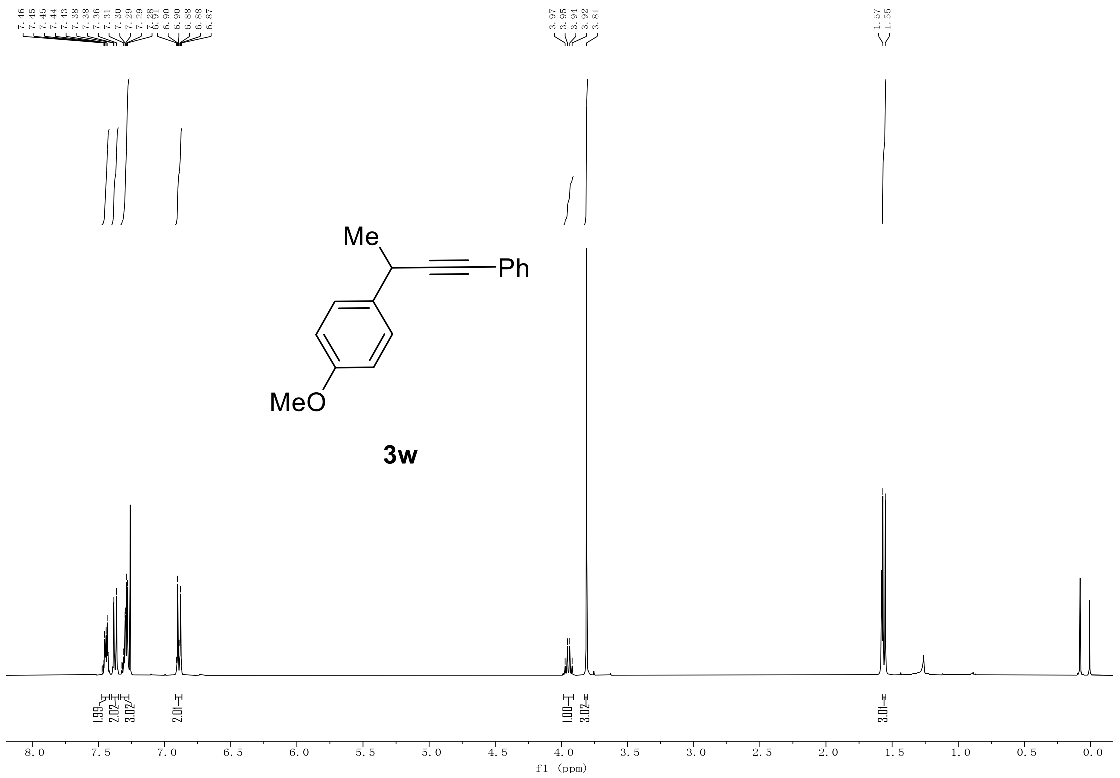
7.46
7.45
7.45
7.44
7.43
7.38
7.38
7.36
7.31
7.30
7.29
7.29
7.28
6.91
6.90
6.88
6.88
6.87
3.97
3.95
3.94
3.92
3.81
1.57
1.55
Me
Ph
MeO
3w
1.99
2.02
3.02
2.01
1.00
3.02
3.01
f1 (ppm)

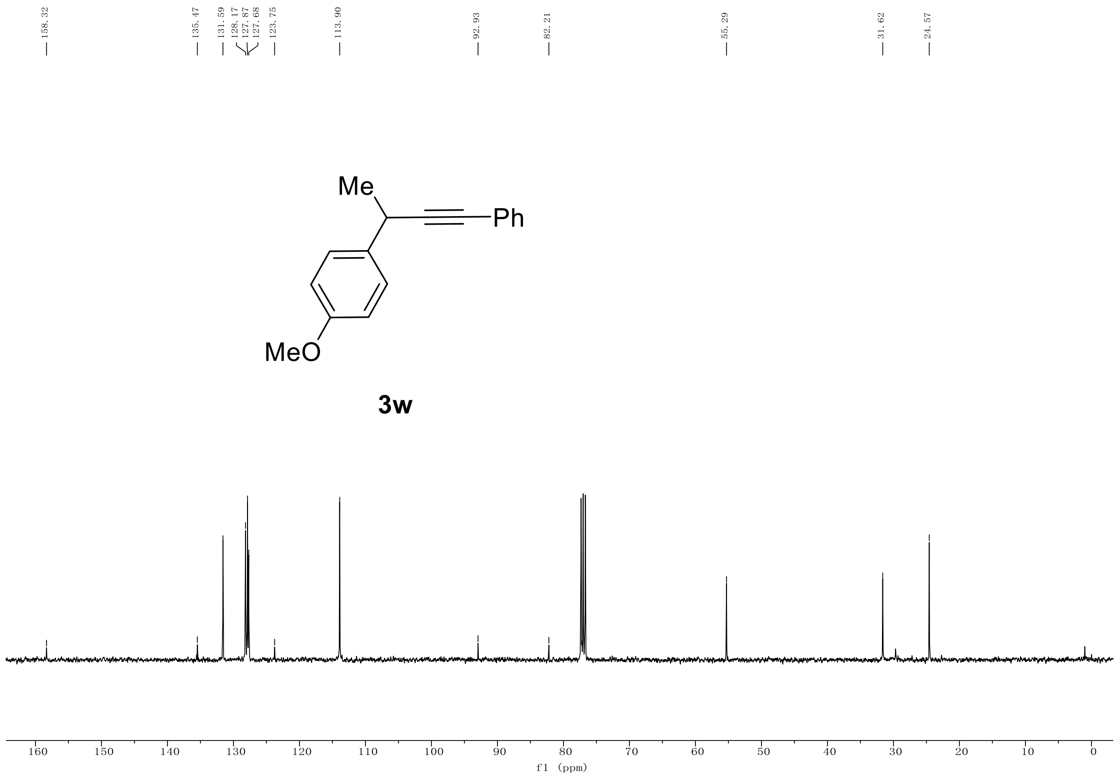
158.32
135.47
131.59
128.17
127.87
127.68
123.75
113.90
92.93
82.21
55.29
31.62
24.57
Me
Ph
MeO
3w
f1 (ppm)

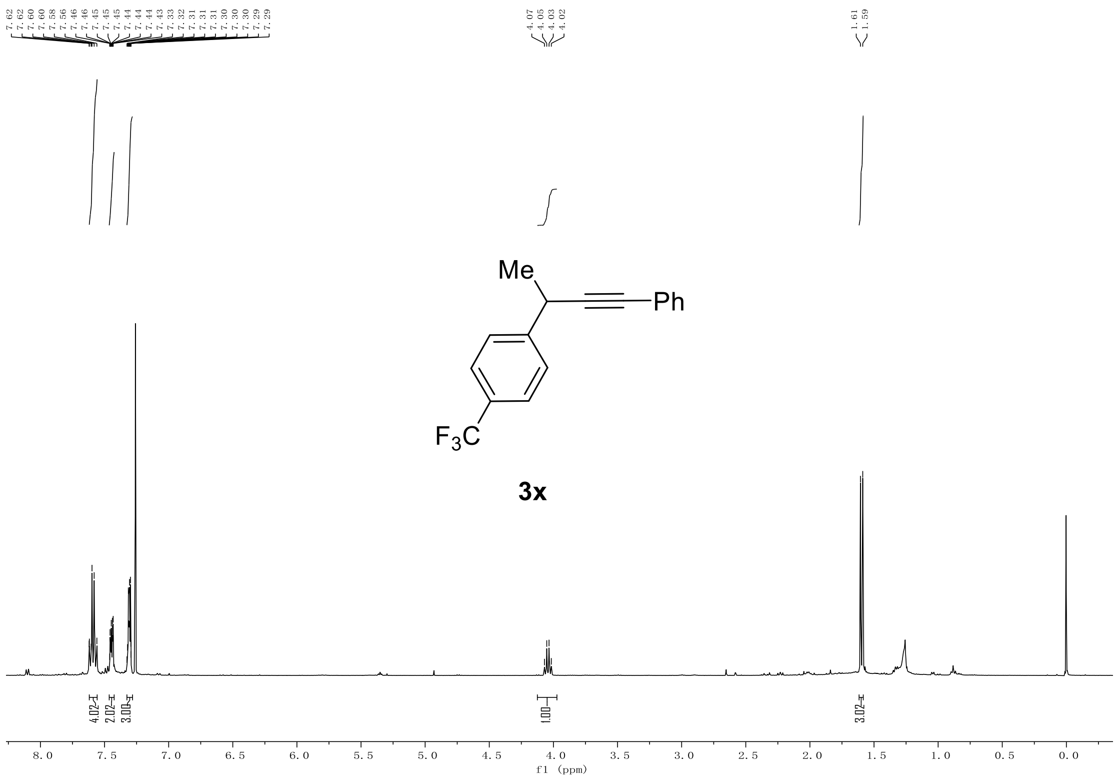
7.62
7.62
7.60
7.58
7.56
7.46
7.45
7.45
7.44
7.44
7.43
7.33
7.32
7.31
7.31
7.30
7.30
7.29
7.29
4.07
4.05
4.03
4.02
1.61
1.59
Me
Ph
F3C
4.02
2.02
3.00
1.00
3.02
8.0
7.5
7.0
6.5
6.0
5.5
5.0
4.5
4.0
3.5
3.0
2.5
2.0
1.5
1.0
0.5
0.0
f1 (ppm)

3x

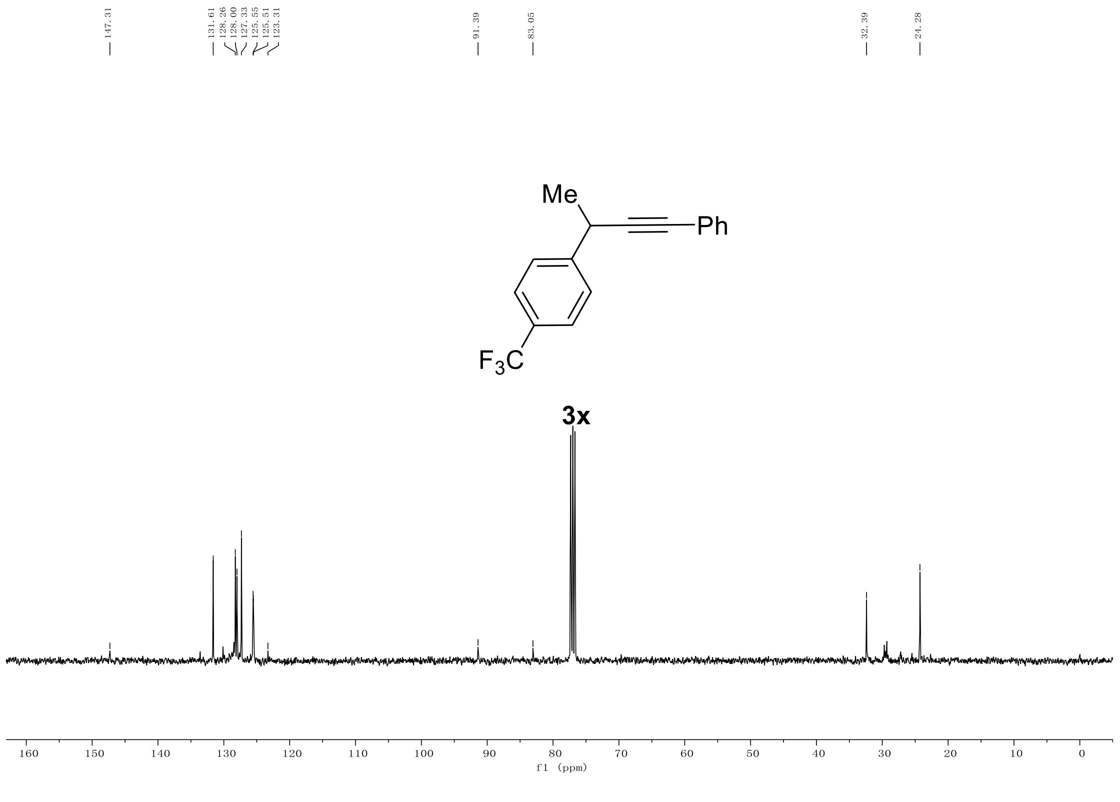
147.31
131.61
128.26
128.00
127.33
125.55
125.51
123.31
91.39
83.05
32.39
24.28
Me
Ph
F3C
160
150
140
130
120
110
100
90
80
70
60
50
40
30
20
10
0
f1 (ppm)

3x

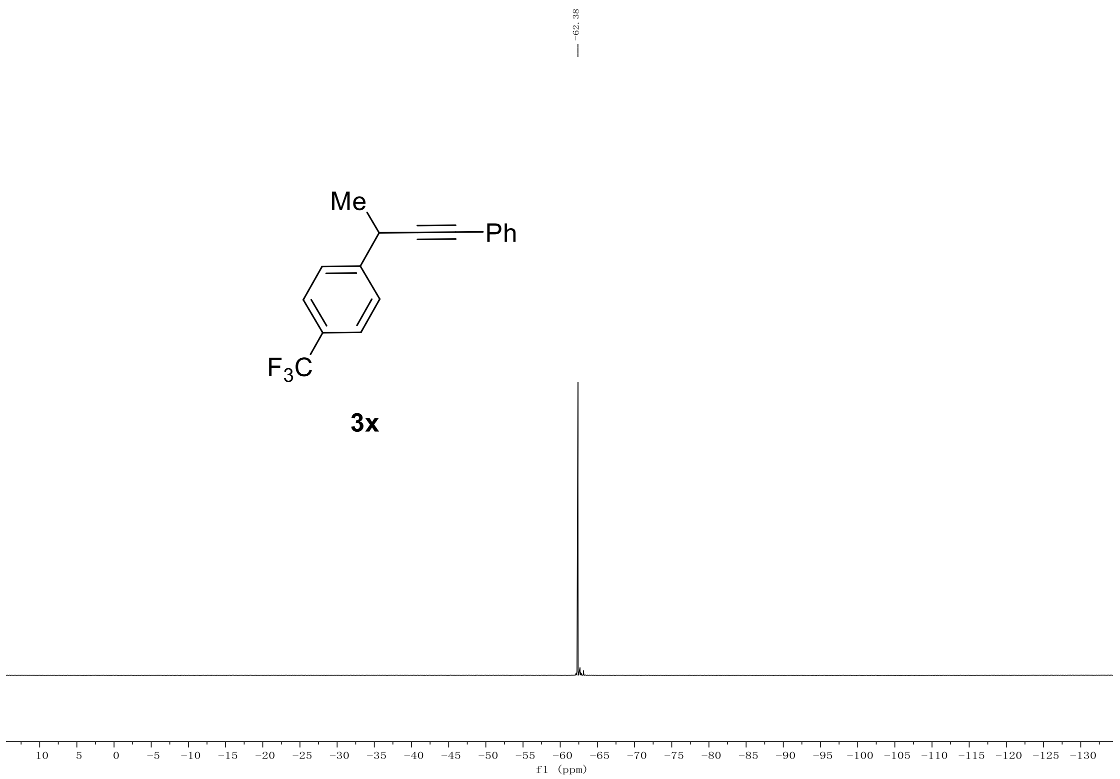

-62.38
Me
Ph
F3C
3x
f1 (ppm)

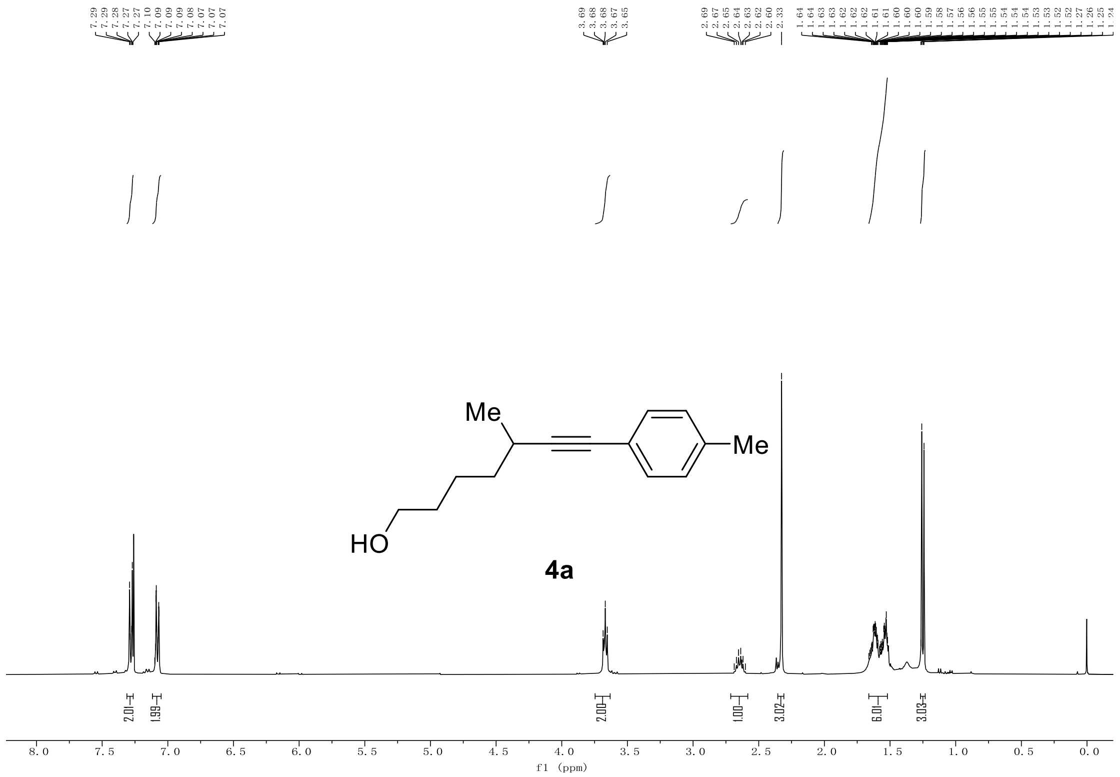

Me
Me
HO
4a
2.01
1.99
2.00
1.00
3.02
6.01
3.03
f1 (ppm)

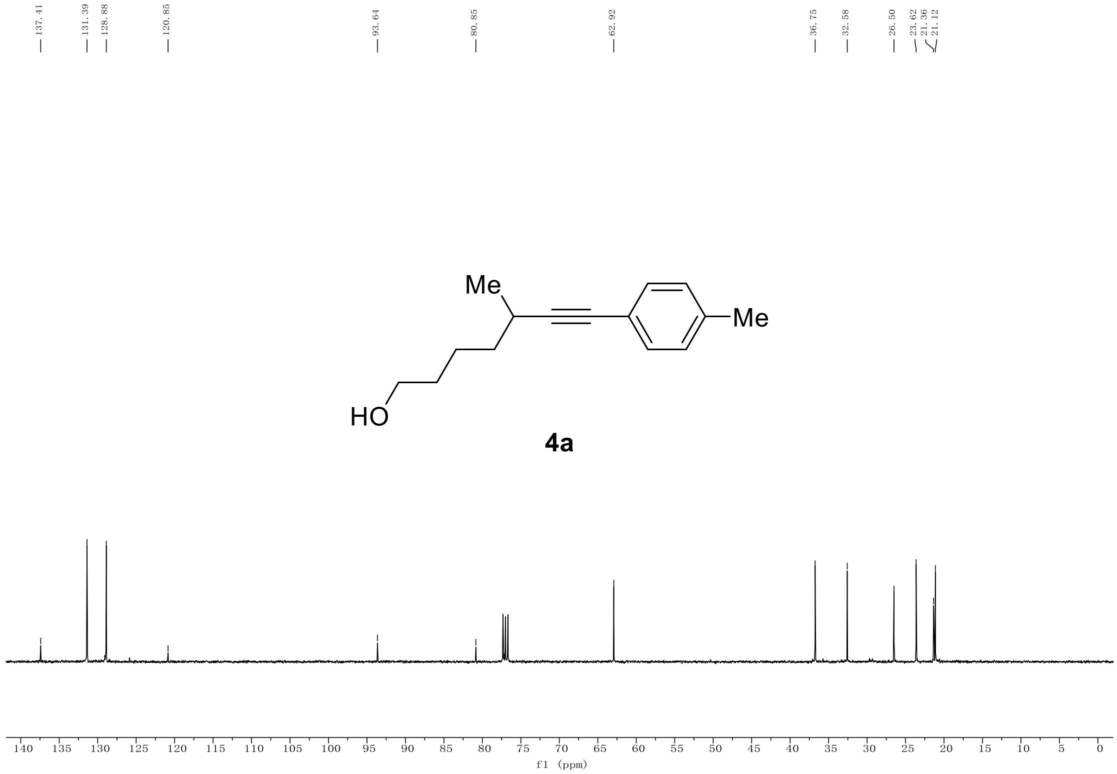
137.41
131.39
128.88
120.85
93.64
80.85
62.92
36.75
32.58
26.50
23.62
21.36
21.12
Me
Me
HO
f1 (ppm)

4a

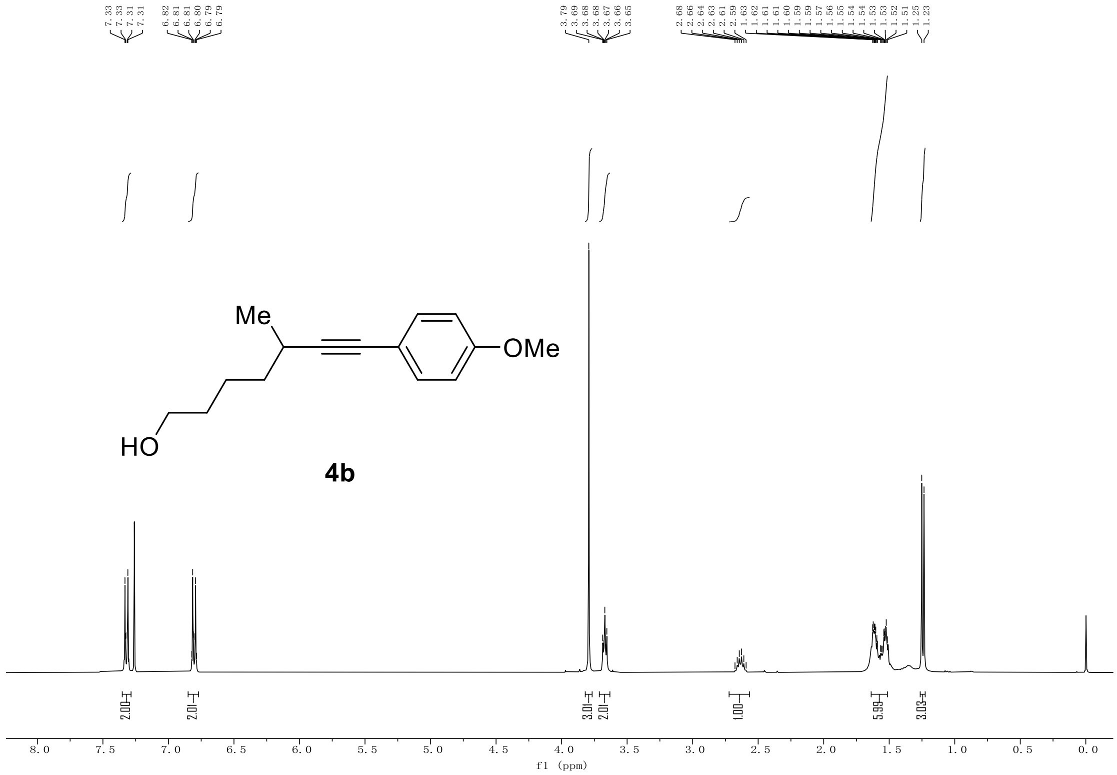
7.33
7.33
7.31
7.31
6.82
6.81
6.81
6.80
6.79
6.79
3.79
3.69
3.68
3.68
3.67
3.66
3.65
Me
OMe
HO
2.00
2.01
3.01
2.01
1.00
5.99
3.03
f1 (ppm)

4b

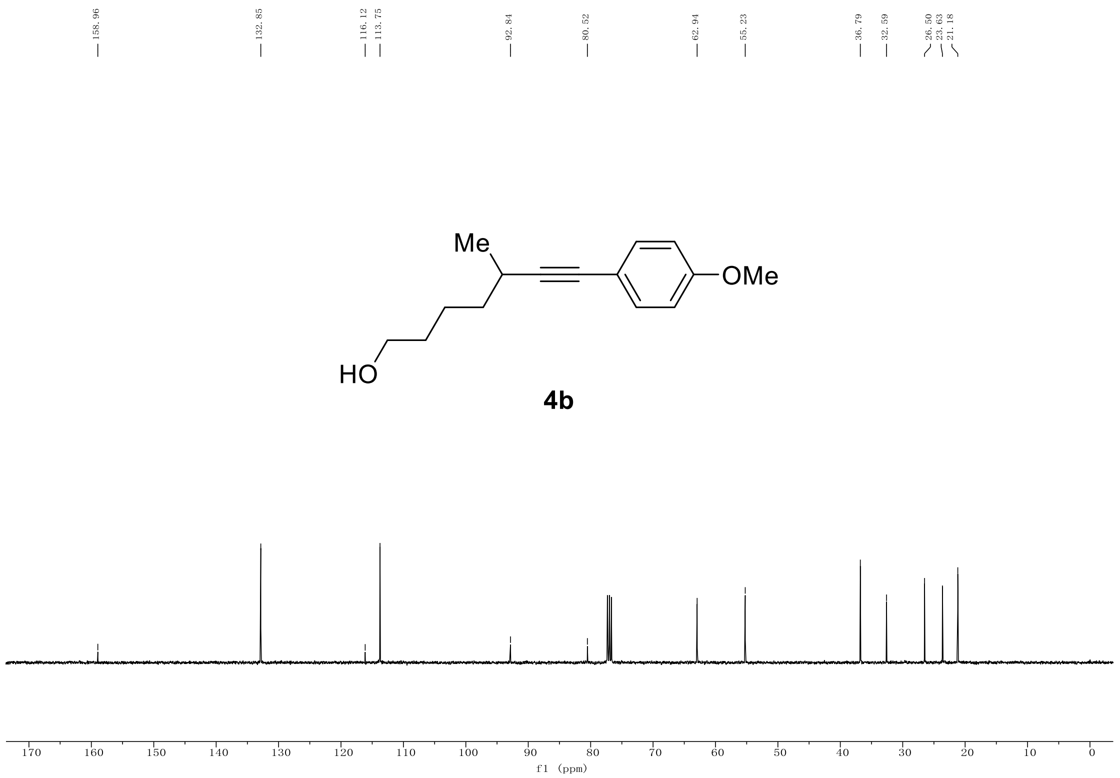
158.96
132.85
116.12
113.75
92.84
80.52
62.94
55.23
36.79
32.59
26.50
23.63
21.18
Me
OMe
HO
4b
170 160 150 140 130 120 110 100 90 80 70 60 50 40 30 20 10 0
f1 (ppm)

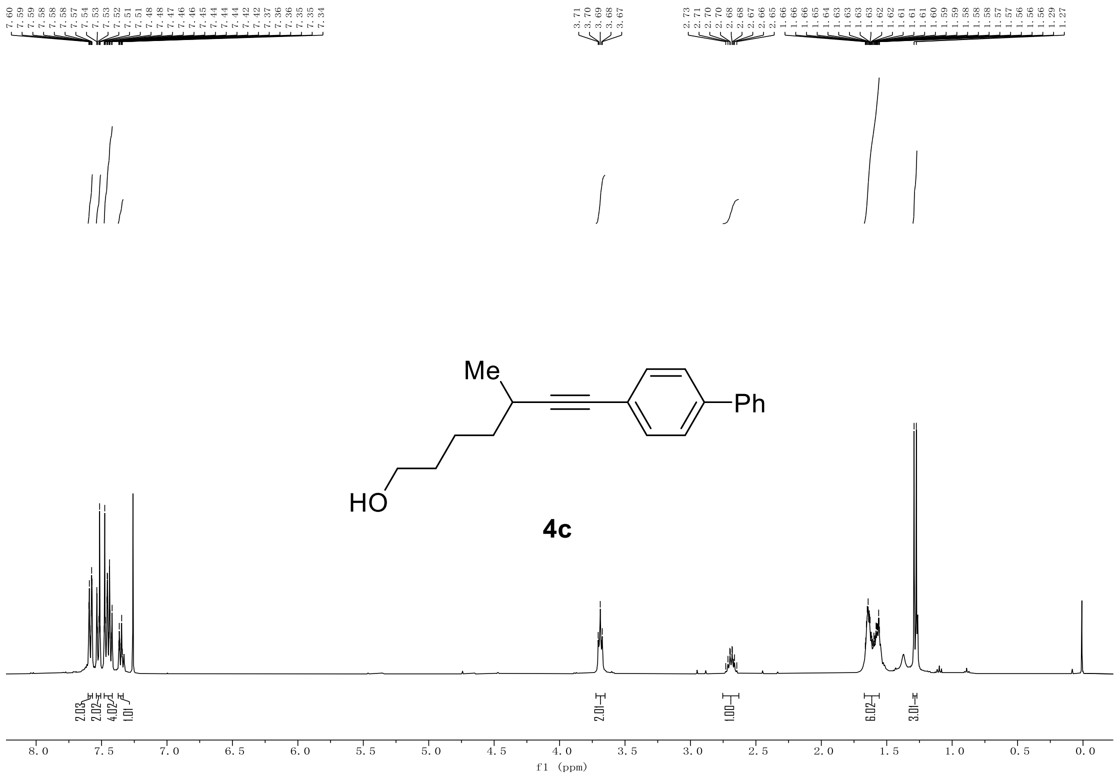
7.60 7.59 7.58 7.58 7.58 7.57 7.54 7.53 7.53 7.52 7.51 7.48 7.47 7.46 7.46 7.45 7.44 7.44 7.42 7.42 7.37 7.36 7.36 7.35 7.35 7.34
3.71 3.70 3.69 3.68 3.67
2.73 2.71 2.70 2.70 2.68 2.68 2.67 2.66 2.66 2.65
1.66 1.66 1.65 1.64 1.63 1.63 1.63 1.63 1.62 1.62 1.61 1.61 1.60 1.59 1.59 1.58 1.58 1.58 1.57 1.57 1.56 1.56 1.29 1.27
Me
Ph
HO
4c
2.03
2.02
4.02
1.01
2.01
1.00
6.02
3.01
8.0 7.5 7.0 6.5 6.0 5.5 5.0 4.5 4.0 3.5 3.0 2.5 2.0 1.5 1.0 0.5 0.0
f1 (ppm)

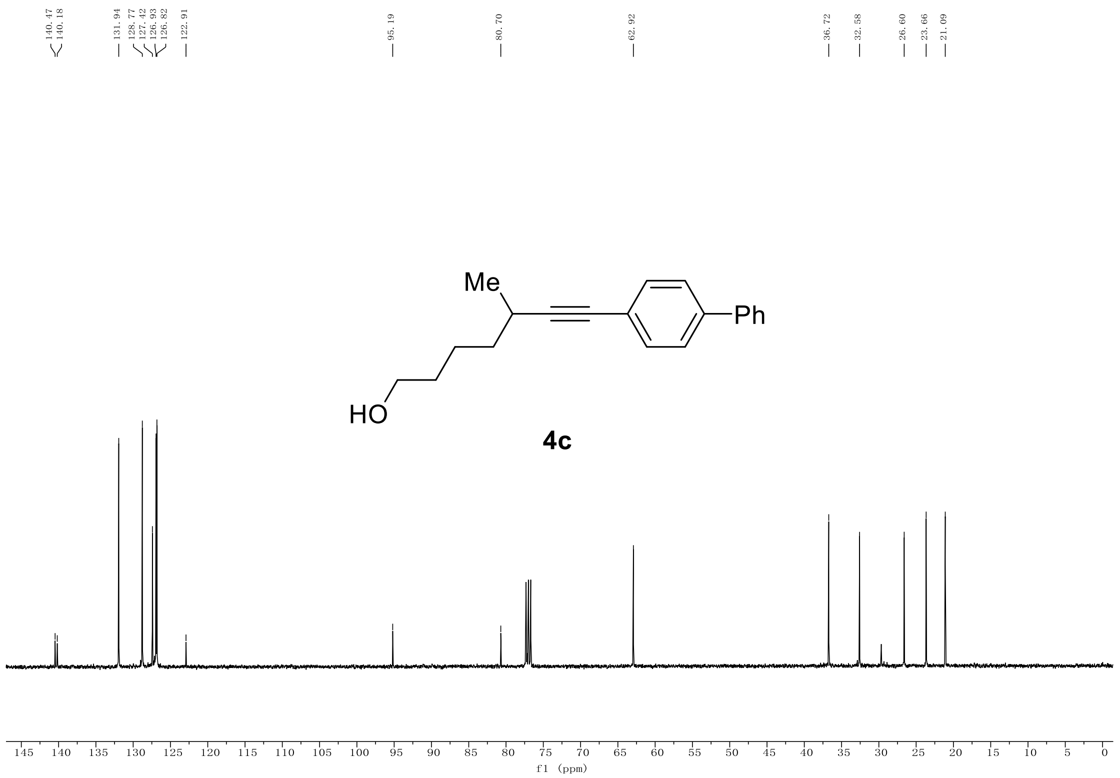
140.47
140.18
131.94
128.77
127.42
126.93
126.82
122.91
95.19
80.70
62.92
36.72
32.58
26.60
23.66
21.09
Me
Ph
HO
4c
145 140 135 130 125 120 115 110 105 100 95 90 85 80 75 70 65 60 55 50 45 40 35 30 25 20 15 10 5 0
f1 (ppm)

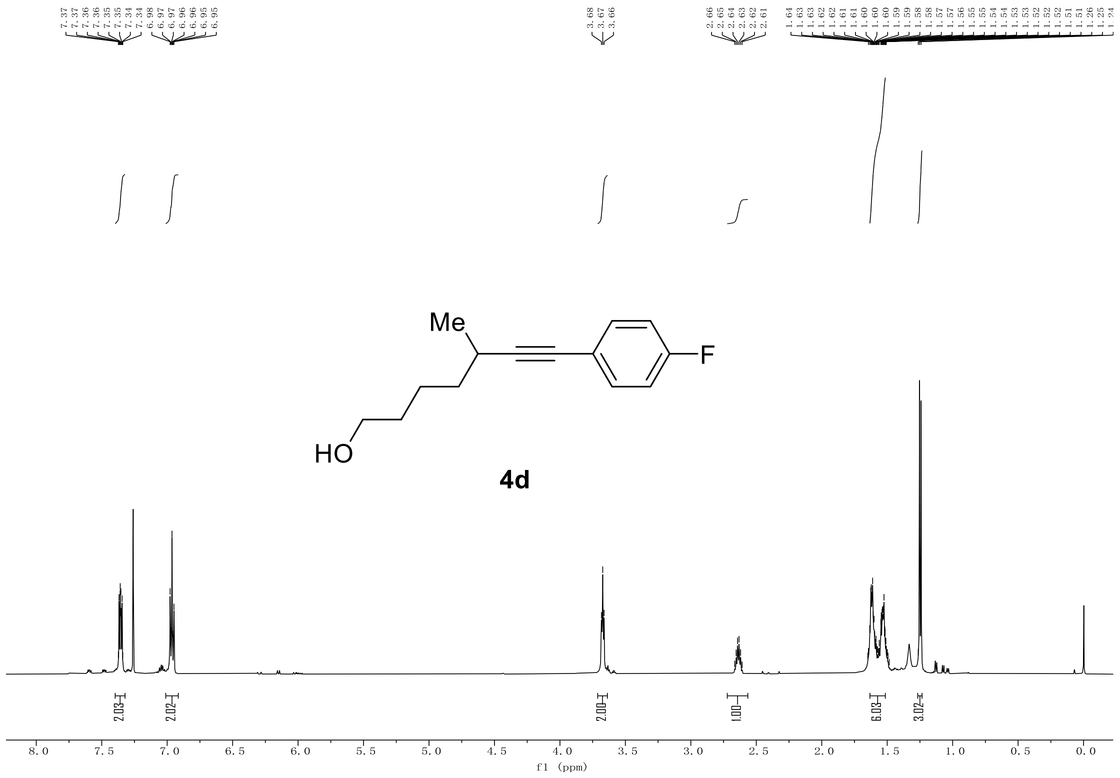
7.37 7.37 7.36 7.35 7.35 7.34 7.34 6.98 6.97 6.97 6.96 6.96 6.95 6.95
3.68 3.67 3.66
2.66 2.65 2.64 2.63 2.62 2.61
1.64 1.63 1.63 1.62 1.62 1.61 1.61 1.60 1.60 1.60 1.59 1.59 1.58 1.58 1.57 1.56 1.55 1.55 1.54 1.54 1.53 1.53 1.52 1.52 1.52 1.51 1.51 1.26 1.25 1.24
Me
F
HO
4d
2.03
2.02
2.00
1.00
6.03
3.02
8.0 7.5 7.0 6.5 6.0 5.5 5.0 4.5 4.0 3.5 3.0 2.5 2.0 1.5 1.0 0.5 0.0
f1 (ppm)

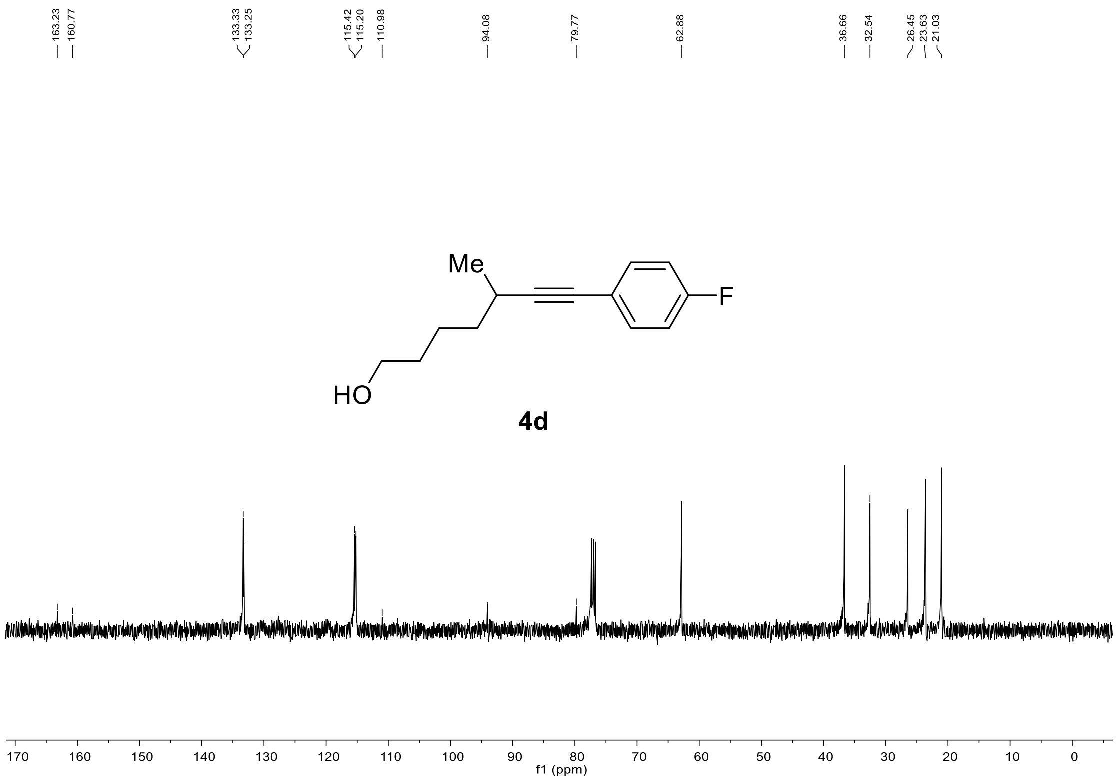
163.23
160.77
133.33
133.25
115.42
115.20
110.98
94.08
79.77
62.88
36.66
32.54
26.45
23.63
21.03
Me
F
HO
4d
f1 (ppm)

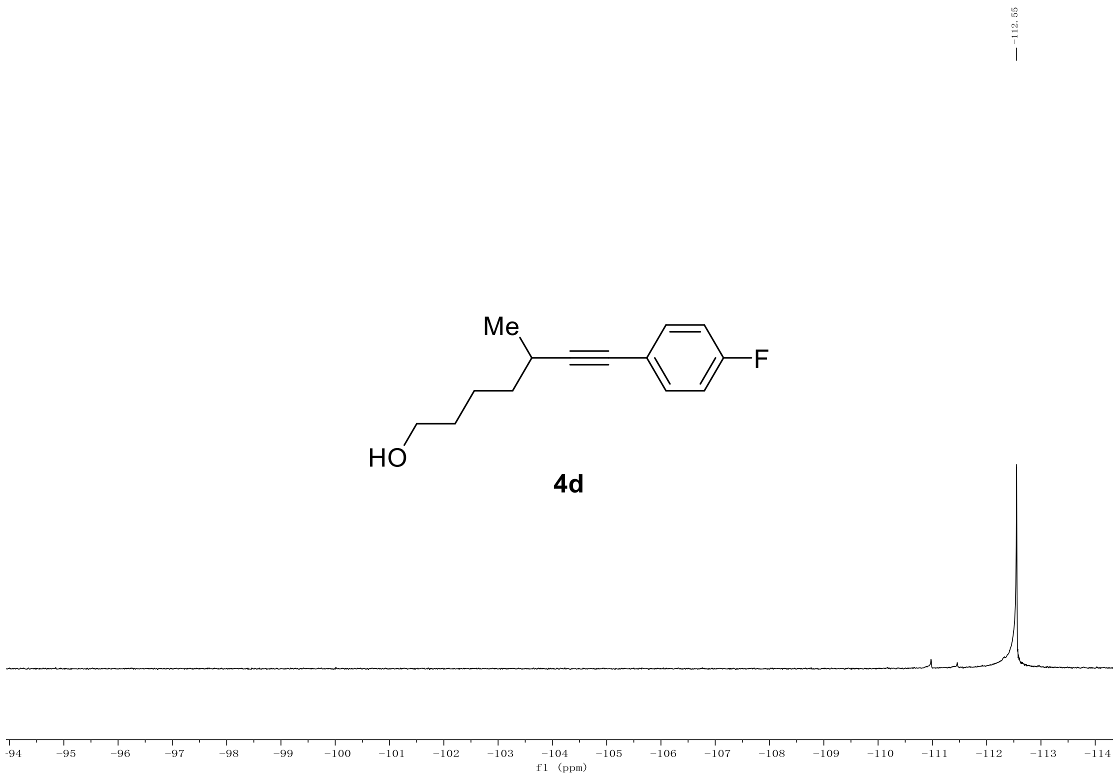
-112.55
Me
F
HO
4d
f1 (ppm)

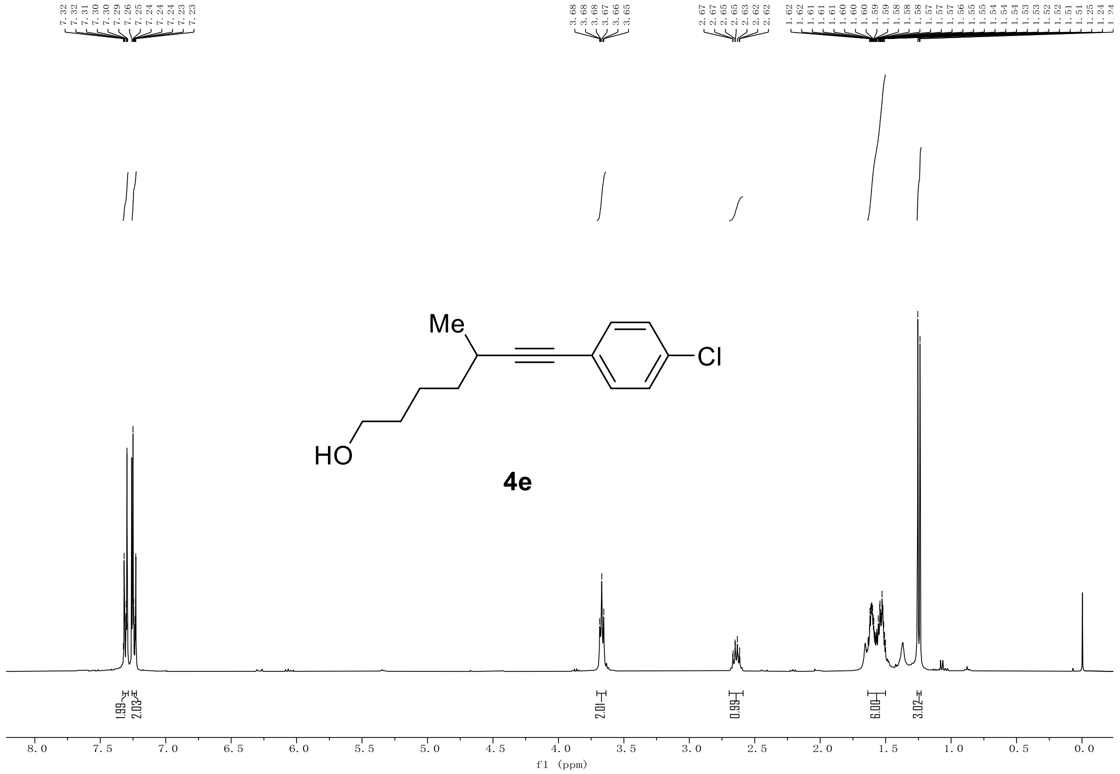
Me
Cl
HO
4e
1.99
2.03
2.01
0.99
6.00
3.02
f1 (ppm)

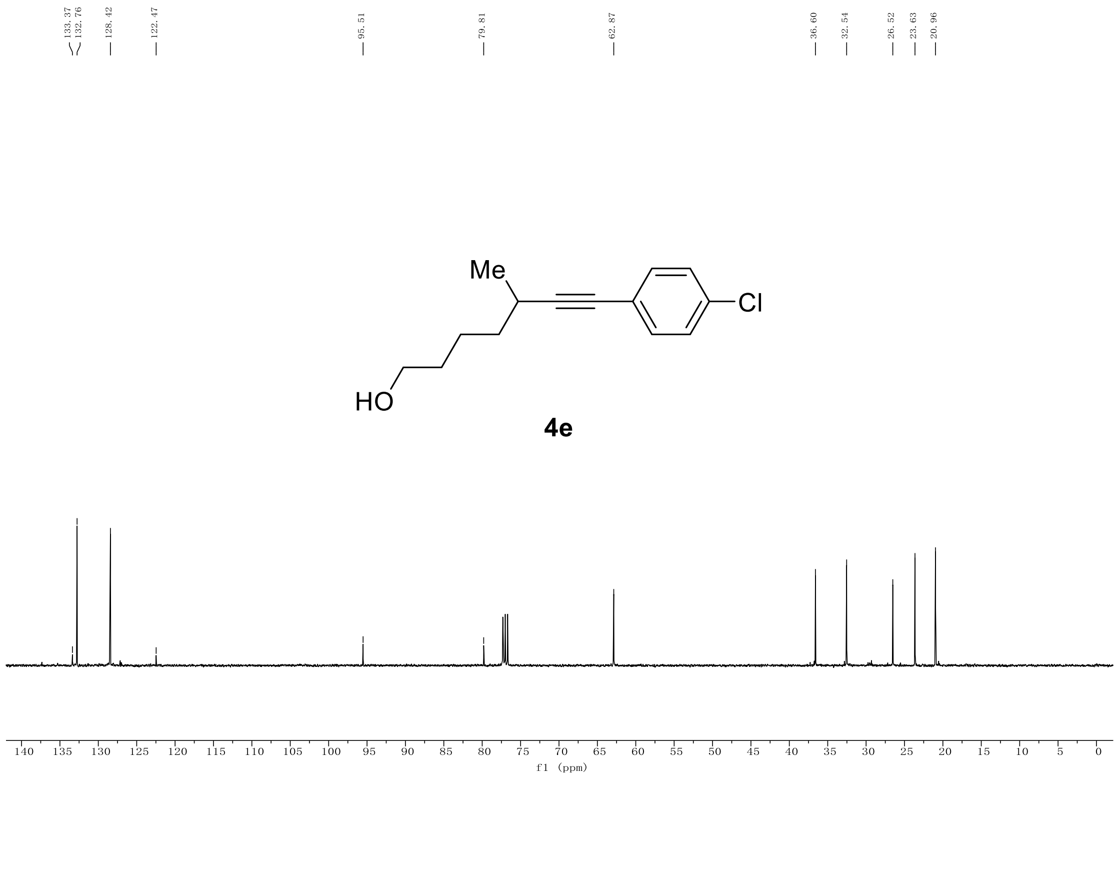
133.37
132.76
128.42
122.47
95.51
79.81
62.87
36.60
32.54
26.52
23.63
20.96
Me
Cl
HO
4e
f1 (ppm)

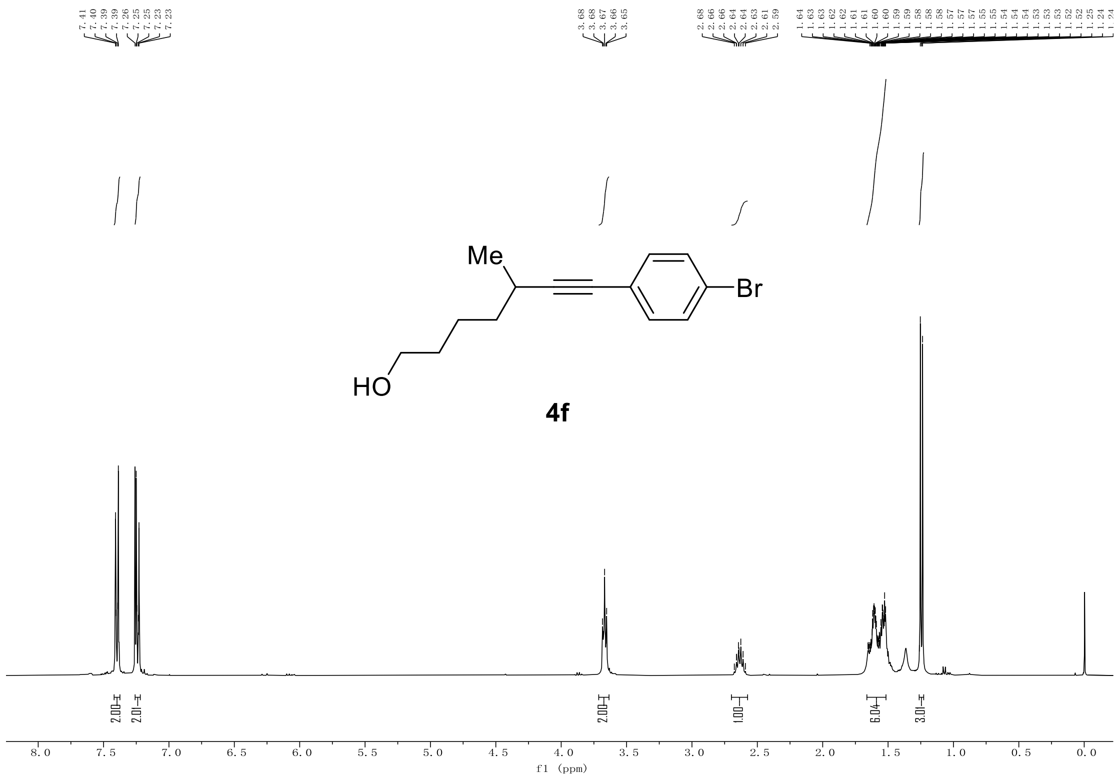
Me
Br
HO
4f
f1 (ppm)

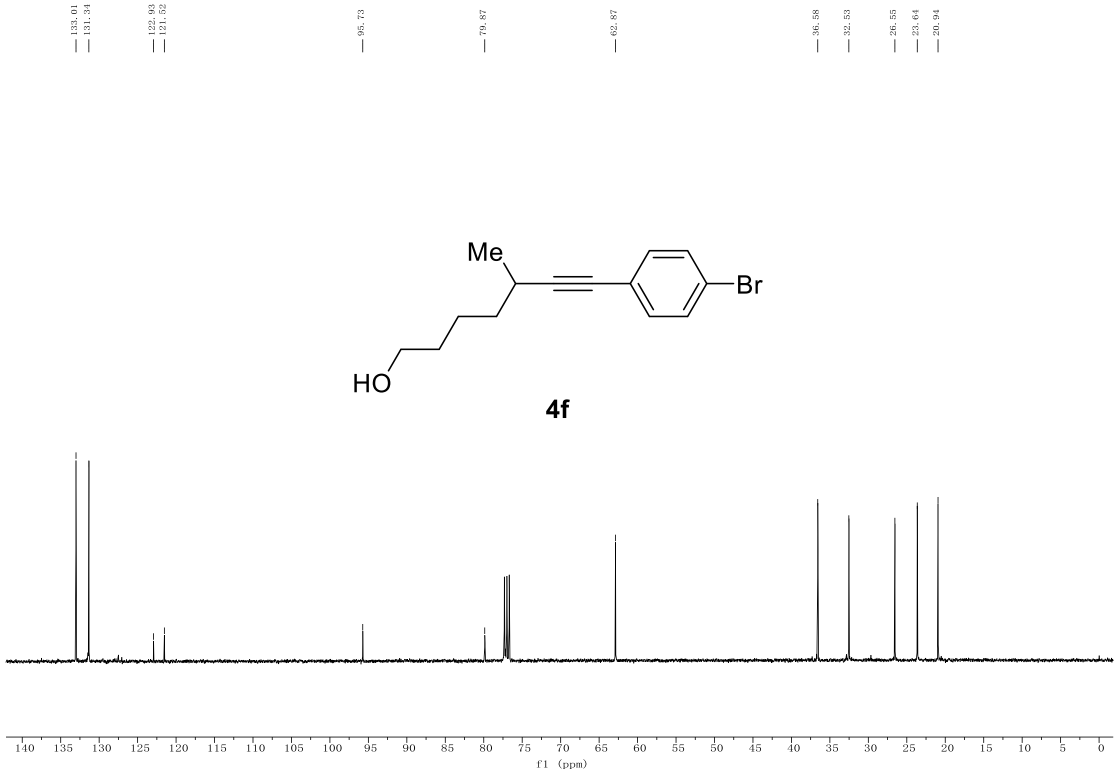
Me
Br
HO
4f
f1 (ppm)

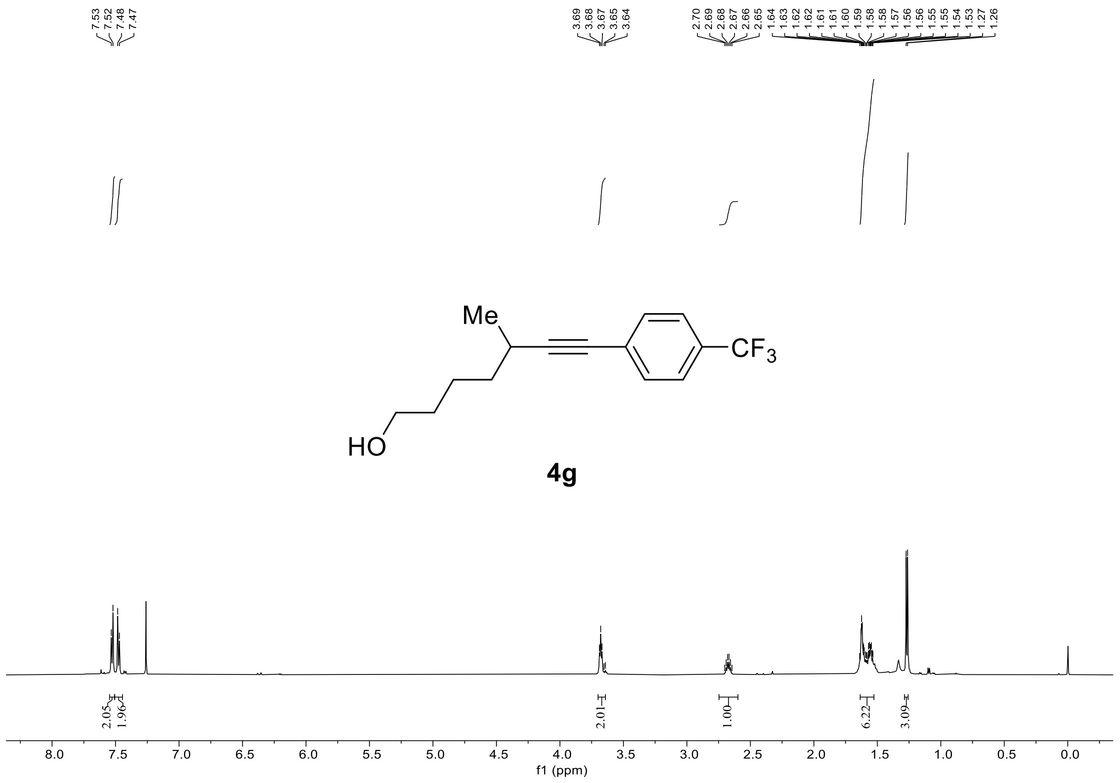

7.53 7.52 7.48 7.47
3.69 3.68 3.67 3.65 3.64
2.70 2.69 2.68 2.67 2.66 2.65 1.64 1.63 1.62 1.62 1.61 1.61 1.60 1.59 1.58 1.58 1.57 1.56 1.56 1.55 1.55 1.54 1.53 1.27 1.26
Me
CF_3
HO
2.05 1.96 2.01 1.00 6.22 3.09
8.0 7.5 7.0 6.5 6.0 5.5 5.0 4.5 4.0 3.5 3.0 2.5 2.0 1.5 1.0 0.5 0.0
f1 (ppm)

4g

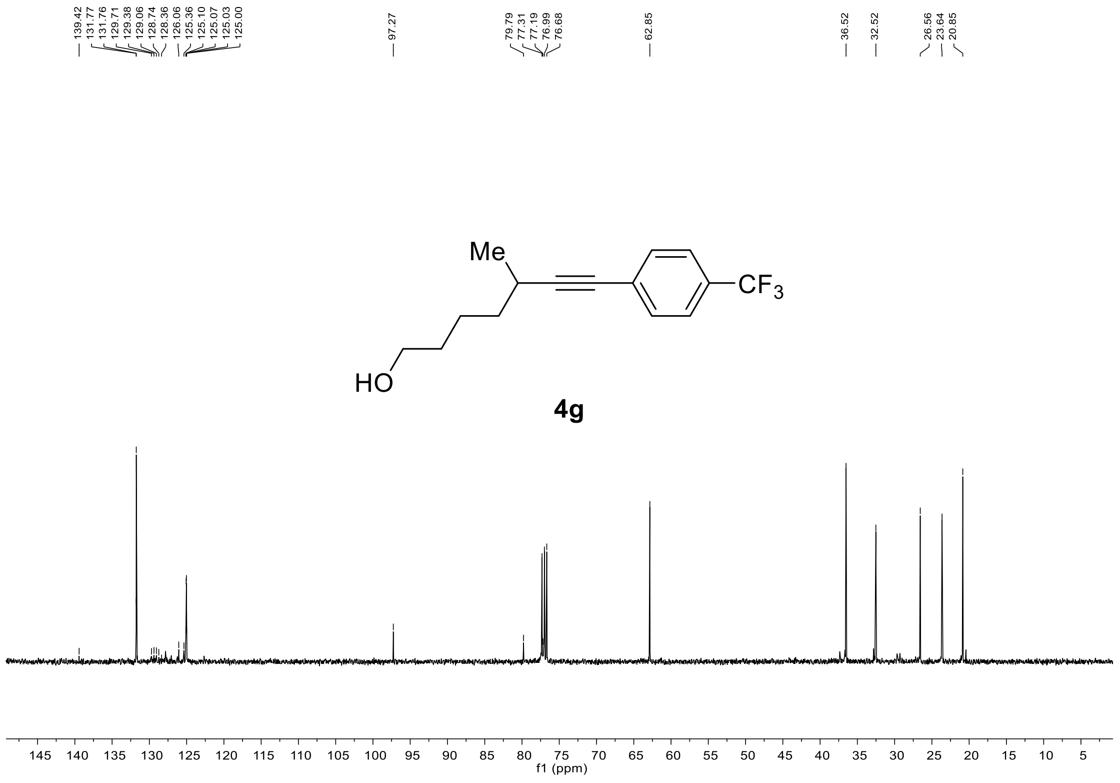

139.42 131.77 131.76 129.71 129.38 129.06 128.74 128.36 126.06 125.36 125.10 125.07 125.03 125.00
97.27
79.79 77.31 77.19 76.99 76.68
62.85
36.52 32.52
26.56 23.64 20.85
Me
CF_3
HO
145 140 135 130 125 120 115 110 105 100 95 90 85 80 75 70 65 60 55 50 45 40 35 30 25 20 15 10 5
f1 (ppm)

4g

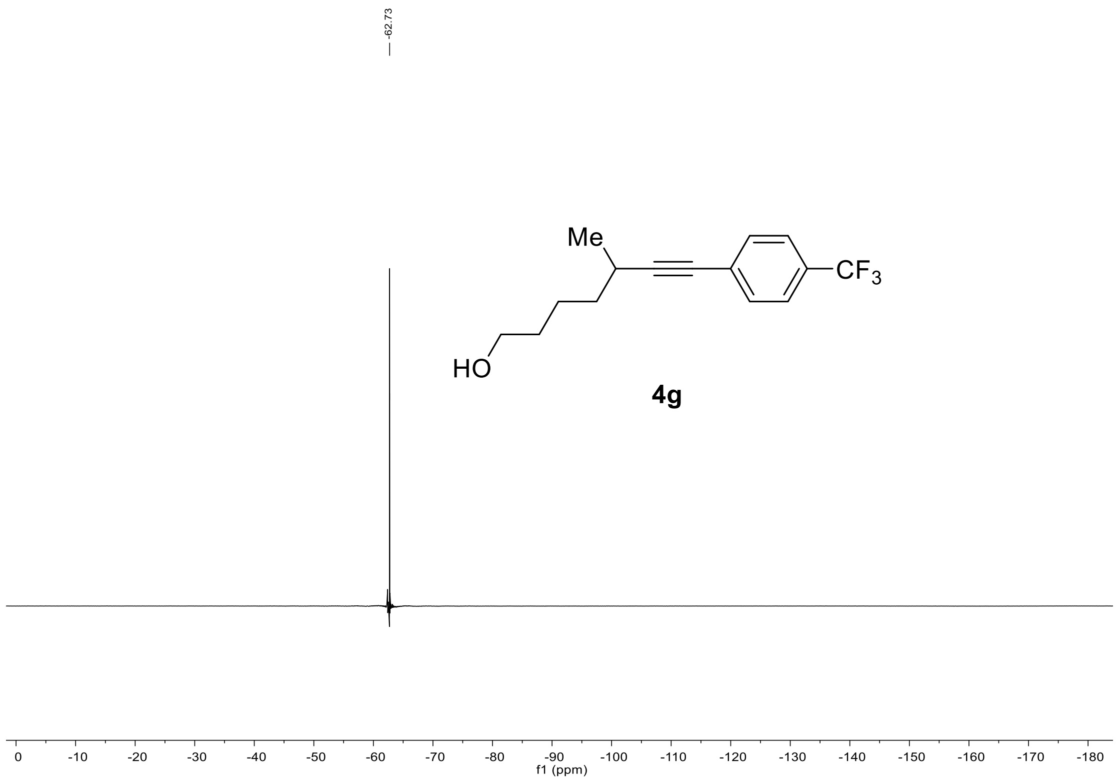

-62.73
Me
CF3
HO
4g
0 -10 -20 -30 -40 -50 -60 -70 -80 -90 -100 -110 -120 -130 -140 -150 -160 -170 -180
f1 (ppm)

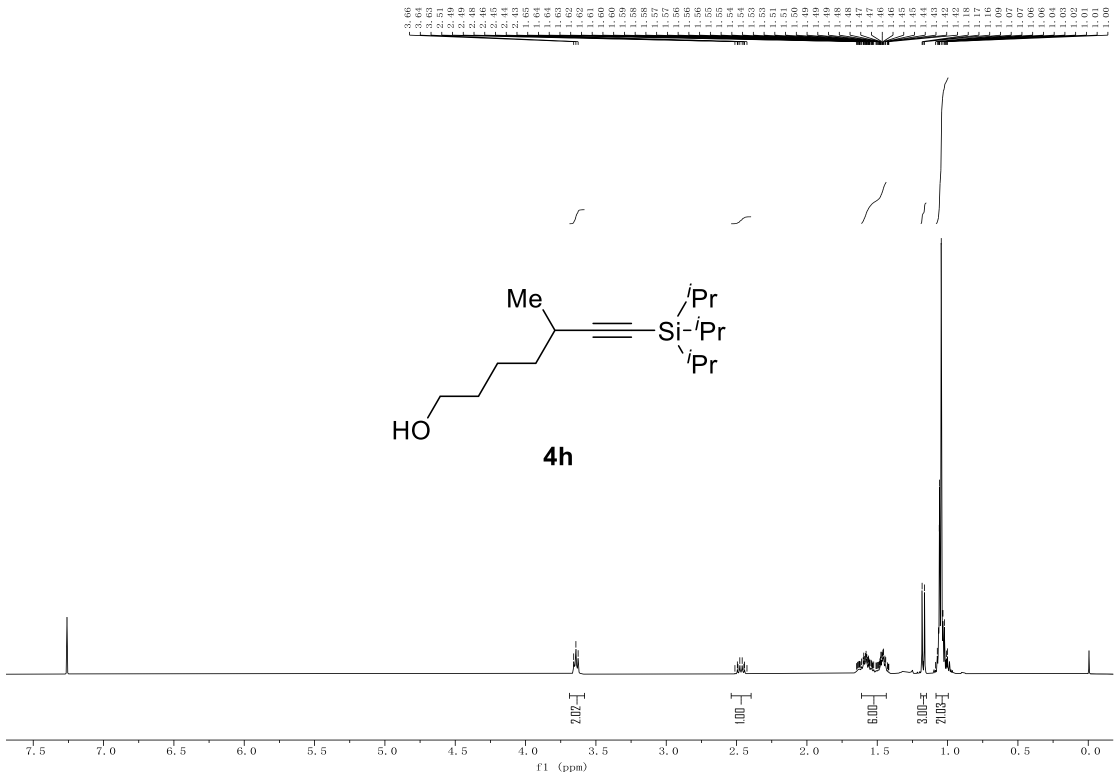

3.66 3.64 3.63 2.51 2.49 2.49 2.48 2.46 2.45 2.44 2.43 1.65 1.64 1.63 1.62 1.62 1.61 1.60 1.60 1.59 1.58 1.58 1.57 1.57 1.56 1.56 1.55 1.55 1.54 1.54 1.53 1.53 1.51 1.51 1.50 1.49 1.49 1.49 1.48 1.48 1.47 1.47 1.46 1.46 1.45 1.45 1.44 1.43 1.42 1.42 1.18 1.17 1.16 1.09 1.07 1.06 1.06 1.04 1.03 1.02 1.01 1.00
Me
Si
iPr
iPr
iPr
HO
4h
2.02
1.00
6.00
3.00
21.03
7.5 7.0 6.5 6.0 5.5 5.0 4.5 4.0 3.5 3.0 2.5 2.0 1.5 1.0 0.5 0.0
f1 (ppm)

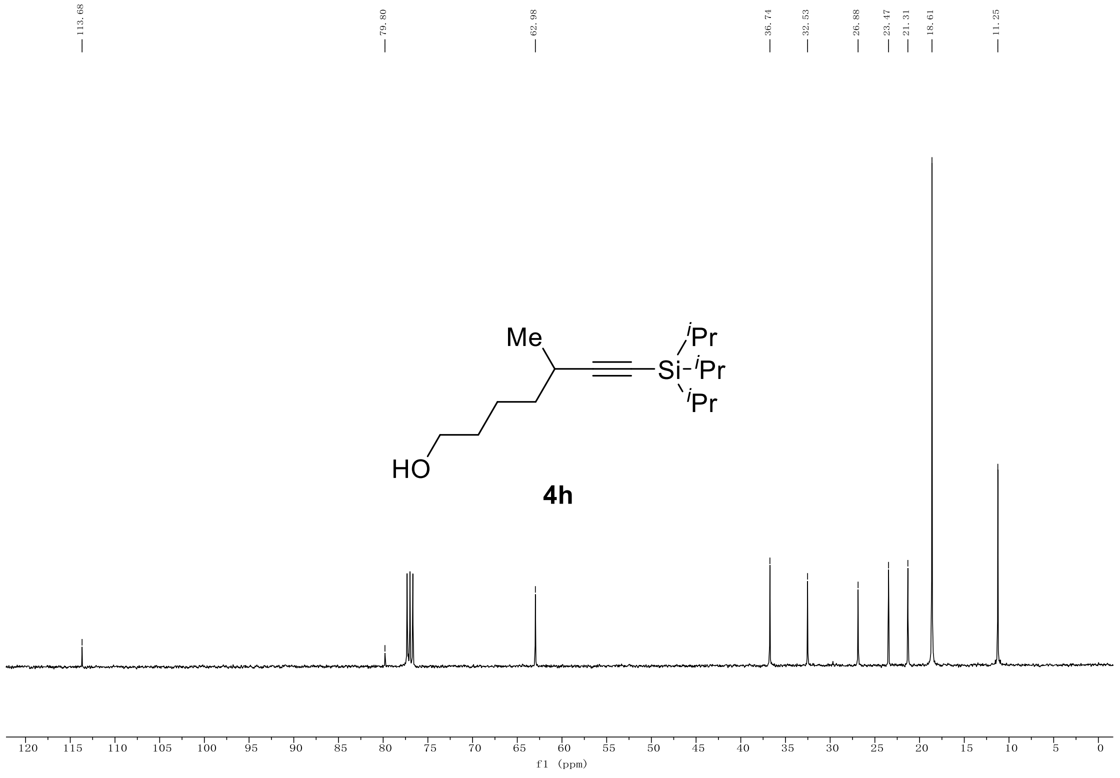

113.68
79.80
62.98
36.74
32.53
26.88
23.47
21.31
18.61
11.25
Me
Si
iPr
iPr
iPr
HO
4h
120 115 110 105 100 95 90 85 80 75 70 65 60 55 50 45 40 35 30 25 20 15 10 5 0
f1 (ppm)
